# Unique molecular and functional features of extramedullary hematopoietic stem and progenitor cell reservoirs in humans

**DOI:** 10.1182/blood.2021013450

**Published:** 2022-06-09

**Authors:** Nicole Mende, Hugo P. Bastos, Antonella Santoro, Krishnaa T. Mahbubani, Valerio Ciaurro, Emily F. Calderbank, Mariana Quiroga Londoño, Kendig Sham, Giovanna Mantica, Tatsuya Morishima, Emily Mitchell, Maria Rosa Lidonnici, Fabienne Meier-Abt, Daniel Hayler, Laura Jardine, Abbie Curd, Muzlifah Haniffa, Giuliana Ferrari, Hitoshi Takizawa, Nicola K. Wilson, Berthold Göttgens, Kourosh Saeb-Parsy, Mattia Frontini, Elisa Laurenti

**Affiliations:** 1 Department of Haematology, University of Cambridge, Cambridge Biomedical Campus, Cambridge, United Kingdom; 2 Wellcome-MRC Cambridge Stem Cell Institute, University of Cambridge, Cambridge Biomedical Campus, Cambridge, UK; 3 Department of Haematology and Cambridge NIHR Biomedical Research Centre, Biomedical Campus, University of Cambridge, Cambridge, UK; 4 Laboratory of Stem Cell Stress, International Research Centre for Medical Sciences, and Centre for Metabolic Regulation of Healthy Aging, Kumamoto University, Kumamoto, Japan; 5 Laboratory of Hematopoietic Stem Cell Engineering, International Research Center for Medical Sciences, Kumamoto University, 860-0811 Kumamoto, Japan; 6 Cancer, Ageing and Somatic Mutation Group, Wellcome Sanger Institute, Hinxton, UK; 7 San Raffaele-Telethon Institute for Gene Therapy (SR-TIGET), IRCCS San Raffaele Scientific Institute, Milan, Italy; 8 Department of Medical Oncology and Hematology, University Hospital Zurich and University of Zurich, Zurich, Switzerland; 9 Institute of Molecular Systems Biology (IMSB), ETH Zurich, Zurich, Switzerland; 10 Institute of Medical Genetics, University of Zurich, Zurich, Switzerland; 11 Biosciences Institute, Newcastle University, Newcastle upon Tyne, UK; 12 Haematology Department, Freeman Hospital, Newcastle-upon-Tyne Hospitals NHS Foundation Trust, Newcastle-upon-Tyne, NE7 7DN, UK; 13 Department of Surgery and Cambridge NIHR Biomedical Research Centre, Biomedical Campus, University of Cambridge, Cambridge, UK; 14 Wellcome Sanger Institute, Wellcome Genome Campus, Hinxton, Cambridge CB10 1SA, UK; 15 Department of Dermatology and NIHR Newcastle Biomedical Research Centre, Newcastle Hospitals NHS Foundation Trust, Newcastle upon Tyne NE2 4LP, UK; 16 Vita-Salute San Raffaele University, Milan, Italy; 17 Institute of Biomedical & Clinical Science, College of Medicine and Health, University of Exeter Medical School, Exeter, UK; 18 National Health Service Blood and Transplant, Cambridge Biomedical Campus, Cambridge, United Kingdom; 19 British Heart Foundation Centre of Excellence, Cambridge Biomedical Campus, Cambridge, United Kingdom

## Abstract

Rare hematopoietic stem and progenitor cell (HSPC) pools outside the bone marrow (BM) contribute to blood production in stress and disease but remain ill-defined. Although non-mobilized peripheral blood (PB) is routinely sampled for clinical management, the diagnosis and monitoring potential of PB HSPCs remains untapped, as no healthy PB HSPC baseline has been reported. Here we comprehensively delineate human extramedullary HSPC compartments comparing spleen, PB and mobilized PB (mPB) to BM using single-cell RNA-seq and/or functional assays. We uncover HSPC features shared by extramedullary tissues and others unique to PB. First, in contrast to actively dividing BM HSPCs, we find no evidence of substantial ongoing hematopoiesis in extramedullary tissues at steady state, but report increased splenic HSPC proliferative output during stress erythropoiesis. Second, extramedullary stem cells/multipotent progenitors (HSC/MPPs) from spleen, PB and mPB share a common transcriptional signature and increased abundance of lineage-primed subsets compared to BM. Third, healthy PB HSPCs display a unique bias towards erythroid-megakaryocytic differentiation. At HSC/MPP level, this is functionally imparted by a subset of phenotypic CD71^+^ HSC/MPPs, exclusively producing erythrocytes and megakaryocytes, highly abundant in PB but rare in other adult tissues. Finally, the unique erythroid-megakaryocytic-skewing of PB is perturbed with age, in essential thrombocythemia and in beta-thalassemia. Collectively, we identify extramedullary lineage-primed HSPC reservoirs that are non-proliferative in situ and report involvement of splenic HSPCs during demand-adapted hematopoiesis. Our data also establish aberrant composition and function of circulating HSPCs as potential clinical indicators of BM dysfunction.

## Introduction

In adults, hematopoiesis occurs in the bone marrow (BM), where over 99% of hematopoietic stem and progenitor cells (HSPCs) reside^1,2^, giving rise to all mature blood cells. Rare HSPCs are also found outside the BM, in extramedullary tissues, such as spleen, lung and liver or circulating in peripheral blood (PB)^3–5^. Yet very little is known about the cellular composition of extramedullary and circulating human HSPCs, their function and their contribution to hematopoiesis in healthy individuals.

In adulthood, HSPC migration and differentiation outside the BM, also called extramedullary hematopoiesis (EMH), is associated with hematopoietic stress and has mostly been studied in mouse models. The spleen is one of the most common sites for EMH in anaemia^6–8^, myeloproliferative disorders^9^, during pregnancy^10–13^ and chronic inflammation/infection^14,15^. Under stress, splenic SCF and CXCL12 attracts BM HSPCs to infiltrate the spleen^10,16,17^. At steady-state, spleen HSPCs comprise BM-derived circulating HSPCs transiently occupying a distinct niche, but likely also long-term residents of this tissue, as shown by parabiosis experiments^18^ and the identification of progenitor cell types found exclusively in spleen^19,6,8^. Phenotypic hematopoietic stem cells (HSCs) are significantly less frequent in mouse and human spleen than in BM, but have similar long-term repopulating capacity^18^ or long-term culture initiating capacity^20^, respectively. Finally, clinical observations suggest differences in EMH in mice and humans, particularly under stress, with mice much more likely to activate splenic erythropoiesis than humans^21^. Overall, the cellular dynamics and molecular regulation of HSPCs inside the spleen remain largely unexplored, especially in humans.

Much research has focused on signals that mobilize BM HSCs into PB^22^. HSPC migration and activity in PB fluctuates following a light-dark cycle^23^, peaking in humans post meridian^24^. In mice, most steady-state circulating PB HSPCs show no long-term repopulating potential^25^, often fail to re-enter the BM^18,26,27^, or change phenotype after their egress^27^. In humans, increased frequencies of CD34^+^ cells in PB are observed in many hematopoietic diseases, notably in sickle cell anemia and b-thalassemia, and in cardiovascular, autoimmune and rheumatological conditions^21,28–34^, but with very few studies providing further resolution of CD34^+^ subsets. It has long been assumed that steady-state PB HSPC composition mirrors that of the BM but no baseline steady-state circulating HSPC composition has been reported to date.

Here we report unique cellular, molecular and functional features of adult human extramedullary HSPCs at steady-state, in G-CSF mobilized peripheral blood (mPB), the most widely used source for clinical HSC transplantation, and in disease. Collectively, we profiled more than 155,000 single CD19^-^CD34^+^ HSPCs by scRNA-seq, 33,000 single HSPCs by CITE-seq and 9,000 single phenotypic hematopoietic stem cells/multipotent progenitors (HSC/MPPs) in functional assays. We define steady-state non-cycling progenitors and HSC/MPP lineage-priming as hallmarks of extramedullary HSPCs. Moreover, HSPCs circulating in PB largely consist of cells committed to erythroid/megakaryocytic differentiation, many of which molecularly akin to HSC/MPPs, in a unique configuration that becomes perturbed with age and disease.

## Methods

### Human samples

Human samples used in this study are summarized in Table S1. Bone marrow (BM), spleen and peripheral blood (PB) from consented deceased organ donors (ODs) with no clinical signs of acute infection, and spleen from hereditary spherocytosis patients were harvested at the Cambridge University Hospitals NHS Trust Addenbrooke’s Hospital in accordance with regulated procedures approved by the relevant Research and Ethics Committees (REC 15/EE/0152 and REC 07/MRE05/44 respectively). PB from living donors (LD-PB, healthy individuals and ET patients) was collected with informed consent by the NHS Blood and Transfusion (NHSBT) Centre in Cambridge from either i) leukocyte cones that are a by-product of platelet apheresis (REC 12/EE/0040); ii) whole blood leukoreduction filters or iii) venipuncture (REC 07/MRE05/44 and REC 18/EE/0199). PB from transfusion-dependent b-thalassemia patients was sampled at Ospedale San Raffaele in Milan with informed consent under TIGET09 protocol. Frozen BM and G-CSF mobilized PB cells were purchased from Lonza and Stem Cell Technologies. Sample preparation, antibody staining, cell sorting and phenotyping by flow and mass cytometry are described in the supplemental methods.

### RNA-sequencing

For 10x scRNA-seq and CITE-seq, up to 30,000 CD19^-^CD34^+^ HSPCs from each tissue and donor (Table S9) were sorted into 300 μl PBS/3%FCS. After centrifugation, cells were resuspended in 47 μl PBS + 0.04% BSA for library prep using the Chromium™ Single Cell 3’ reagents.

Single-cell sorted phenotypic HSC/MPPs from OD1 and OD2, and CD71^+^ PB HSC/MPPs were sequenced with the Smart-seq2 protocol (Table S9) as described by ^35^. This protocol was also used for bulk RNA-sequencing (20 cells) of CD71^-^ and CD71^+^ PB HSC/MPPs. In this case, technical triplicates were sequenced for each population and donor (n=4).

Library preparation and bioinformatics analysis are described in the supplemental methods and Tables S2-4.

### Cell culture assays

Single-cell assays testing for My, Ery, Meg and Ly differentiation were performed as described in ^35^. Single-cell cultures specifically promoting Ery and Meg lineage differentiation were modified from a published protocol ^36^. Bulk colony assays were performed using the Megacult^™^-C kit or MethoCult^™^ (H4034) medium following manufacturer’s instructions (Stem Cell Technologies). Further details on cell culture assays and their analysis are described in the supplemental methods.

### Mice and xenotransplantation assays

Mice of the NOD.Cg-PrkdcscidIl2rgtm1Wjl/SzJ strain (NSG) were obtained from Charles River or bred in-house. Animals were housed in a Specific-Pathogen-Free animal facility. Experiments were conducted under the project license PIL P846C00DB, regulated by the UK Home Office and the Animals (Scientific Procedures) Act 1986 Amendment Regulations 2012 following ethical review by the University of Cambridge Animal Welfare and Ethical Review Body (AWERB).

Xenotransplantation was performed on age-matched female NSG mice as described in supplemental methods.

### Statistical analysis

After examination of data distribution and variance between groups, the appropriate statistical tests were performed with Graph Pad Prism (v7 or higher), R (v3.4.0 or higher), or Python 3.8.6. Statistics for each analysis are described in each relevant section.

## Results

### HSPC composition in extramedullary tissues is skewed towards early progenitors

Human spleen and PB, contain rare and to date poorly characterized phenotypic CD34^+^ HSPCs and HSC/MPPs (supplemental Figure 1A-C). To comprehensively characterize extramedullary HSPC composition at steady state, we performed 10x scRNA-seq on CD19^-^CD34^+^ HSPCs isolated from matched BM, PB and spleen from young organ donors (ODs) with no clinical signs of acute infection and unmatched PB from six healthy volunteers (characteristics in Table S1, supplemental Figure 1D). Given the abundance of CD19^+^CD34^+^ B cell progenitors in the spleen (supplemental Figure 1A), these were excluded from all tissues to ensure profiling of sufficient numbers of all other HSPC subsets.

**Figure 1:**
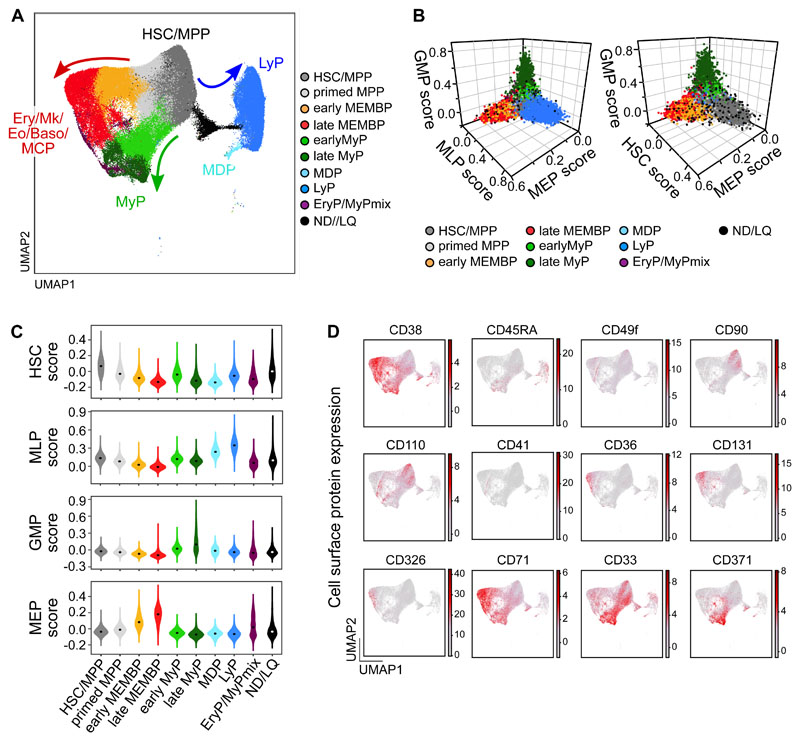
Single cell transcriptomic landscape of human adult HSPCs across medullary and extramedullary hematopoietic tissues. Analysis of 10x Genomics scRNA-seq and CITE-seq data from 117,200 CD19^-^CD34^+^ HSPCs isolated from BM, non-mobilized PB and spleen of adult donors. **(A)** UMAP of the multisite HSPC landscape after exclusion of mature cells (see methods). Clusters were annotated using known lineage and stem cell marker genes found amongst the most differentially expressed genes in each cluster (Table S2a). Clusters with similar cell identity are shown as HSPC groups using different cluster colors. Detailed cluster composition is shown in Supplemental Figure 1F and HSCP grouping is summarized in Table S2c. ND/LQ: cluster of lower quality which identity could not be defined using known marker genes. HSC/MPP: hematopoietic stem cell/multipotent progenitor; MEMBP: Megakaryocyte/erythroid/eosinophil/mast cell/basophil progenitor; MyP: myeloid progenitor; MDP: monocyte/dendritic cell progenitor; LyP: lymphoid progenitor. **(B, C)** 3D-plots (B) and violin plots (C) of lineage- and HSC-scores calculated for each cell using published gene sets enriched in prospectively isolated HSPC subsets^39^ (details see methods). **(D)** CITE-seq data from two BMs (OD3: 9,477 cells, OD4: 12,500 cells) and one spleen (OD4: 11,822 cells). UMAPs highlighting selected surface protein expression across the HSPC landscape.

To generate a reference map of all analyzed hematopoietic tissues, we combined all cells from all donors using the Seurat integration method^37^. Leiden clusters^38^ were annotated based on i) highly expressed marker genes; ii) ‘lineage scores’ derived from published gene sets of highly purified HSPC subsets^39^ and iii) cluster position along a diffusion pseudotime axis (Figure 1A-C, supplemental Figure 1E-I, Table S2a-b). Surface protein expression from CITE-seq data from two donors (Figure 1D) further confirmed cluster annotation and verified that transcriptionally defined HSC/MPPs overlapped with phenotypic HSC/MPPs (CD34^+^CD38^-^CD45RA^-^) with high expression of known HSC markers such as CD90 and CD110.

**Figure 2:**
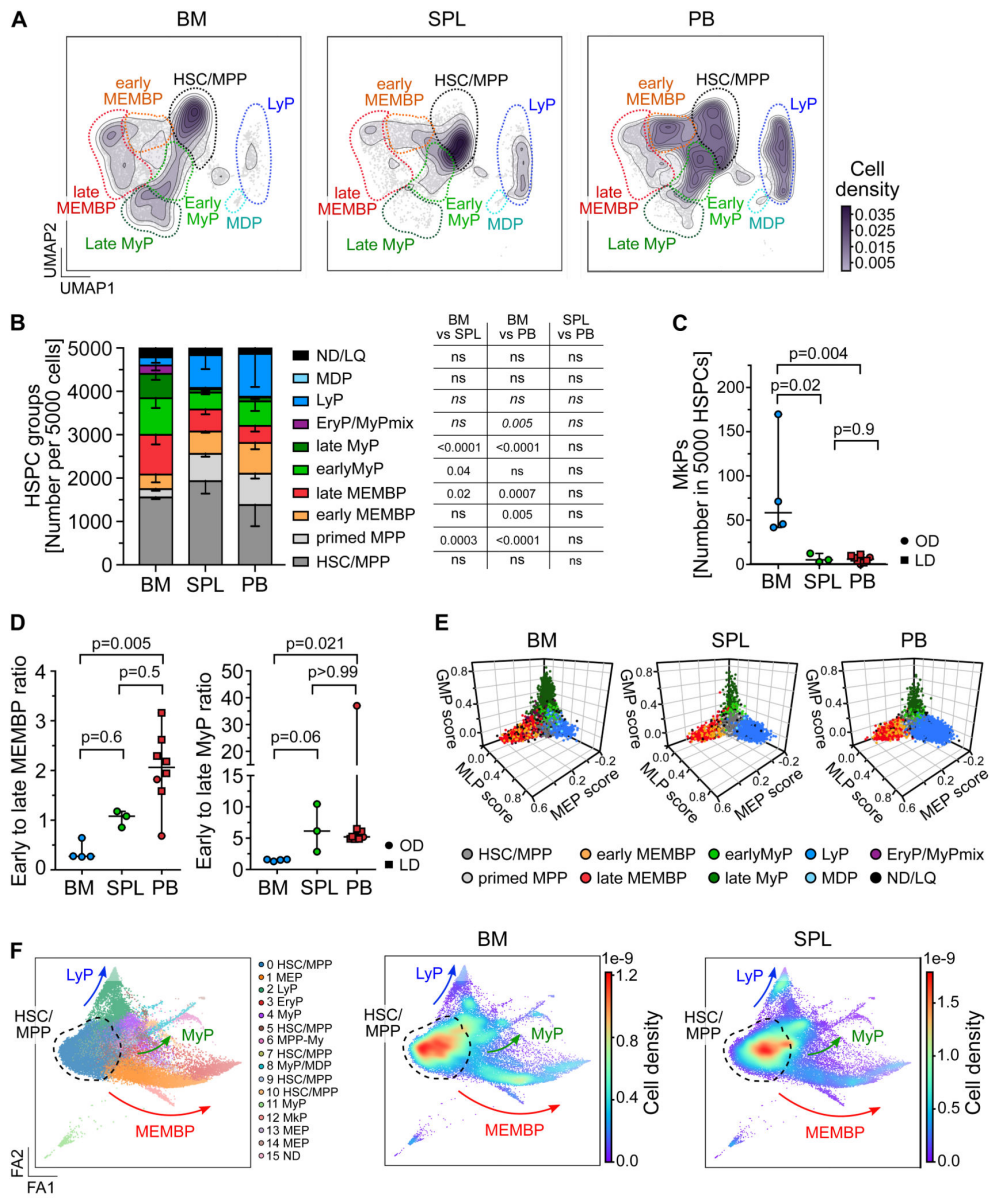
Distinct HSPC composition in BM, spleen and PB. **(A-E)** Analysis of 10x Genomics scRNA-seq data from 117,200 cells, combining all donors but comparing different tissues (BM: 34,967 cells; SPL: 22,068 cells; PB: 60,165 cells). **(A)** 2D kernel density of cells across the UMAP coordinates of each tissue, displayed as contours filled by a color gradient. **(B)** Bar graph of the relative composition of HSPC groups in BM, spleen and PB. Each group was defined as shown in Figure 1A. Mean ± SD is shown. **(C)** Relative number of MkPs (cluster 22) in each tissue. **(D)** The ratio of early to late progenitors of the MEMB (left) or My (right) branch is shown. Kruskal-Wallis; Dunn’s multiple comparison test. **(E)** 3D plots show lineage scores as in Figure 1B for each tissue. **(F)** Force Directed Graph computed using CITE-seq protein data of two BM (OD3: 9,477 cells; OD4: 12,500 cells) and one spleen (OD4: 11,822 cells). Left: Leiden clusters as annotated based on known surface marker expression (see Supplemental Figure 2D). Right: Density visualization of the distinct cell distributions for each tissue in different areas of the landscape. **B-C)** One-way ANOVA with post hoc Tukey test, except for LyP and EryP/MyPmix clusters (not normally distributed, italic text), for which a Kruskal-Wallis test with Dunn’s multiple comparison was used. ns; p>0.05. **C-D)** Median ± 95% confidence interval is shown. LD: living donor, OD: organ donor, SPL: spleen.

Considering that CD19^+^CD34^+^ B cell progenitors were purposedly not profiled here, our multi-tissue HSPC landscape overall resembles that described for human HSPC hierarchies in fetal, neonatal and adult life^40–44^. Strikingly, spleen and PB displayed HSPC compositions clearly distinct from BM in the HSC/MPP compartment and in the committed progenitor branches, as shown by analysis of i) cluster independent cellular distributions (Figure 2A, supplemental Figure 2A); ii) normalized cell counts for each cluster (Table S2c) or iii) HSPC group (Figure 2B). Inter-individual variability in cluster abundance was highest in PB (supplemental Figure 2B), but comparable to that previously reported for healthy donor BM^40^.

BM contained more myeloid (My, Figure 2B) and megakaryocyte progenitors (MkP, Figure 2C) than extramedullary tissues, with the former having significantly higher Meg-priming scores than extramedullary MkPs (supplemental Figure 2C). Spleen and PB harbored significantly less late progenitors of the My and Megakaryocyte Erythroid Mast cell and Basophil (MEMB) lineage than BM (Figure 2A-B), resulting in a marked shift in the ratios of early-to-late progenitors between tissues (Figure 2D). In addition, ‘primed MPPs’, a subset of HSC/MPPs with marked My or Ery/Meg lineage-priming, were significantly enriched at extramedullary sites (Figure 2B). All specific features of progenitor composition in extramedullary tissues were confirmed in a cluster independent manner i) using the lineage-scores (Figure 2E), ii) building an embedding based exclusively on 198 protein markers (done for 2 BMs and 1 spleen, Figure 2F, supplemental Figure 2D-E, Table S2d-e), iii) comparing spleen and PB data to the benchmark Human Cell Atlas BM dataset (HCA)^40^ (see Methods, supplemental Figure 2F-H, Table S2f). All extramedullary features were also observed using different QC parameters (not shown), upon regression of cell cycle genes (Table S2g, Figure S3A-F), and with an independent batch correction method (ComBat^45^, Table S2h, Figure S3G-H). In conclusion, HSPC composition at extramedullary sites markedly differs from that of BM.

### Minimal in situ proliferation of erythroid and myeloid progenitors at extramedullary sites

**Figure 3:**
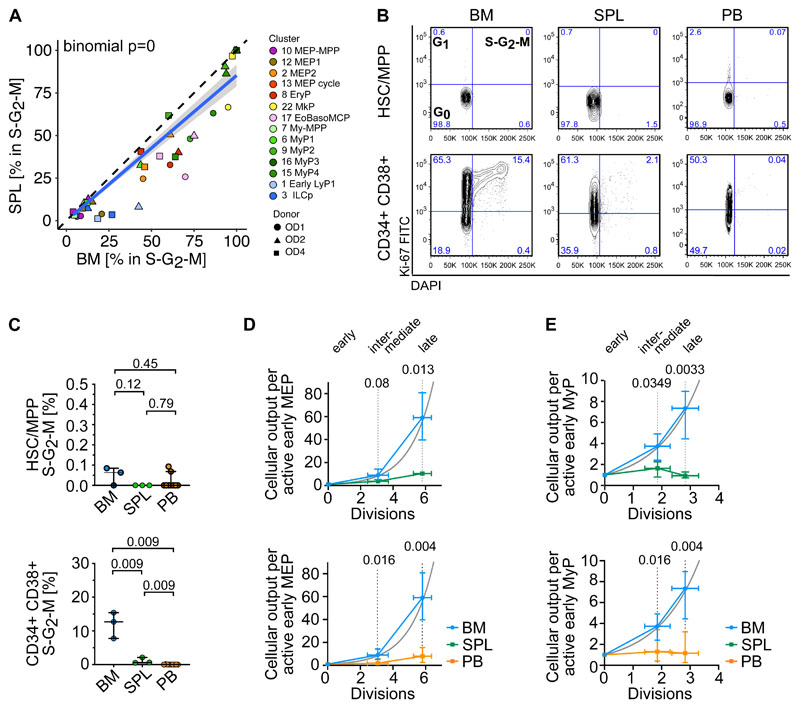
Low proliferation of progenitors in extramedullary tissues compared to BM. **(A,D,E)** Analysis of 10x Genomics scRNA-seq data from 117,200 cells, combining all donors but comparing different tissues. **(A)** Percentage of cells in S-G_2_-M phase (assigned by cell cycle phase scoring as described by ^72^) in matched BM and spleen (left) from the same donor for each indicated progenitor cluster. Two-sided exact binomial test. **(B)** Representative flow cytometry plots of BM (left), spleen (middle) and PB (right) CD19^-^CD34^+^CD38^-^CD45RA^-^ HSC/MPPs (top row) or CD19^-^CD34^+^CD38^+^ progenitor cells (bottom row) in G_0_ (Ki-67^-^DAPI^-^), G_1_ (Ki-67^+^DAPI^-^) and S-G_2_-M (Ki-67^+^DAPI^+^) cell cycle phases. **(C)** Frequency of phenotypic HSC/MPPs (left) or CD19^-^CD34^+^CD38^+^ progenitor cells (right) from each tissue in S-G_2_-M phase (Ki-67^+^DAPI^+^) assessed by flow cytometry. Median ± 95% confidence interval is shown. A two-tailed unpaired t-test was used to compare BM and spleen (normal distribution) and two-tailed Mann-Whitney tests were used to compare BM/spleen with PB (not normally distributed). n=3 non-matched BM and spleen tissues, n=9 PBs measured over 8 experiments. **(D-E)** Estimated cellular output from early to late progenitors of the MEMB (D) and the My branch (E) branch calculated from the number of active cells assuming all divisions are symmetric divisions towards differentiation (details see methods). Grey line: theoretical exponential expansion. Vertical error bars indicate the range observed in the different tissues. Horizontal error bars indicate the standard deviation of the estimated number of divisions for each expansion stage. SPL: spleen.

Given the striking relative lack of late extramedullary progenitors compared to BM, we next investigated the cell cycle and differentiation dynamics of human extramedullary HSPCs. Most progenitor clusters in spleen and PB had significantly decreased proportions of cycling cells (S-G_2_-M phase) compared to BM by transcriptome-based cell cycle assignment (Figure 3A, supplemental Figure 4A, Table S2b). Ki67/DAPI flow cytometry confirmed the almost complete absence of CD19^-^CD34^+^CD38^+^ progenitors in S-G_2_-M at extramedullary sites (Figure 3B-C).

As early progenitors of the MEMB and My branches are overrepresented in extramedullary tissues compared to BM (Figure 2B,D), we estimated the relative numbers of actively proliferating cells in each cluster, and calculated the lower bound expansion along each branch. In BM, progenitor production grew exponentially, at a rate consistent with approximately 6 and 3 differentiating divisions from the earliest to latest stages of the MEMB and My branches respectively (Figure 3D-E), in keeping with active hematopoiesis. In spleen or PB, we modelled very limited progenitor expansion that is incompatible with sustained ongoing hematopoiesis at these sites. In line with the greater proliferative output of BM, division-associated gene sets were enriched in BM early MEMBPs and MyPs compared to extramedullary sites (supplemental Figure 4B, Table S3a-d, S4b-e). Yet, despite not being proliferative in situ, PB MEPs remain responsive to cytokine stimulation *in vitro*, entering cell cycle with similar kinetics as BM MEPs and producing colonies of similar type and size (supplemental Figure 4C-E). In conclusion, whereas extramedullary progenitors retain the capacity to cycle and differentiate, in situ active hematopoiesis is minimal in PB and spleen.

### Most spleen HSC/MPP are lineage-primed

**Figure 4:**
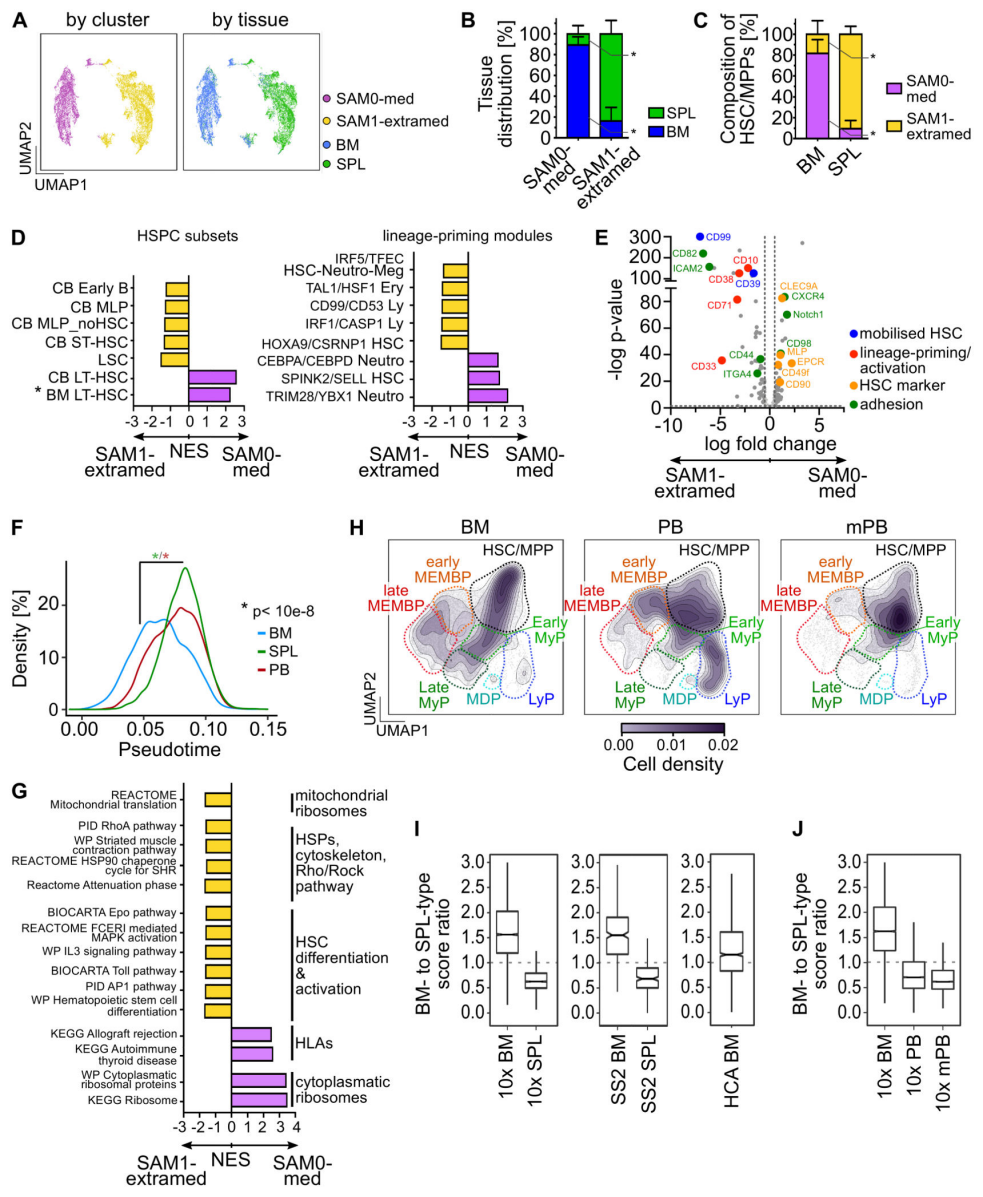
Unique transcriptional and cell surface protein characteristics of extramedullary HSC/MPPs. **(A-E,G)** Analysis of 10x Genomics scRNA-seq data from 16,651 transcriptionally defined HSC/MPPs (sum of clusters 0,4,5,11,21 from Figure 1A) combining matched BM and spleen from the same individuals (OD1: 3,812 cells, OD2: 3,460 cells, OD4: 9,379 cells). **(A)** UMAPs of HSC/MPPs clustered by SAM (k-means=2; top panels colored by SAM cluster, bottom panels by tissue). **(B)** Bar graphs of the proportions of BM and spleen-derived HSC/MPPs in the SAM0-med and SAM1-extramed clusters. **(C)** Proportions of SAM0-med and SAM1-extramed HSC/MPPs in the HSC/MPP space of each tissue. **(D)** Analysis of genes differentially expressed between the SAM0-med (n= 7,068 cells) and SAM1-extramed (n= 9,583 cells) clusters. Pre-ranked GSEA of population-specific signatures (left; CB LT-HSC and ST-HSC from ^47^, BM LT-HSC (unpublished), other from ^39^) and lineage-priming modules (right; from ^43^) comparing SAM0-med with SAM1-extramed HSC/MPPs. Selected lineage-priming modules are shown. All gene sets are listed in Table S4g. **(E)** Volcano plot of differentially expressed surface proteins (p-value <0.05; log FC >0.5) in SAM0-med and SAM1-extramed HSC/MPPs from CITE-seq data of OD4. **(F)** Pseudotime of all transcriptionally defined HSC/MPPs in each tissue. Kruskal-Wallis test with multiple comparison. **(G)** GSEA of C2 curated MSigDB pathways (FDR <0.05 by pre-ranked GSEA) on differentially expressed genes between SAM0-med (n= 7,068 cells) and SAM1-extramed (n= 9,583 cells) HSC/MPPs. Selected gene sets are shown. All gene sets are listed in Table S4h. **(H)** scRNA-seq data from four mobilized PB (mPB) CD19^-^CD34^+^ HSPCs (28,026 cells) were integrated with the same BMs and non-mobilized PBs as in Figure 2. The cell density across the UMAP coordinates of each tissue is displayed as contours filled by a color gradient. Different HSPC groups are indicated by dashed lines. **(I,J)** Gene signatures of medullary and extramedullary type HSC/MPPs were used to compute a BM- or SPL-type identity score for each HSC/MPP cell of the multi-tissue landscape. Box plots show the ratio between BM- and SPL-type scores for each sample (‘identity ratio’). Notches indicate the 95% confidence interval of the median (middle line). **(I)** The ’identity ratio’ was calculated for BM and spleen HSC/MPPs taken from our 10x multi-tissue landscape, and then validated using transcriptionally defined HSC/MPPs from the HCA BM dataset^40^ (HCA-BM) as well as Smart-seq2 data from single-cell sorted phenotypic HSC/MPPs (CD19^-^CD34^+^CD38^-^CD45RA^-^) from BM (SS2 BM) and spleen (SS2 SPL) of OD1 and OD2. (J) Boxplots show the ’identity ratio’ for BM (10x BM), non-mobilized PB (10x PB) and mobilized PB (mPB) calculated using only the data integration containing these tissues. **(B-C)** Mean ± SD is shown. Two-tailed paired t-test. * p=0.02. SPL: spleen.

Given the distinct cellular microenvironments of BM and spleen, we hypothesized that HSC/MPPs in these anatomical locations would differ in their cellular composition and molecular properties. As we noted distinct abundances of transcriptionally defined HSC/MPP clusters 0 and 4 in BM and spleen (supplemental Figure 5A), we reclustered all HSC/MPPs from matching BM and spleen of the same individuals using the ‘Self Assembling Manifolds’ (SAM) algorithm^46^. Consistently across donors, unsupervised clustering yielded one cluster containing mainly BM cells (termed ‘medullary’, SAM0-med) and one cluster largely constituted of cells from spleen (‘termed ‘extramedullary’, SAM1-extramed, Figure 4A-B, supplemental Figure 5B, Table S2i). Within the HSC/MPP space, 90±7.5% of all spleen cells were of the extramedullary type, whereas 82±13% of BM cells were of the medullary type (Figure 4C).

Extramedullary HSC/MPPs differed from medullary HSC/MPPs by two main features. First, extramedullary HSC/MPPs were largely more lineage-primed than their BM counterparts. Gene signatures and surface markers of long-term HSCs^43,47^ were significantly enriched in medullary HSC/MPPs. In contrast, extramedullary HSC/MPPs had higher expression of gene sets associated with short-term HSCs and lineage committed progenitors^39,43,47^, the master regulator of quiescence exit *CDK6* and of surface proteins associated with HSC differentiation and activation (Figures 4D-E, supplemental Figure 5C, Tables S3e-i, 4f-g). Spleen HSC/MPPs were also significantly shifted downstream in the pseudotime trajectory of our multi-tissue reference map (Figure 4F) but were equally quiescent to BM HSCs by flow cytometry (Figure 3B-C). Second, extramedullary HSC/MPPs were marked by expression of gene sets and surface proteins linked to altered cytoskeleton organization, cell migration and adhesion (Figure 4E,G, supplemental Figure 5C, Tables S3e-i, S4h). Comparison of sorter-purified phenotypic HSC/MPPs from spleen and BM via Smart-seq2 (SS2) analysis confirmed the altered expression of cytoskeletal genes and transcriptional priming of splenic HSC/MPPs (supplemental Figure 5D, Tables S3j, S4i-j).

### A shared transcriptional HSC/MPP identity in spleen, PB and mPB

We next investigated the nature of HSC/MPP in non-mobilised PB and G-CSF mPB HSC/MPP. When comparing the 10x scRNA-seq HSPC landscapes from four mPBs (28,026 cells) to BM and steady-state PB, HSC/MPPs and early progenitors were expectedly most abundant in mPB (Figure 4H, supplemental Figure 5E-G, Table S2j-k). To assess if PB and mPB HSC/MPPs are globally more akin to BM or spleen HSC/MPPs, we derived a scoring method using the top differentially expressed genes between SAM0-med and SAM1-extramed HSC/MPPs (see methods, Table S3f-h). Ratios of these scores above 1 and below 1 respectively indicated medullary and extramedullary identities, including when benchmarked on independent datasets (SS2 BM and SPL HSC/MPPs and HCA dataset, Figure 4I).

Interestingly, both HSC/MPPs circulating in PB at steady-state and recently mobilized from the BM by G-CSF displayed a strong extramedullary identity (Figure 4J). Features of HSC/MPP medullary and extramedullary identity were also partially observed in early lineage progenitors, and to lower extent in late progenitors (Table S4k) and displayed complex patterns of expression dependent on anatomical location and G-CSF stimulation (Supplemental Figure 5H and Table S3k-l).

In summary, the vast majority of extramedullary HSC/MPPs (spleen, PB and mPB) share elements of a transcriptional identity distinct from that of BM HSC/MPPs, linked to lineage-priming, short-term repopulation capacity and different mechanical and adhesive properties of HSC/MPPs outside the BM microenvironment.

### Spleen HSPCs contribute to the erythropoietic response in chronic anemia

**Figure 5:**
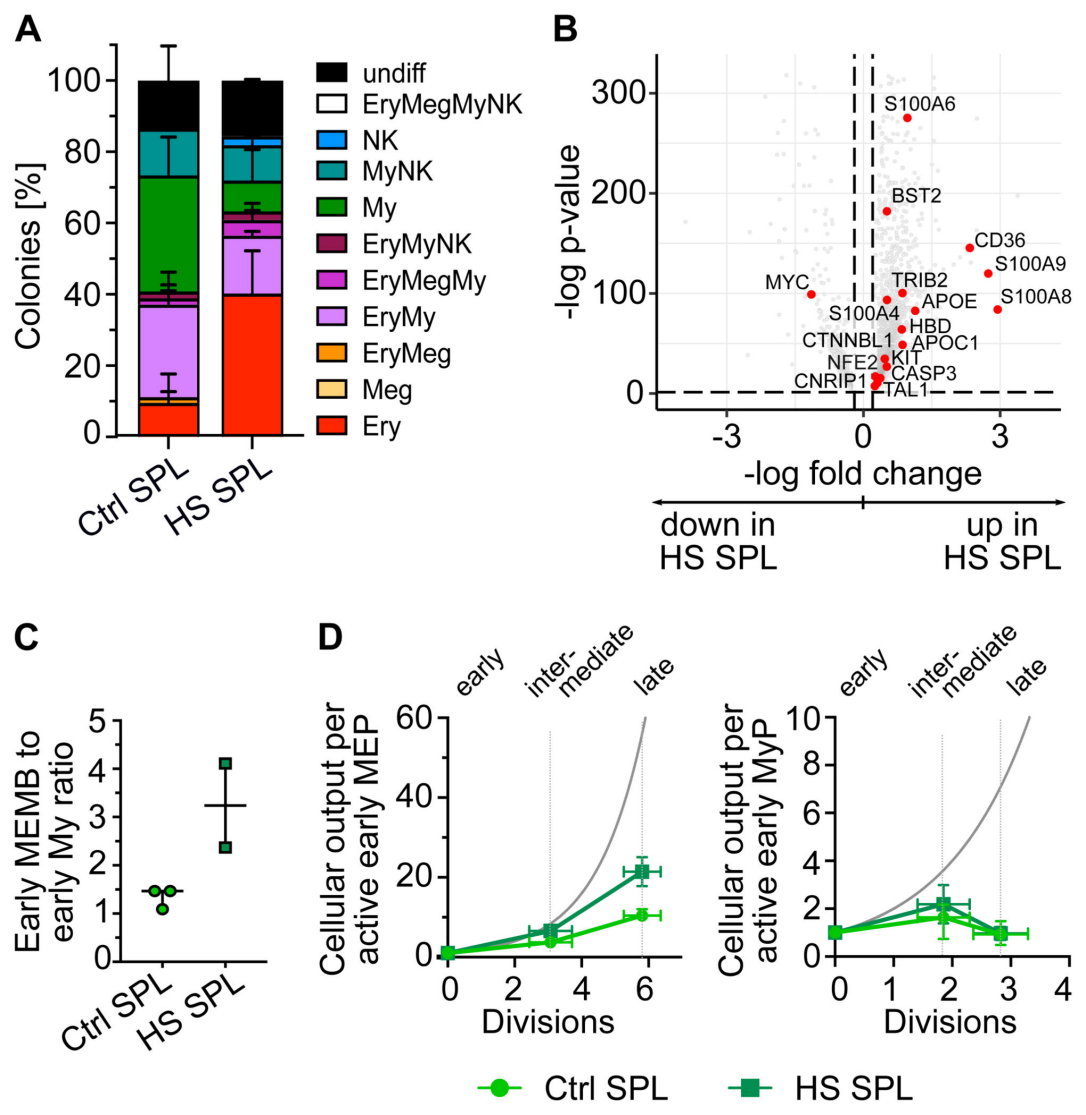
Spleen HSPCs in the anemia erythroid response. **(A)** Colonies derived from single phenotypic HSC/MPPs from control spleen (Ctrl, n=234 single cells from 3 donors) and spleens of hereditary spherocytosis (HS) patients (n=198 single cells from 2 donors) seeded into medium supporting My/Ly/Ery/Meg differentiation (see methods). Mean ± SD is shown. **(B)** CD19^-^CD34^+^ HSPCs (n=9,939 cells) from two HS patients were sequenced using the 10x Genomics scRNA-seq platform and were integrated with control spleen data (same as in Figure 2). UMAPs and cluster annotation of the HSPC landscape are shown in Supplemental Figure 6B-C. Volcano plot shows the differentially expressed genes (FDR<0.05, LFC>0.2) between transcriptionally defined HSC/MPPs from control and HS spleens. Genes associated with erythroid lineage commitment are shown in red. **(C)** Ratio of early MEMB to early My progenitors in control and HS spleens. Median ± 95% confidence interval is shown. **(D)** Normalized estimated cellular output from early to late progenitors of the MEMB (left) and the My branch (right) branch calculated as for Figure 3D-E (details see methods). Grey line: theoretical exponential expansion. Vertical error bars indicate the range observed in the different tissues. Horizontal error bars indicate the standard deviation of the estimated number of divisions for each expansion stage. Control spleens are same as in Figure 3D,E. SPL: spleen.

We next sought to assess whether extramedullary HSPC composition and function are modified under severe hematopoietic stress conditions. We analyzed splenic HSPCs from two hereditary spherocytosis (HS) patients displaying splenomegaly due to chronic anaemia (Table S1, Supplemental Figure 6A) by 10x scRNA-seq (9,939 cells, Supplemental Figure 6B, Table S2l-m) and single cell functional assays. Single phenotypic HSC/MPPs in HS spleens produced more Ery colonies *in vitro* than those in control spleens (Figure 5A) and displayed stronger transcriptional Ery-priming (Figure 5B, Table S3m). Furthermore, despite variability in progenitor composition between donors (Supplemental Figure 6C), splenic HSPCs in both HS spleens had a noticeably higher ratio of early Ery to My progenitors than control samples (Figure 5C). Importantly, whereas the lower bound expansion along the My branch did not change, differentiation along the Ery branch was estimated to be increased in HS spleens compared to controls, albeit not to the levels modeled in BM (Figure 5D). Altogether, these data indicate splenic HSPCs contribute to erythropoiesis in response to anemia in humans.

### Functional erythro-megakaryocytic skewing of HSC/MPPs is a distinctive feature of healthy PB

**Figure 6:**
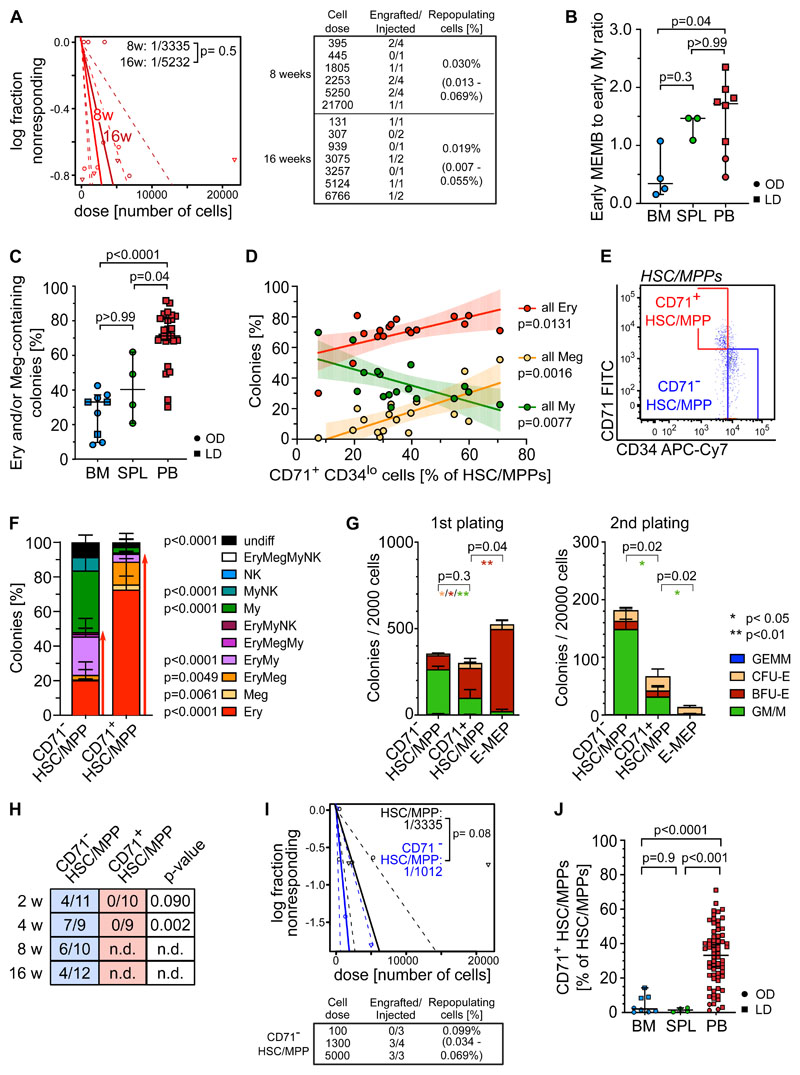
Multipotent repopulating HSC/MPPs and quiescent CD71^+^ HSC-like cells with restricted erythroid/megakaryocyte differentiation potential coexist in steady-state PB. **(A)** Frequency of repopulating cells in PB HSC/MPPs calculated using Extreme Limiting Dilution Analysis (ELDA^73^) statistics at 8 weeks (2 experiments, n=17) and 16 weeks (2 experiments, n=10) post-transplantation. Table indicates doses of cells injected and number of NSG mice with human cell engraftment in their BM (see methods). **(B)** Ratio of early MEMB (sum cluster 2,12) to early My (sum cluster 6,9) progenitors in all tissues. **(C-D)** Colonies derived from single phenotypic HSC/MPPs from BM (n=913 single cells from 7 samples), spleen (n=234 single cells from 3 samples) and PB (n=3,034 single cells; 27 independent PBs over 16 experiments) seeded into medium supporting My/Ly/Ery/Meg differentiation (see methods). **(C)** Frequency of colonies containing Ery and/or Meg cells for each tissue as assessed by flow cytometry. **(D)** Relationship between the percentage of all Ery-, Meg- and My-containing colonies and the proportion of CD71^+^CD34^lo^ cells within the phenotypic PB HSC/MPP pool. Linear regression and 95% confidence interval are indicated by solid line and shaded area. n=17 PBs. **(E)** Representative pseudocolor plot for flow cytometry isolation of CD71^-^ and CD71^+^ HSC/MPPs in PB gated on phenotypic HSC/MPPs (CD19^-^CD34^+^CD38^-^CD45RA^-^ cells as defined in Supplemental Figure 1D). **(F)** Percentage of colonies generated by CD71^-^ (n=872 single cells; 15 independent PBs) and CD71^+^ (n=1,109 single cells; 18 independent PBs) HSC/MPPs. p-values comparing CD71^-^ and CD71^+^ HSC/MPP colony output are shown. Two-tailed Mann-Whitney test. **(G)** Serial replating of PB CD71^-^ or CD71^+^ HSC/MPPs and CD71^+^ MEPs (E-MEPs) in methylcellulose medium. Colony numbers per indicated number of seeded cells after first (left) and secondary (right) plating are shown. n=4 PBs over 3 experiments. Paired two-tailed t-test. ** p<0.01; * p<0.05; ns = not significant, p>0.05. **(H)** Ratio of NSG mice engrafted to total mice tested at the indicated time points after transplantation of CD71^-^ and CD71^+^ phenotypic PB HSC/MPPs. n.d.: not determined. p-values comparing engraftment of CD71^-^ and CD71^+^ HSC/MPPs were determined by two-tailed Fisher-test and are shown below each time point. **(I)** Frequency of repopulating cells within all phenotypic PB HSC/MPPs (same as Figure 5A) and CD71^-^ HSC/MPPs at 8 weeks after transplantation using ELDA statistics. **(J)** Percentage of CD71^+^ cells withing the phenotypic HSC/MPP pool of BM (n=8), spleen (n=4) and PB (n=65). One-way ANOVA; Tukey’s multiple comparison. **B-C)** Median ± 95% confidence interval is shown. Kruskal-Wallis; Dunn’s multiple comparison test. F,G,J) Mean ± SD is shown. LD: living donor, OD: organ donor, SPL: spleen.

Due to their rarity, little is known about the function of purified HSC/MPPs that circulate in healthy individuals’ blood. HSC/MPPs with long-term repopulating capacity were detected 8 and 16 weeks post-transplantation (Figure 6A, supplemental Figure 7A, Table S5), but were very rare, around 60 and 3 times lower than that reported for similar phenotypic populations in cord blood^48^ and mPB^49^ respectively. This low repopulation frequency of PB HSCs is consistent with the overall transcriptional features of extramedullary HSC/MPPs.

Not only are early progenitors markedly overrepresented in PB HSPCs compared to BM (Figure 2D), but, unlike in BM, early MEMB progenitors vastly dominate over early My progenitors (Figure 6B, supplemental Figure 7B). We thus tested whether this overrepresentation of non-proliferative early MEMB progenitors is associated with lineage-skewing in phenotypic PB HSC/MPPs. For this, we index-sorted 4,181 single HSC/MPPs from BM, spleen and PB and cultured them in medium promoting simultaneous differentiation into My, Ery, Meg and Ly (NK) lineages^35^. Phenotypic HSC/MPPs from all tissues gave rise to a range of uni- or oligo-lineage colonies (supplemental Figure 7C-D). Notably, >70% of PB HSC/MPP-derived colonies contained Ery and/or Meg cells, with a particularly high proportion of unilineage Ery colonies, significantly more than in BM and spleen (Figure 6C, supplemental Figure 7D).

Analysis of index sorting data highlighted a strong positive correlation between the proportions of Ery-containing colonies and the percentage of PB HSC/MPPs expressing high levels of CD71 and low levels of CD34 (Figure 6D, supplemental Figure 7E). Prospective isolation of CD71^+^CD34^lo^ cells (hereafter termed CD71^+^ HSC/MPPs) from the PB HSC/MPP pool (Figure 6E) confirmed that they almost exclusively differentiated towards the Ery/Meg lineage (Figure 6F, supplemental Figure 8A-C), whereas CD71^-^CD34^hi^ cells (CD71^-^ HSC/MPPs) had a balanced lineage output. CD71^+^ HSC/MPPs clustered in a specific area of extramedullary HSC/MPP cluster 0 in the multi-tissue landscape (supplemental Figure 8D) and displayed several features distinguishing them from both CD71^-^ HSC/MPPs and classically defined Ery/Meg progenitors, such as CMPs and MEPs. Despite being as quiescent (supplemental Figure 8E), CD71^+^ HSC/MPPs expressed significantly higher levels of Ery/Meg-associated gene sets than CD71^-^ HSC/MPPs (supplemental Figure 8F, Table S3n, S4l-m). CD71^+^ HSC/MPPs were delayed in their first division *in vitro* compared to MEPs (supplemental Figure 8G) and expressed signaling and mitochondrial proteins to levels similar to those of CD71^-^ HSC/MPPs but significantly lower than CMP/MEPs (supplemental Figure 8H). CD71^+^ HSC/MPPs also produced more bi-potent Ery-Meg colonies than MEPs (supplemental Figure 8I), and their serial replating was intermediate between that of CD71^-^ HSC/MPPs and MEPs (Figure 6G). Only CD71^-^ HSC/MPPs expressed high levels of CD90 on their cell surface and gave rise to myelo-lymphoid reconstitution in NSG mice, whereas CD71^+^ HSC/MPPs were largely CD90^-^ and failed to engraft even in the short-term (Figure 6H-I, supplemental Figure 8J-K, Table S5).

Our data show that, whereas very rare bona fide HSCs do circulate in blood (a small proportion of CD71^-^ HSC/MPPs), most phenotypic PB HSC/MPPs are quiescent, lowly active cells committed to the Ery/Meg lineage, with broad molecular similarity to HSCs, but no self-renewal capacity *in vivo* (CD71^+^ HSC/MPPs). Strikingly CD71^+^ HSC/MPPs predominate in non-mobilized PB, whereas they are rare in BM and spleen (Figure 6J, supplemental Figure 8L). Altogether, we demonstrate that in contrast to BM and spleen, PB HSPC composition is uniquely skewed towards Ery/Meg differentiation both at the HSC/MPP and the progenitor level.

### The Ery-Meg skewing of healthy PB HSC/MPPs is conserved with age but suppressed in disease

**Figure 7:**
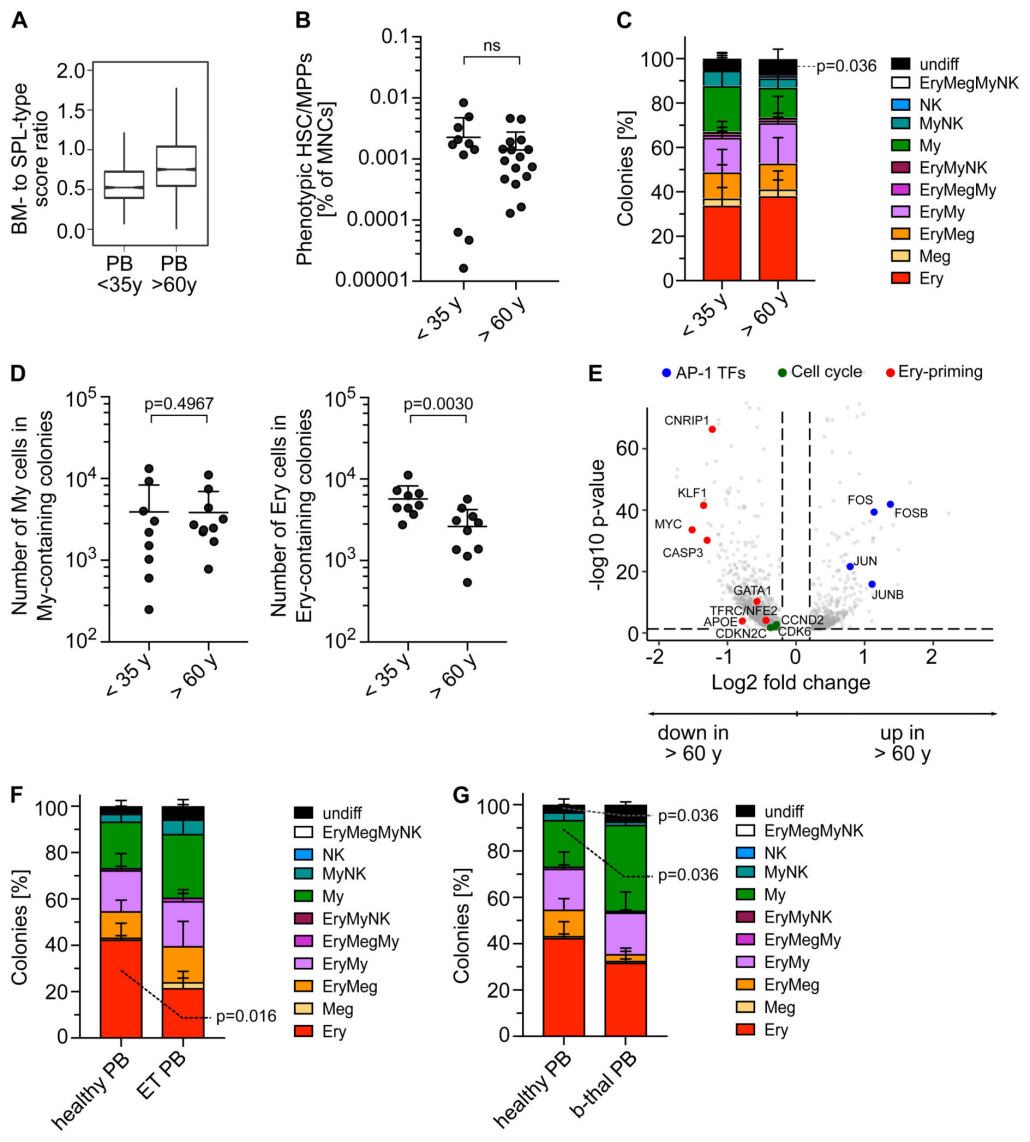
PBs unique erythroid/megakaryocyte-biased differentiation output becomes imbalanced with age and disease. **(A)** Ratio between BM- and SPL-type identity scores in PB HSC/MPPs by age group. Notches indicate the 95% confidence interval of the median (middle line). **(B)** Frequencies of phenotypic HSC/MPPs (CD19^-^CD34^+^CD38^-^CD45RA^-^) in PB mononuclear cells (MNCs) by age group (n=27, same data as in Supplemental Figure 1B). **(C)** Percentage of colonies generated by single cell sorted phenotypic HSC/MPPs from non-mobilized PB (same as in Figure 6C), grouped by age. <35 years; n=942 single cells; 9 independent PBs; >60 years; n=700 single cells; 8 independent PBs. **(D)** Median size of all myeloid colonies (left) and all erythroid colonies (right) generated from non-mobilized PB HSC/MPPs, grouped by age as in Figure 7C. **(E)** Volcano plot showing selected genes differentially expressed genes (FDR <0.05) in EryP (cluster 8) of PB donors <35 years and >60 years. **(F)** Percentage of colonies generated by single cell sorted phenotypic HSC/MPPs from non-mobilized PB of healthy individuals (n=445 cells, 5 healthy controls) and ET patients (n=349 cells, 5 individuals) over 5 experiments. **(F)** Percentage of colonies generated by single cell sorted phenotypic HSC/MPPs from non-mobilized PB of healthy individuals (same as in Figure 7F) and b-thalassemia patients (n=271 cells, 3 individuals) over 3 experiments. **(B,C,D,F,G)** Mean± SD is shown. Two-tailed Mann-Whitney test.

Given that ageing and disease lead to BM HSC lineage skewing^50,51^, we next checked whether the molecular, phenotypic and functional composition of the PB HSC/MPP pool was perturbed with age. Transcriptionally defined HSC/MPPs of older PB donors had markedly higher BM- to SPL-type score ratios than younger donors (<35 years, Figure 7A). Age had no significant effect on i) frequencies of phenotypic HSC/MPPs (Figure 7B), ii) the abundance or function of CD71^+^ HSC/MPPs, and iii) colony formation efficiency or differentiation balance within the entire PB HSC/MPPs pool (Figure 7C, supplemental Figure 9A-C). However, PB HSC/MPPs from older individuals produced significantly smaller erythroid colonies than HSC/MPPs from young donors (Figure 7D), indicating decreased proliferation potential with age. Accordingly, compared to younger donors, PB Ery progenitors from older donors expressed significantly lower levels of Ery lineage master regulators (GATA-1, KLF and MYC) and genes of the CDK4-6/Cyclin D complex, key for cell cycle progression as well as Ery differentiation^52^ (Figure 7E, Table S3o-p).

Next, we assessed whether the unique lineage-skewing of PB is affected by disease. Given recent reports of Meg bias in myelofibrosis and essential thrombocythemia (ET) BM HSCs^53–55^, we measured the phenotypic (Supplemental Figure 9D) and functional composition of circulating PB HSC/MPPs from ET patients. ET PB HSC/MPPs showed a shifted differentiation balance, with significantly decreased Ery colonies compared to healthy PB (Figure 6F), independently of the JAK2 mutational status of individual cells within the sample (Supplemental Figure 9E). Interestingly, increased My production at the expense of the Ery lineage was also observed in PB HSC/MPPs of anemic patients with b-thalassemia (Figure 6G, supplemental Figure 9F). These data suggest that disease-driven microenvironmental changes and/or BM hematopoietic dysfunction lead to a shift in the differentiation balance of PB HSC/MPPs across a range of diseases, opening the way for new clinical diagnostic strategies.

## Discussion

Here we report largely distinct HSPC compositions in human BM and extramedullary sites. Unlike BM, PB and spleen are not sites of active hematopoiesis at steady state. Instead, they harbor poised cellular reservoirs centered around quiescent but lineage-primed HSC/MPPs and early progenitors with suppressed proliferation. Extramedullary HSPCs remain capable of differentiative expansion both *in vitro* and *in vivo*, as shown here in the context of chronic anemia induced stress erythropoiesis. Our data provide proof-of-principle evidence of participation, albeit likely limited, of spleen HSPCs to human demand-adapted hematopoiesis.

Major challenges to the functional characterization of human extramedullary HSPCs are their inherent rarity and sampling within ethical guidelines (i.e. requirement for large PB volumes and invasiveness of spleen sampling). Here we have overcome some of these limitations by using single cell methods and by leveraging tissue collection from organ donors and healthy living donors wherever ethically possible (BM and PB). We observed slight changes between organ donor and living donor derived hematopoietic tissues (Supplementary Note 1), likely originating from traumatic death circumstances leading to pro-inflammatory cytokine^56,57^ and catecholamine release^58,59^. We therefore cannot exclude that some molecular features observed here in organ donor spleen are influenced by death-induced cellular responses. Importantly though, all key features of extramedullary HSPCs were observed independently of sample source and with effect sizes well beyond those observed for differences between organ and living donors. Hence, we infer that collectively, our data highlight general inter-tissue differences of physiological relevance.

We provide evidence that rare but transplantable HSC/MPPs exist in human PB, only so far postulated from mouse models^5^, and define a transcriptional identity for PB, mPB and spleen HSC/MPPs, indicative of their biology. Collectively our data indicate that most extramedullary HSC/MPPs are of the short-term HSC, lineage-primed type. They also differ from their BM counterparts in genes and cell surface proteins linked to activation, cell adhesion and cytoskeleton reorganization. The latter may confer increased motility^60–63^ and loss in cell polarity and HSC function, similar to that of aged mouse HSCs^64^, and/or lead to changes in extramedullary HSC/MPPs lineage-priming^65^. Many of these features are also observed in mPB HSCs, suggesting they are acquired rapidly upon mobilization, hence likely due to either the loss of interaction with the BM niche^10^ or the effects of G-CSF itself. Future work will have to develop new tools to study how the dynamics of HSC/MPP tissue residency links to the acquisition of specific extramedullary features.

We also report a unique functional bias of PB HSPCs towards erythropoiesis. This is initiated at the HSC/MPP level and driven by Ery/Meg-biased CD71^+^ HSC-like cells almost exclusively found in PB. CD71 is an early marker for HSC activation^66^ and Ery differentiation, used to enrich human Ery and/or Meg-primed CMP/MEP populations from numerous tissues across development^36,43,48,67^. However, in adult human BM and spleen less than 15% of phenotypic HSC/MPPs contain CD71^+^ cells. Predominance of CD71^+^ HSC/MPPs in homeostatic PB may thus reflect either enhanced BM egress relative to other HSC/MPP subsets, or functional biases induced by a changing environment, including adaptation to different iron levels^68,69^. Collectively we identify a circulating reservoir of adult MPPs/progenitors, likely seeding spleen, skin and/or lung where erythropoiesis and megakaryopoiesis have respectively been observed under stress conditions^3,70^.

Non-mobilized PB is by far the easiest to sample of all hematopoietic tissues. Hence it is ideally suited for early diagnosis, prevention and patient management in disease, hematological or not, bypassing the needs for invasive tissue biopsies. To date the clinical potential for PB HSPC screening remains untapped, largely due to lack of understanding of circulating HSPC biology. Here we have established a healthy HSPC baseline in humans and shown phenotypic and functional imbalances in circulating HSC/MPPs from the elderly, in ET and b-thalassemia patients. Recently we also reported My-skewing in PB progenitors from chronic lymphocytic leukemia patients^71^. With single cell technologies becoming increasingly embedded into clinical protocols, our data warrants further investigation of PB HSPC subsets in large cohorts of patients at high resolution, with the purpose of identifying novel diagnostic or monitoring biomarkers.

## Supplementary Material

Supplementary Material

Table S1

Table S2

Table S3

Table S4

## Data Availability

Sequencing files and metadata associated to 10x Genomics scRNA-seq are deposited at the European Nucleotide Archive, accessible via BioStudies (identifiers SUBS4 and SUBS10): https://www.ebi.ac.uk/biostudies/studies/S-SUBS4 https://www.ebi.ac.uk/biostudies/studies/S-SUBS10 or via GEO (accession number GSE190067). The data can also be explored by the interactive Web portals: http://bioinf.stemcells.cam.ac.uk:3838/laurenti/ExtramedHSPCs/ and http://bioinf.stemcells.cam.ac.uk:3838/laurenti/mobPB_HSPCs/ Data from Smart-seq2 datasets are available at GEO with accession numbers GSE143567 (BM/spleen phenotypic HSC/MPPs) and GSE131409 and associated superseries_(CD71^-^/CD71^+^ HSC/MPPs). All code is publicly available at https://github.com/elisa-laurenti/ExtramedHSPC.

## Key Points

- Extramedullary tissues harbor reservoirs of HSC/MPPs and non-proliferative progenitors without ongoing hematopoiesis.
- Functional bias of peripheral blood HSPCs towards erythropoiesis, mediated by CD71^+^ HSC/MPPs and suppressed with age and disease.
