# Supplementary material for: Unique molecular and functional features of extramedullary hematopoietic stem and progenitor cell reservoirs in humans
Source: Blood. Author manuscript; Available in PMC 2022 Jun 11. (PMC7612845; doi:10.1182/blood.2021013450)
Supplement: Supplementary Material [file EMS145429-supplement-Supplementary_Material.pdf]

Supplemental data for Mende et al.

- Supplemental methods
- Supplemental note 1
- Supplemental tables 1-9
- Supplemental figures 1-9
- References for supplemental data

SUPPLEMENTAL METHODS

Sample preparation

For isolation of mononuclear cells (MNCs) from BM and PB taken by venipuncture (ET, b-thalassemia patients, and healthy controls), samples were diluted 1:1 in PBS prior to density gradient centrifugation on Pancoll (PAN-Biotech) or on LymphoPrep (Axis-Shield). The MNC fraction was collected, and red blood cells were lysed in RBC lysis buffer (BioLegend) or Ammonium Chloride Solution (Stem cell Technologies) for 15 min at 4 °C. PB from leukoreduction filters was lysed twice by incubation with RBC lysis buffer for 5 min at 4 °C, followed by density gradient centrifugation and MNC harvest. Freshly collected spleen samples (OD spleen and HS spleen) were immediately placed into 4˚C UW solution and kept up to 12 hours at 4˚C until processing. Cell suspensions were obtained by placing small sections of spleen (<5 mm^3) with cold phosphate buffer into a benchtop gentleMACS™ dissociator (Miltenyi Biotec). Then, MNCs were enriched through density gradient separation followed by red cell lysis. For some samples, $CD34^+$ selection was performed prior to freezing using the CD34 MicroBead Kit (Miltenyi Biotec) and manual separation on LS columns. MNC and $CD34^+$ cell suspensions were frozen in FBS/10% DMSO and stored at -150 °C until use. Prior to flow cytometry, frozen MNCs or $CD34^+$ enriched cells were thawed by drop wise addition of pre-warmed IMDM (Life Technologies) + 0.1 mg/ml DNase (Worthington) + 50% Fetal Bovine Serum (FBS, Life Technologies). If required, magnetic $CD34^+$ selection from thawed MNCs was performed as described above.

Flow cytometry

All antibodies are listed in Table S6 and detailed antibody panels can be found below. Cells were stained for 20 min at room temperature (RT) in the dark using antibodies diluted in PBS + 3% FBS (PBS/FCS), followed by two washes in PBS/FCS. For cell cycle analysis, cell surface marker stained samples were fixed in Cytofix/Cytoperm, followed by two washing steps in Perm/Wash according to the manufacturer's protocol (BD). Intranuclear staining was performed at 4 °C overnight in the dark using the FITC anti–human Ki-67 antibody (BD) diluted in Perm/Wash. The next day, DNA content was stained by 20 min incubation in 0.5 µg/ml DAPI (BioLegend) in Perm/Wash at RT, followed by one wash with Perm/Wash. Prior to flow cytometry, all samples were resuspended in PBS/FBS and filtered through a 20 µm cell strainer.

For CITE-seq[1] of OD3 (BM), OD4 (BM and spleen), mPBs and HS spleen, $CD34^+$ cells were resuspended at a concentration of $1x10^6$ cells / 50 µl PBS/FBS and blocked with

5 µl Human TruStain FcX™ Fc Blocking Reagent (BioLegend) for 10 min at 4 °C. In parallel, customized lyophilized TotalSeq™-A antibodies (BioLegend, Table S7) were reconstituted in 50 µl PBS/FBS following the manufacturer's protocol. Reconstituted TotalSeq™-A antibodies were added at a concentration of 50 µl antibody mix / $1x10^6$ cells and incubated for 30 min at 4 °C. Simultaneously, cells were stained with HSPC antibody panel (E). After three washes with PBS/FBS, cells were resuspended in PBS/FBS and filtered for flow cytometry.

Samples were sorted or analyzed at the NIHR Cambridge BRC Cell Phenotyping Hub using the BD FACSAria Fusion and FACSAria III sorters or BD LSRII and LSRFortessa cytometer, respectively. Single-cell sorts were performed using single-cell purity and index-sorting modes, while purity mode was chosen for bulk sorts. The average sort purity was >95%. High-throughput analysis was performed using the BD High Throughput Sampler (HTS). Data were analyzed using FlowJo software v9.9 or v10 (Tree Star).

Antibody panels

Clone, supplier, and catalogue number for each antibody can be found in Table S6. Antibody panels A-F were used to sort and phenotype subsets of hematopoietic stem and progenitor cells. Panel (A): CD19/BV785, CD34/APC-Cy7, CD38/PE-Cy7, CD90/APC, CD45RA/A700, CD49f/PE-C5, CD10/BV421, CD71/FITC, CD117/BV650, CLEC9A/PE, Zombie Aqua; Panel (B): CD19/BV785, CD34/APC-Cy7, CD38/PE-Cy7, CD90/APC, CD45RA/A700, CD49f/PE-C5, CD41a/BV510, CD71/FITC, CD117/BV650, CLEC9A/PE, Zombie UV; Panel (C): CD45RA/FITC, CD90/PE or CD90/APC, CD49f/PE-Cy5, CD38/PE-Cy7, CD10/APC or CD10/BV421, CD19/A700, CD34/APC-Cy7, CD7/BV421, Zombie Aqua; Panel (D): CD19/BV785, CD34/APC-Cy7, CD38/PE-Cy7, CD36/APC, CD45RA/A700, CD71/PerCP-Cy5.5, GlyA/BV421, CD90/FITC, CD45/BV605, CD135/PE, Zombie Aqua. Panel (E): CD71/FITC, CD90/PE, CD38/PE-Cy7, CD45RA/A700, CD34/APC-Cy7, CD49f/PE-Cy5, CD41a/BV510 or Zombie Aqua, CD19/BV785 or CD123/BV785, CD105/BV650, Zombie UV. In some cases, a CD36/APC, CD41/APC or CD135/A647 antibody was added to the staining in panel (E). In other cases, CD90/PE was replaced by CD90/APC, CD45RA/A700 was replaced by CD45RA/BV421 and a CD135/PE or CD71/PE or CD71/BV650 antibody was added. Panel (F): CD3/FITC, CD5/PE, CD49f/PE-Cy5, CD34/APC-Cy7, CD38/PE-Cy7, CD90/APC, CD45RA/A700, Streptavidin/BV510 (BioLegend, dilution 1:400), Zombie UV. After exclusion of dead cells based on Zombie dyes (BioLegend), HSPCs populations were defined as previously described: total HSPC pool ($CD19^-CD34^+$), phenotypic HSC/MPPs

(CD19^{-}CD34^{+}CD38^{-}CD45RA^{-}), CMP/MEPs (CD19^{-}CD34^{+}CD38^{+}CD45RA^{-} CD10/CD7^{-}), MEPs (CD19^{-}CD34^{+}CD38^{+}CD45RA^{-}CD123^{-}), E-MEPs (CD19^{-}CD34^{+}CD38^{+}CD45RA^{-}CD123^{-}CD71^{+}).

For cell cycle analysis, panel (G) or (H) were used. Panel (G): CD45RA/PE, CD90/APC, CD49f/PE-Cy5, CD38/PE-Cy7, CD10/BV421, CD19/A700, CD34/APC-Cy7, CD7/BV421, KI-67/FITC, DAPI. Panel (H): CD19/APC-Fire750; CD34/APC, CD38/PE-Cy7, CD45RA/PE, CD71 /PerCP-Cy5.5, CD123/BV785, KI-67/FITC, DAPI.

Colonies from My, Ly, Ery and Meg single-cell differentiation assays were stained with Panel (I): CD45/PE-Cy5, CD41/FITC, CD11b/APC-Cy7, CD14/PE-Cy7, CD15/BV421, GlyA/PE, CD56/APC or (J): CD45/PE-Cy5, CD71/FITC, CD11b/APC-Cy7, CD14/PE-Cy7, CD15/BV421, GlyA/PE, CD56/APC, CD41/BV510. Colonies from single-cell Ery/Meg differentiation assays were stained with panel (K) CD71/FITC, GlyA/PE, CD45/PE-Cy5, CD14/PE-Cy7, CD42b/APC, CD11b/APC-Cy7, CD15/BV421, CD41a/BV510.

At the time point of final analysis, mouse bone marrow was stained with antibody panel (L): GlyA/PE, CD19/FITC, CD45/PE-Cy5, CD14/PE-Cy7, CD33/APC, CD19/A700, CD45/BV510, CD3/APC-Cy7; or panel (M): GlyA/PE-Cy7, CD19/FITC, CD71/PE, CD45/PE-Cy5, CD14/APC-Cy7, CD33/APC, CD19/A700, CD45/BV510.

Index-sorting analysis

Principal component analysis (PCA) of single-cell indexed surface marker expression, recorded as .csv file during FACS sorting, was performed with R (version 3.4.0 or above) as previously described [2]. In brief, data collected from different sorts occurring on different days was normalized using the pipeline established in [3]. Therefore, data from individual experiments were logicle transformed using the estimateLogicle function (m = 4.5) of the FlowCore package and were then normalized with the ComBat function from the sva package. Next, principal component analysis on the normalized data was performed using the prcomp function. The prcomp rotation variable was used to obtain vector loadings.

Mass cytometry

Three independent LD-PB donors were used for mass cytometry experiments. Staining for heavy metal labeled antibodies (Table S8, purchased from Fluidigm or labeled using Maxpar antibody labeling kit) was performed using the manufacturer’s reagents and protocol (Fluidigm). Briefly, cells were washed once with PBS and stained with 1 µM Cell-ID Cisplatin for 15 min at RT to exclude dead cells. Cells were

fixed with MaxPar Fix I buffer and stained with biotinylated antibodies labeling lineage positive cells (Lin: CD2, CD3, CD4, CD7, CD8, CD10, CD11b, CD14, CD19, CD20, CD56, CD235a) in 100 µl of cell staining buffer for 30 min at RT. Cells were washed with MaxPar cell stain buffer and stained with heavy-metal labeled surface marker antibodies, including a heavy-metal labeled anti-biotin secondary antibody, in 100 µl of cell staining buffer for 1 h at 4°C. Cells were washed twice and permeabilized with 1 mL of methanol for 25 min at 4°C. Cells were then stained with heavy-metal labeled cytoplasmic and nuclear antibodies in 100 µl of cell staining buffer for overnight at 4°C, washed and resuspended in 1ml of intercalation solution containing Cell-ID Intercalator-Ir for 1hr at RT, washed and resuspended in Maxpar water for data acquisition on Helios (Fludigm). After gating of the indicated populations in the Cytobank software (Fluidigm), raw data of signal intensity was exported and data was processed in R (v3.4.0). First, the median of signal intensities in each channel was computed for each population. Next, heatmaps were generated using the gplots package heatmap.2 function, whereby data was scaled based on channel intensities.

Library preparation using the Smart-seq2 protocol

The Smart-seq2 protocol was used for sequencing of i) single phenotypic HSC/MPPs from OD BM, spleen and PB, ii) single CD71^{+} HSC/MPPs from LD-PB and iii) for minibulk (20 cells) sequencing of CD71^{-} and CD71^{+} HSC/MPPs from LD-PB. Library preparation was adapted from the Smart-seq2 protocol of [4]. Individual cells (or 20 cells for minibulk sequencing) were directly sorted into 4 µl of lysis buffer (0.22% (v/v) Triton X-100, 11.5 U/µl RNase inhibitor (Clontech), 5 mM DTT, 1 mM dNTP in nuclease-free water (Ambion, Life Technologies)) per well of 96-well PCR plates. Plates were spun down immediately after sort and stored at −80 °C until library preparation. Plates were thawed on ice and 10 µM OligodTs (Sigma) and ERCCs at a final concentration of 1:30,000,000 (Ambion, Life Technologies) were added, followed by annealing for 3 min at 72 °C. Next, reverse transcription was carried out using 50 U/well SmartScribe (SmS) RT enzyme (Takara) + 1x SmS First Strand buffer (Takara) + 5 µM TSO (Qiagen). Amplification of cDNA (22 amplification cycles) was performed using 0.55 µM ISPCR primers (Sigma) and 1 x KAPA HiFi Hotstart mix (Roche). After purification of the PCR product with a 1: 0.6 ratio of PCR product to AMPure XP beads (Beckman Coulter), the success of cDNA preparation was confirmed using a 2100 Bioanalyzer with High-sensitivity DNA chip (Agilent). Illumina library preparation with the Nextera XT DNA Library Prep Kit (Illumina) was carried out following manufacturer’s instructions. Size distribution of the libraries was analyzed on a High-sensitivity DNA chip (Agilent) and the concentration of indexed libraries was quantified following the

protocol of the KAPA library quantification kit (Kapa Biosystems). For each organ donor (OD), the libraries from all tissues (containing 380 - 464 cells) were combined prior to sequencing and sequenced on the Illumina HiSeq 4000 system in 2 lanes. For OD1 only BM and spleen phenotypic HSC/MPPs were sequenced using this method, for OD2 phenotypic HSC/MPPs from all three tissues were sequenced using Smart-seq2 (see Table S9). The library from 192 single $CD71^{+}$ LD-PB HSC/MPPs was sequenced on 2 lanes of the Illumina HiSeq 4000 system. For minibulk sequencing of $CD71^{-}$ and $CD71^{+}$ HSC/MPPs from LD-PBs all samples (32 pools of 20 cells) were combined prior sequencing on 2 lanes of the Illumina HiSeq 4000 system.

Smart-seq2 analysis

Resulting reads from each OD were independently aligned and mapped against Ensembl genes (release 81) [5] using GSNAP (version 2015-09-29) [6] and quantified using HTSeq (version 0.6.0) [7]. Quality control filters were applied rejecting cells with less than 50,000 (OD1) or 200,000 (OD2) reads mapping to nuclear genes which in turn had to be at least 10% of the mapping reads in the cell. Additionally, the maximum allowed fraction of cells mapping to mitochondrial genes was set at 20%. The levels of technical variance were estimated using the ERCC spike-ins as described by [8] with highly variable genes (HVGs) being defined as having the squared coefficient of variation exceeding technical noise. Prior to differential gene expression analysis, the Smart-seq2 data filtered count matrices from both donors were combined and HVGs again were determined as described for the individual donors above. Principal component analysis (PCA) was calculated on the HVGs and the cells falling outside the upper and lower limits of the boxplot for the three first principal components were removed as outliers (Table S9). Differential expression tests were performed using the R packages DESeq2 [9] with donor identity preserved as a covariate in the design matrix. The batch correction method Combat, was then applied as described in [10] after which the HVG set was recomputed, using the method described in [11]. This recomputed HVG set was then subsequently used to recompute PCA and the first two components plotted.

Reads obtained from the single $CD71^{+}$ HSC/MPP cells were processed and filtered through the same procedure described above up to resulting in a counts table that was later further processed to project against a HSC/MPP cells reference (described in methods below).

Analysis of the minibulk data from $CD71^{-}$ and $CD71^{+}$ HSC/MPPs was performed as follows. After alignment to the human reference using GSNAP and quantification with HTseq, quality control (QC) was performed for the resulting read counts using the

bglab package ([https://github.com/wjawaid/bglab]) on R v3.4.0 (or higher). All samples passed quality control and had >$2x10^5$ reads mapped to a gene feature. Technical replicates clustered together by PCA. Prior to further analysis, read counts of technical triplicates were summed and treated as one sample for all following steps. Data were normalized for sequencing depth using size factor calculated on ERCCs spike-in genes [12]. Highly variable genes (HVGs) were selected with the techVar function of the bglab package, setting the MeanForFit parameter at 10 [8]. PCA was performed on the 184 detected HVGs using the runPCA function (bglab package). To determine differential gene expression between $CD71^-$ and $CD71^+$ HSC/MPPs with DESeq2, the doDESeq wrapper function of the bglab package was used [9].

Library preparation using the 10x Genomics platform

Cells resuspended in 47 µl PBS + 0.04% BSA were immediately processed for single-cell sequencing using the Chromium™ Single Cell 3’ Library & Gel Bead Kit v2 (OD1, OD2) and v3 (OD3, OD4, all living donor PBs - LD-PBs 1-6, G-CSF mobilized PB – mPB 1-4, Hereditary Spherocytosis spleens – HS1-2) (10x Genomics) following the manufacturer’s protocols. RNA and cell surface protein libraries were generated following the manufacturer’s protocols (10x Genomics and BioLegend, respectively). For some samples, pools of cells from multiple donors (mPB1+mPB2; mPB3+mPB4; HS1+HS2) were combined and processed together during library prep. Following sequencing, donors were demultiplexed using SNP information from the resulting data with the soupourcell[13] software pipeline.

For each OD, the libraries from all three tissues were combined prior to sequencing and sequenced on the Illumina HiSeq 4000 system in 2 (OD1) or 3 lanes (OD2). For all LD-PB samples, the libraries from 2 or 3 donors were combined and sequenced in 1-2 lane on the Illumina NovaSeq 6000 system. For CITE-seq of OD3 and OD4, as well as the mPB and HS spleen samples, libraries were sequenced on the Illumina NovaSeq 6000 system, aiming for a minimum of 20,000 reads per cell for gene expression and 5,000 reads per cell for cell surface protein.

The resulting scRNA-seq reads were aligned to the GRCh37 (hg19) reference genome and quantified using cellranger count (CellRanger pipeline versions 2.1.1 [OD1-2]; 3.1.0 [OD3-4, LD1-6]; or version 6.0.1 [mPB1-4, HS1-2]), giving a cell recovery of 31-60% for each donor and tissue (Table S9 for the number of cells passing quality control). Multiplets were estimated using the scrublet Python package[14] and subsequently removed.

10x scRNA-seq data - Filtering

For all donors, further gene and cell filtering was applied to the 10x scRNA-seq datasets, either independently or as combined datasets. Genes being expressed in fewer than 3 cells and cells expressing fewer than 500 genes or cells with 10% (mPB3, mPB4, HS1, HS2), 15% (mPB1, mPB2) or 20% (OD1-4, LD PB 1-6, unless otherwise stated) or more of its expressing genes being mitochondrial were excluded. Thresholds were chosen based on visual inspections of feature distributions. These filtering steps were applied resorting to Scanpy Python package[15] inbuilt filtering methods. The subsequent analyses pipeline is summarized in supplemental Figure 10.

10x scRNA-seq data – Normalization

The filtered count matrices from OD-BM, OD-spleen, OD-PB and LD-PB data were combined into an anndata object using the Scanpy[15] Python package. This combined dataset was then log-normalized and its HVGs determined using the inbuilt package methods, into what is hence referred here as the *multitissue base normalized dataset*. The filtered counts matrices from the (four) mPB and (two) HS samples were also combined into two respective datasets as described in the next section and processed, as above, into log-normalized datasets (supplemental Figure 10).

10x scRNA-seq data - Seurat3 integration

Three different data integrations were performed using the Seurat (version 3) R package[16] : 1) integration of OD-BM, OD-spleen, OD-PB and LD-PB samples in a *multitissue seurat integrated dataset;* 2) integration of OD-spleen (OD1,2,4) and HS-spleen (HS1,2) in an *integrated spleen* dataset and 3) OD1-4 BM and all PB samples (OD1, OD2, OD4, LD1-6, mPB1-4) to create the *integrated mobilized peripheral blood dataset* (supplemental Figure 10).

The filtered count matrix from each dataset was independently normalized and had its HVGs determined[11]. Subsequently, integration was performed using 3,000 anchors found using the first 15 dimensions from the canonical correlation analysis. Following the integration, the data was scaled and dimensionality reductions were calculated, resulting in what is hence called the *multitissue seurat integrated dataset,* the *integrated spleen dataset* and the *integrated mobilized peripheral blood dataset.*

To verify that similar results could be obtained with a different method, in parallel, we also applied ComBat correction[10] (as implemented in Scanpy) to the *multitissue base normalized dataset*. This yielded a similar landscape with similar cluster identities and proportions (supplemental Figure 3G-H).

CITE-seq cell surface protein data analysis

Cell surface protein data was analyzed for OD3 (spleen) and OD4 (BM, spleen) as described in [17]. Cell surface protein data was quantified using CITE-seq-Count (version 1.4.3). Cells with total counts higher than 5,000, or fewer than 30 antibodies, or antibodies expressed in fewer than 3 cells were removed from downstream analysis. Raw protein count matrices underwent correction for cell-to-cell variation via normalization using the DSB (Denoised and Scaled by Background) algorithm [18]. DSB normalization quantified protein counts above background levels in individual cells and allowed for improved interpretability of protein expression. The dimensionality reduction techniques applied were the ones implemented on the 10x data. PCA, UMAP and Force Directed Graph (FDG) visualization were performed as below and DSB normalized expression values were plotted on the UMAP coordinates and on a heatmap. FDG was used for illustrating cell density. For batch correction of the cell surface protein data ComBat was used to integrate all donors. Finally, Leiden clustering was performed, and clusters were annotated using differential expression of known surface markers.

10x data - Dimensionality reduction and visualization

The dimensionality reduction techniques applied to the analysis datasets (supplemental Figure 10) were the ones implemented in the Scanpy package, with the exception for the *multitissue seurat integrated dataset* in which the implementations within the Seurat3 package were used. Specifically, these techniques were PCA, diffusion maps[19] and uniform manifold approximation and projection (UMAP)[20]. In addition, the ForceAtlas2 force-directed graph (FDG) algorithm[21] was applied to the k-nearest neighbor graph of the datasets to also provide further embeddings. Furthermore, the PCA coordinates generated for the *multitissue seurat integrated dataset* were imported to the *multitissue base normalized dataset* and used to compute the nearest neighbor's graph in this dataset before further downstream analysis.

10x data - Clustering

Cell communities (henceforth clusters) were defined via the Leiden algorithm (as implemented in Scanpy) at several resolutions. Silhouette plots and respective scores were calculated in order to evaluate and select which resolution to proceed with. All clustering resolutions yielded similar proportions of the four major hematopoietic lineage branches. Leiden resolution 1.2 was chosen for all further analyses downstream of the *multitissue base normalized dataset* as it yielded the best granularity within each branch and high silhouette scores. No clustering was performed

on the *integrated spleen dataset* and the *integrated mobilized peripheral blood dataset.* Instead, cells were annotated by transfer of the cluster labels from the *multitissue seurat integrated dataset* (supplemental Figure 10, see methods below).

Differential expression

Differential expression tests (Table S3) were performed on raw counts using the R packages DESeq2 [9] for Smart-seq2 sequencing data and edgeR [22] using the likelihood ratio test for the 10x sequencing data, with modeling the donor categorical as covariate where applicable (supplemental Figure 4B). Additionally, the Wilcoxon rank sum (WRS) test, as implemented in Scanpy, was used to test differential mRNA and cell surface protein expression between groups of cells. Prior to gene set enrichment analysis, differential gene expression lists were ranked based either on the Wald statistic (from DESeq2 results of Smart-seq2 data) or by the product of the sign of the fold change with the -log10 of the p-value (for all edgeR results where donor was used as covariate, supplemental Figure 4B) or by Wilcoxon rank sum test (Figure 4D,G, supplemental Figure 5D,11C).

Pseudotime

Pseudotime was calculated initially on the diffusion map of the *multitissue base normalized dataset* (integrating all OD samples and LD-PB) using diffusion pseudotime (DPT) [23] (as implemented in Scanpy). Upon analysis of the scores and the lineage marker plots cells scoring in pseudotime above 0.15 (1,578 cells) and 5 cells belonging to clusters 20, 25, 26 were identified as mature cell types and excluded and the diffusion map and DPT recalculated. The root cell was selected from the BM cells on cluster 4 (HSC/MPP1). DPT and a root cell in cluster 4 was also used for pseudotime calculation of ADT-data from CITE-seq of OD3 (BM) and OD4 (BM, spleen).

10x scRNA-seq data - Integration of cell cycle regressed data

An additional integration of the data from the OD-BM, OD-spleen, OD-PB and LD-PB samples was performed. As previously, we used Seurat for that purpose. However, in this integration the filtered counts were processed through a variance stabilizing transformation (sctransform), in lieu of the more commonly used steps of log-transformation. Additionally, using the vars.to.regress parameter, we regressed for number of counts, number of genes, fraction of mitochondrial genes and cell cycle. Downstream of these steps, the integration followed as previously described for the *multitissue seurat integrated dataset* leading to what we henceforth refer as the *multitissue base regressed dataset*. Using again the Leiden algorithm for clustering we

initially chose a Leiden resolution of 0.7, and identified 2 clusters (15,16) as mature, which we excluded. Proceeding to an additional round of re-clustering, at a resolution of 1.3 led us to exclude another cluster identified as mature (20). On a final round of reclustering we settled on the Leiden resolution of 1.4 as the final dataset partitioning.

Lineage characterization

Characterization of the clusters defined via Leiden clustering was achieved through i) ranked gene groups (by score) WRS tests using a one-versus-rest strategy, thus retrieving the most differentially expressed genes in a cluster relative to the remainder clusters; ii) the expression of known gene markers for specific lineages within the Leiden clusters was further investigated by plotting them onto the previously generated embeddings and verifying their co-localization with respect to the defined clusters. Clusters of My and Meg/Ery/Eo/Baso/MC (MEMB) lineages were further grouped into 'early' and 'late' progenitors (see Table S2c) based on their order along a diffusion pseudotime axis and progressive increase in marker gene as well as surface marker expression. Using pseudotime and cluster annotation, 1583 mature cells were excluded from the *multitissue seurat integrated dataset* (integrating ODs and LD-PB) prior to further downstream analysis steps (cell distribution, lineage scores, de novo pseudotime, cell cycle scores, CITE-seq analysis, comparison to HCA-BM data). Cluster 14 of the same dataset was relatively enriched for cells with a high fraction of mitochondrial reads, had relatively low read counts per cells and could not be assigned to any hematopoietic lineage using the strategy above, nor to any other cell lineage using the MSigDB C8 gene signatures. We thus termed it ND/LQ (Not Determined / Low quality) and excluded it from all downstream analyses.

Analysis of cell distribution

After exclusion of mature cells, cluster counts were normalized to 5000 total cells per donor and tissue for a standardized comparison of cluster/HSPC group composition between different sites. For statistical analysis of cluster/HSPC group composition, the normal distribution of each cluster/HSPC group was tested for each tissue using a Shapiro-Wilk test on the normalized cluster counts. Next, a one-way ANOVA with post-hoc Tukey multiple pairwise-comparisons was performed on normally distributed clusters/HSPC group. A Kruskal-Wallis test with Dunn's multiple comparison was used for not normally distributed clusters/HSPC groups. To visualize the different cell distributions in each tissue along the UMAP coordinates, a random 2500 cells were first selected for each donor and tissue to allow equal contribution of each sample.

Cells of the same tissue were combined and then a 2D kernel density estimation was performed using the stat_density_2d function of ggplot2.

Self-assembly manifolds (SAM) of HSC/MPPs

The subset of BM and spleen cells from ODs 1, 2 and 4 in the *multitissue seurat integrated dataset* that were defined as HSC/MPPs (Leiden clusters 0, 4, 5, 11, 21) was processed with the Self-assembling manifold (SAM) approach [24] with the aim of getting better resolution in cell segregation (supplemental Figure 10). This subset was corrected using Scanorama [25] before being processed with SAM, while disabling inbuilt standardization. K-means (k=2) clustering was subsequently computed after visual inspection of the UMAP plots.

Cell-cycle, Ery-Meg-priming, lineage/HSC and identity scores

The Scanpy implementation of the “score genes cell cycle” method presented by [11] was used to calculate S phase and G2-M phase scores, as well as the lineage and identity scores. To retrieve the gene lists used to calculate the Ery and Meg scores, differentially expressed genes between EryP (cluster 8) and MegP (cluster 22) were analyzed and the top50 genes (FDR <0.05, ranked by LFC) were extracted as being most characteristic of each population. For GMP-, MLP-, MEP- and HSC-scores, the top50 genes of published gene sets enriched in prospectively isolated HSPC subsets[26] were used. The gene lists used for the BM-type and SPL-type HSC/MPP identity scores were built from the overlap of significantly differentially expressed genes (FDR<0.05) between cluster SAM0-med and SAM1-extramed that were found in all ODs (OD1, 2, 4). For the medullary identity score all 72 overlapping genes were used (LFC>0 in each donor), whereas for the extramedullary identity score only the top 120 genes with LFC>0.3 in each donor were used. These gene lists (see Table S4) were then used as input in the score genes method (implemented in Scanpy) to calculate the Ery-Meg-priming, lineage/HSC and identity scores.

To quantify the proportions of HSC/MPPs with a BM- or SPL-type identity score, a ratio of the scaled score values (to be between 0 and 1) was calculated. The score ratio in transcriptionally identified HSC/MPPs from BM and/or spleen in our 10x dataset was used as a reference to compare the score ratio in other tissues from the same landscape or from independent datasets. Ratios of these scores above 1 and below 1 respectively indicated BM-type and spleen-type identities. To detect differential expression patterns of the signature genes defining the BM- and spleen-type identity (same as to calculate scores), the package DEGreport

(http://lpantano.github.io/DEGreport/) was used on the log-normalized UMI counts of HSC/MPPs (Leiden clusters 0, 4, 5, 11, 21) from different tissues.

Gene set enrichment analysis

Gene set enrichment analysis (GSEA) was performed with the GSEA software (v4.1) using pre-ranked gene lists (WRS or "edgeR-derived ranking score", as previously described). Enrichment was tested for C2 curated gene sets and hallmark gene sets of the MSigDB database v7.2 [27], published population-specific signatures [26,28], lineage-priming modules [29] as well as signatures of human BM LT- and ST-HSCs (Cabezas-Wallscheid et al., submitted) as reference gene sets. Further, two sets of genes previously found either up- or down-regulated in mouse HSCs after treatment with mobilizing agents compared to untreated HSCs [30] were used. All gene sets used in this study are listed in Table S4.

Cell projection and annotation transfer

For transferring annotations from a reference dataset to a target dataset of interest, we have used cell project (https://github.com/Iwo-K/cellproject), a tool which allows to map cell coordinates from a target dataset's embedding into a reference dataset's embedding, as well as the transference of selected annotations.

Wherever possible, scRNA-seq data was compared to the benchmark HCA-BM dataset[1], generated by 10x scRNA-seq of MNCs. We used the same single cells selected as $CD34^+$ (based on mRNA expression) in the published study [31]. To best compare with our $CD19^-CD34^+$ sorted cells, all CD19 expressing clusters (cluster "$CD34^+$ pre-B", "$CD34^+$ pre-PC", "$CD34^+$ pre-B cycling", "$CD34^+$ Lymphoid UNK" as labeled in the original publication) were first excluded from this dataset. We then used cell project to i) transfer cluster annotations (label transfer) from our *multitissue seurat integrated dataset* (BM cells subset) to the HCA-BM dataset filtered as described above (Table S2f). We also performed the reverse, ii) where we used the filtered HCA-BM dataset as a reference and label transferred its annotations to our full *multitissue seurat integrated dataset* (Table S2o). Similarly, as described for our own 10x scRNA-seq dataset, cluster counts were normalized to 5,000 total cells for each donor of the HCA-BM (Table S2f) to allow a standardized comparison of cluster/HSPC group composition to our own dataset.

The same approach of label transfer was also applied individually on the integrated spleen and integrated mobilized peripheral blood datasets. Here, the datasets were split into 'target datasets', comprising the iii) two HS spleen samples (Table S2l) and the iv) four G-CSF mobilized PB (Table S2j) respectively, and the remainder of cells

from each (OD spleens or BM plus PBs, respectively) constituting the reference datasets. Finally, and still using the same method, we projected v) single CD71^{+} HSC/MPPs on a reference set generated from reintegrating only the cells from our HSC/MPP clusters (0, 4, 5, 11, 21) from our *multitissue seurat integrated dataset*.

Estimation of cells actively dividing and cellular expansion modes in the MEMBP and MyP branch

The number of active cells in each cluster of the MEMBP and MyP branches was calculated as: (number of cells in the a given cluster) * (proportion of cells in that cluster in S-G2-M) (values for calculations taken from Table S2n). Then values were renormalized to 5,000 cells. We postulate that this is the minimum number of cells that is bound to divide at any given moment, as they have passed the R point of the cell cycle.

Based on transcriptomic characteristics, cell cycle assignment and pseudotime, we postulated 3 steps in both MEMBP and MyP differentiation: i) an early step (containing cells with no suspected differentiation potential towards other lineages and lowest pseudotime); ii) a late step (including the cluster with marker differentiation markers and the highest pseudotime), and iii) an intermediate step (defined as all the clusters in between early and late steps).

Specifically, in the multitissue integrated dataset, for the MEMPB branch, these were: Early step: MEP-MPP (cluster 10) + MEP1 (cluster 12); Intermediate step: MEP2 (cluster 2); Late step: EryP (cluster 8) + MkP (cluster 22) + Eo/Baso/MCP (cluster 17) + MEP_cycle (cluster 13). For the MyP branch, the steps were: Early step: CMP-MPP (cluster 7) + MyP1 (cluster 6); Intermediate step: MyP2 (cluster 9); Late step: MyP3 (cluster 16) + MyP4 (cluster 15).

The numbers of active cells were normalized to the Early Step of the respective branch. We then estimated the number of divisions occurring in each branch in the BM, with the assumption that from the respective early steps onwards all divisions are symmetric towards differentiation. This was obtained as follows:

Log2(Norm Active cells at late stage in BM) - Log2(Norm Active cells at early stage in BM).

The number of divisions between the early and late steps in the MEMBP and MyP branches was estimated respectively as 5.81 (standard deviation: 0.55) and 2.83 (standard deviation: 0.47). If the differentiation kinetics were similar to the BM, the number of cells expected at the late stage of differentiation in spleen and PB would be: $2^{(\text{number of divisions estimated from BM})}$ * number of active cells at early stage.

The number of active cells in each cluster of the MEMBP and MyP branches was also calculated for the integrated spleen datasets (using values in Table S2n). The raw values obtained for the OD spleens were very close to those obtained for the same samples in the multitissue integrated dataset. For better direct comparison to this dataset and the data in Figure 3D, the values of active differentiation cells were normalized to those found for OD-spleen in the multitissue dataset before being visualized in Figure 5D.

My, Ly, Ery and Meg single-cell differentiation assay

One day before the sort, MS5 cells at passage 10-13 [32] (imported from Prof Katsuhiko Itoh at Kyoto University) were seeded at 3000 cells per well of a 96-well flat-bottom plate (Nunc) in 100 µl Myelocult H5100 medium (Stem Cell Technologies) + 1% Pen/Strep (Life Technologies). The next day, medium was replaced by 100 µl/well StemPro-34 SFM medium (Life Technologies) supplemented with 3.5 % StemPro-34 nutrients (Life Technologies), SCF 100 ng/ml, Flt3-L 20 ng/ml, TPO 100 ng/ml, IL-6 50 ng/ml, IL-3 10 ng/ml, IL-11 50 ng/ml, GM-CSF 20 ng/ml, IL-2 10 ng/ml, IL-7 20 ng/ml (all Miltenyi Biotec), erythropoietin (EPO) 3 units/ml (Eprex, Janssen-Cilag), h-LDL 50 ng/ml (Stem Cell Technologies), 1% L-Glutamine (Life Technologies) and 1% Pen/Strep. Single phenotypic HSC/MPPs (from LD-BM, healthy LD-PB, ET LD-PB, b-thal LD-PB, OD-BM, OD-spleen or HS spleen) or $CD71^-$ and $CD71^+$ HSC/MPPs from LD-PB were index-sorted into each well and incubated at 37 °C for 2-3 weeks prior to analysis by flow cytometry (BM, spleen 3 weeks; PB 2 weeks). If necessary, medium was topped up with 50 µl StemPro medium plus supplements at the half time of culture. For colony harvest, cells from each well were transferred through a 30-40 µm plate filter (Pall Laboratory) into 96 U-bottom plates, to exclude co-cultured MS5 cells. Cells were stained with antibody panel (I) or (J) for 20 min at RT in the dark, washed once with PBS/FBS and resuspended in 100 µl PBS/FCS per well, and 50 µl were used for high-throughput flow cytometry analysis. For colonies from ET PB HSC/MPPs, the residual 50 µl were kept for genotyping. A single cell was defined as giving rise to a colony if the sum of cells detected in the $CD45^+$ and $GlyA^+$ gates was > 30 cells. Colony efficiency was calculated as the number of positive colonies divided by the number of seeded single cells. Individual colonies were identified as positive if they contained at least the indicated number of surface-marker positive cells: Ery ($CD45^-GlyA^+$) > 30 cells, Meg $CD41^+$ > 20 cells, My colonies (sum of $CD45^+CD14^+$ and $CD45^+CD15^+$) > 30 cells, NK colonies ($CD45^+CD56^+$) > 30 cells (also see gating strategy in supplemental Figure 7C). Colonies were identified as undifferentiated (undiff) if the sum of cells in $CD45^+$ and $GlyA^+$ gates exceeded 30, but the cells could not be

identified as positive for any lineage (Ery, Meg, My, NK) using the criteria above. For visualization purposes, ‘EryMegMy’ colonies are the sum of EryMegMy and MegMy colonies, the latter of which only appeared at proportions <3% in 7 of the samples. For the same reason the low fraction of EryNk (<1% of all colonies in one sample only) were combined with EryMegNK colonies and are labeled as ‘EryMegNK’. The median colony size (Figure 7D) was determined for each donor individually as: median of the cell counts in the My gates (sum of $CD45^{+}CD14^{+}$ and $CD45^{+}CD15^{+}$) for all positive colonies containing myeloid cells or in the $CD45^{-}GlyA^{+}$ gate for all positive colonies containing erythroid cells. For time to first division experiments, single phenotypic MEPs from LD-BM and LD-PB or single $CD71^{+}$ phenotypic HSC/MPPs were directly sorted into round-bottom 96 well plates containing the same medium as above. Cells were counted under an inverted microscope every 24 h for 4 days of culture. Empty wells were excluded from the analysis. After 2 weeks, colonies were harvested and analyzed as described above.

Genotyping ET colonies

For each colony grown from ET PB HSC/MPPs, half of the cells were used for genotyping. For DNA extraction, colonies were transferred into 96-well PCR plates, spun down, resuspended in 10 µl 0.2M NaOH and incubated for 5 min. After addition of 90 µl Tris-HCL pH7.5 genomic DNA was stored at -20 °C until genotyping.

Genotyping was performed by allele-specific PCR analysis as described in [33]. Briefly, DNA was amplified using one reverse primer (5’CTGAATAGTCCTACAGTGTTTTCAGTTTCA3’) and two forward primers (one annealing upstream to the mutation site: 5’ATCTATAGTCATGCTGAAAGTAGGAGAAAG3’; and one binding the c.1849G>T mutation at 3’end (5’CTGAATAGTCCTACAGTGTTTTCAGTTTCA3’). PCR products – of 364-bp and 203-bp, respectively –, were separated by gel electrophoresis and the mutational status was determined based on the presence or absence of the 203-bp product. The 364-bp product served as internal control of the reaction.

Single-cell Ery/Meg assay

Single cells of the indicated populations (all isolated from LD-PB) were sorted into each well of a U-bottom plate containing 100 µl StemSpan SFEM II (Stem Cell Technologies) supplemented with SCF 100 ng/ml, TPO 100 ng/ml, IL-6 10 ng/ml, IL-3 10 ng/ml, EPO 1 unit/ml, h-LDL 40 ng/ml and 1% Pen/Strep. After 2 weeks of culture at 37 °C, the plate was spun down and single-cell derived colonies were stained with antibody panel (K) for 20 min at RT in the dark, washed once with PBS/FBS and

resuspended in 100 µl PBS/FCS per well for high-throughput flow cytometry analysis. A single cell was defined as giving rise to a colony if the sum of cells detected in the $CD45^+$ and $GlyA^+$ gates was > 30 cells. Individual colonies were identified as positive if they contained at least the indicated number of surface-marker positive cells: Ery ($CD45^-GlyA^+$) > 30 cells, Meg ($GlyA^-CD41a^+CD42b^+$) > 20 cells, My ($CD45^+CD11b^+$) > 30 cells (also see gating strategy in supplemental Figure 8B). Colonies were identified as undifferentiated (undiff) if the sum of cells in $CD45^+$ and $GlyA^+$ gates exceeded 30, but the cells could not be identified as positive for any lineage (Ery, Meg, My) using the criteria above.

Serial replating assay

For serial replating assays, 1,500 - 2,000 $CD71^-$ HSC/MPPs, 800 - 2,000 $CD71^+$ HSC/MPPs or 500 - 2,000 E-MEPs were sorted into 300 µl PBS + 3% FBS in 1.5 ml microcentrifuge tubes. After centrifugation at 500xg for 5 min, the cells were resuspended in 100 µl PBS/FBS and added to 2.5 ml Methocult H4034 Optimum medium (Stem Cell Technologies) supplemented with Flt3-L 10 ng/ml, IL-6 10 ng/ml (Miltenyi Biotec) and 1% Pen/Strep (Life Technologies). Next, samples were plated as technical duplicates into 6 well SmartDishes (Stem Cell Technologies) following the manufacturer's protocol. After 2 weeks of culture at 37 °C, the number and type of colony forming units was determined by visual inspection of photographs taken with the StemVision instrument (Stem Cell Technologies). Colonies were harvested on day 14 and 15,000 - 40,000 cells from each well were replated into a secondary methylcellulose culture. Secondary cultures were incubated for 2 weeks culture at 37 °C before determination of colony numbers and types. For one of the replicates, multiple LD-PB donors were combined prior to sort, whereas all other replicates were performed with LD-PB from individual donors. The sum of the technical duplicates was used for analysis.

Megacult assay

The Megacult^{TM}-C kit (Stem Cell Technologies) was used. In brief, 150 - 7,700 $CD71^-$ HSC/MPPs or 500 - 4,100 $CD71^+$ HSC/MPPs from LD-PB were mixed with 1 ml Megacult^{TM}-C medium supplemented with 1% Pen/Strep, 40 µg/ml h-LDL, 0.6 ml Collagen Solution and seeded as technical duplicates. After 12 days culture at 37 °C, colonies were fixed and stained for CD41 according to the manufacturer's protocol. Colonies were scored using a bright field microscope and identified as follows: small Meg colony: 3-20 $CD41^+$ cells; intermediate Meg colony: 21-49 $CD41^+$ cells; large Meg colony ≥ 50 $CD41^+$ cells; mixed colony: ≥ 20 cells containing $CD41^+$ and $CD41^-$ (only

nuclei staining) cells; non Meg colonies ≥ 20 CD41^{-} cells. For one of the replicates, multiple LD-PB donors were combined prior to sort, whereas all other replicates were performed with PB from individual donors. The average values of technical duplicates were used.

Xenotransplantation assays

Different cell doses of indicated populations (445 - 21,700 HSC/MPPs; 100 - 8,000 CD71^{-} HSC/MPPs; 165 - 10,700 CD71^{+} HSC/MPPs; all isolated from LD-PB; or 50 - 36,500 from cord blood HSC/MPPs as controls, see Table S5) were transplanted into age-matched female NSG mice. One day before transplantation, 12–16 weeks old mice were pre-conditioned by sublethal irradiation (2.4 Gy), and cells were sorted into X-VIVO 10 Serum free hematopoietic cell medium (Lonza) supplemented with 1% BSA (Roche), 1% L-glutamine, 1% Pen/Strep (both Life Technologies), SCF 100 ng/ml, Flt3-L 100 ng/ml, TPO 50 ng/ml, IL-7 10 ng/ml (all Miltenyi Biotec) and kept in medium overnight at 37 °C. The next morning, mice were anaesthetized with isoflurane and different cell doses of indicated populations (Table S5) were injected intrafemorally in 25 µl PBS + 0.1% Pen/Strep. Buprenorphine (0.1 mg/kg, Animalcare) was injected subcutaneously as analgesic. The cohorts transplanted for 2 and 4 weeks with CD71^{-} and CD71^{+} HSC/MPPs, were given intraperitoneal injections of hEPO (20 units/injection, Eprex, Janssen-Cilag) twice a week to promote human red blood cell differentiation [2]. Mice transplanted with varying doses of CD71^{-} HSC/MPPs and CD71^{+} HSC/MPPs were analyzed at 2, 4 and 8 weeks post transplantation. Mice transplanted with PB HSC/MPPs were analyzed at 8 and 16 weeks post transplantation. For some experiments, cord blood (CB) phenotypic HSC/MPPs were transplanted as positive controls and analyzed at 8 and 16 weeks respectively (engraftment is summarized in Table S5).

At the final analysis time points, the injected femur was harvested separately from the non-injected femur and both tibias. Bone marrow was flushed with IMDM + 5% FBS. Cells from the cohorts that received CD71^{-} and CD71^{+} HSC/MPPs and were injected with EPO were stained with antibody panel (M). The cells from all other cohorts (transplanted with CD71^{-} and CD71^{+} HSC/MPPs for 8 or 16 weeks, and all mice transplanted with PB HSC/MPPs) were stained with antibody panel (L).

To allow confident detection of human leukocyte engraftment, we stained all samples with two distinct antibodies against CD45. Cells were only considered human leukocytes if they were positive for both CD45 antibodies (CD45^{++}, see supplemental Figure 7A, 8K) as previously described in [8]. Threshold of engraftment was set to ≥ 0.01 % CD45^{++} cells with at least 30 cells recorded in the CD45^{++} gate. Cells were counted

as My lineage if $CD45^{++}CD33^{+} \geq 20$ cells; Ly lineage if $CD45^{++}CD19^{++} \geq 20$ cells (positive for two distinct CD19 antibodies); Ery lineage: $CD45^{-}GlyA^{+} \geq 20$ cells or $CD45^{-}CD71^{+}GlyA^{+} \geq 20$ cells. Example gating strategies are shown in supplemental Figure 7A, 8K. An estimate of the number of repopulating cells within each transplanted subpopulation was calculated from the engraftment of limiting cell doses using Extreme Limiting Dilution Analysis (ELDA) statistics [34].

Sample size determination and study design

In all experiments the maximal possible sample size was used, considering limited the availability of primary human samples and rarity of the HSPC populations. Unless otherwise indicated, single-cell experiments were repeated at least three times with independent biological samples. For all single cell functional assays, at least 40 single cells were seeded per condition and biological replicate to ensure sufficient power of the assays. For limiting dilution assay transplantation experiments, a minimum of 10 animals per population and at least three cell doses were analyzed. No specific randomization or blinding of NSG mice was performed as all mice shared the same genetic background and were age-matched females.

SUPPLEMENTAL NOTE 1

In this study we have leveraged the use of hematopoietic samples from multiple sources to study HSPC dynamics at extramedullary sites. This included analysis of organ donors (OD) and healthy living donors (LD) derived tissues as well as meta-analysis of published BM data[31] (HCA-BM) to benchmark our findings. We have indicated the source of the material for every datapoint and compared tissues from the same source wherever possible. For most parameters, we have found no significant differences between tissues isolated from different sources. Here we discuss the few instances of measurable differences in HSPC composition in a specific tissue across different sources.

Comparing OD-PB to LD-PB we noticed a slight increase in the abundance of transcriptionally defined early MyPs and HSC/MPPs (HSC/MPP2 and HSC/MPP3 clusters) in OD-PB compared to LD-PB (supplemental Figures 2B, 11A). This was associated with an increase in the BM- to spleen-type ratio (supplemental Figure 11B), particularly marked for one donor OD2, indicating an increased proportion of BM-type HSC/MPPs in OD-PB. Gene sets associated with LT-HSCs or up-regulated in mouse HSCs after treatment with mobilizing drugs[30] were enriched in HSC/MPPs from ODs compared to HSC/MPPs from LDs (supplemental Figure 11C). None of the ODs used for 10x scRNA-seq did show any clinical records of cardiovascular or other chronic diseases that could have influenced circulation of CD34^{+} cells. Thus, we believe this was the result of mobilization of BM HSC/MPPs due to trauma and brain death associated cytokine and neurotransmitter release[35–38] in certain ODs. In line with a potential mobilization of cytokine-activated progenitors, we observe that several progenitor cell types are more cycling in OD-PB compared to LD-PB, albeit still less than compared to the matched BM progenitors (supplemental Figure 4A, 11D). Furthermore, the ratio of early MEMBP to early MyP progenitors is lower in OD-PB compared to LD-PB (supplemental Figure 11E), and more similar to the ratio observed in BM and G-CSF mobilized PB (supplemental Figure 11F), suggesting an increased circulation of myeloid progenitors in OD-PB.

We have also used the benchmark HCA-BM data to verify our findings on extramedullary HSPCs. When directly comparing scRNA-seq from OD-BM to the HCA-BM we noticed a slight increase in cells classified as HSC/MPP and late My progenitors in our OD-BM (supplemental Figure 11D). These could be equally attributed to: 1) distinct sources of BM harvested (sternum vs femur), 2) distinct physiological status of

the donors (brain or circulatory death vs healthy and alive), 3) different methods of purification of $CD34^+CD19^-$ cells (flow cytometry vs mRNA expression). We did not observe any significant difference in the proliferative and differentiation capacity of HSC/MPPs or MEPs purified from either OD or LD (supplemental Figures 4C-E, 7D), indicating no major changes in HSC subsets function between these two sources. Thus, observed differences have no impact on the conclusions of our study on the features of extramedullary hematopoiesis (Figures 2B-D vs supplemental Figures 2F-H; Figure 4I; Figure 6B vs S7B).

SUPPLEMENTAL TABLES

Table S1. Information on the deceased organ donors (ODs) and living donors (LDs) used in this study.

Table S2. Marker genes, cluster counts and cell scores for all datasets. Table content is summarized in the first worksheet.

Table S3. Results of all differential expression and differential pattern analysis performed on RNA-seq and cell surface protein data. Table content is summarized in the first worksheet.

Table S4. List of all gene sets used for GSEA and cell scores and results of all gene set enrichment analyses performed. Table content is summarized in the first worksheet.

Table S5. List of NSG mice used for xenotransplants.

mouse	Cell population transplanted	Cell number transplanted	Weeks of engraftment	Engrafted?	Engraftment [% hCD45++]	Lineage composition		
						% Ery	% My	% Ly
1	LD-PB HSC/MPP	395	8	no	-	-	-	-
2		395	8	yes	1.840	0.00	2.32	97.68
3		395	8	no	-	-	-	-
4		395	8	yes	0.122	0.00	0.00	100.00
5		445	8	no	-	-	-	-
6		1805	8	yes	0.171	0.01	3.66	96.33
7		2253	8	no	-	-	-	-
8		2253	8	yes	0.024	0.00	51.53	48.47
9		2253	8	yes	2.370	0.00	1.02	98.97
10		2253	8	no	-	-	-	-
11		5250	8	no	-	-	-	-
12		5250	8	no	-	-	-	-
13		5250	8	yes	0.031	0.00	4.62	95.38
14		5250	8	yes	1.030	0.00	4.76	95.24
15		21700	8	yes	2.460	0.02	1.73	98.26
16		131	16	yes	0.039	0.00	0.00	100.00
17		307	16	no	-	-	-	-
18		307	16	no	-	-	-	-
19		939	16	no	-	-	-	-
20		3075	16	no	-	-	-	-
21		3075	16	yes	0.160	0.00	1.64	98.36
22		3257	16	no	-	-	-	-
23		5124	16	yes	0.044	0.00	0.00	100.00
24		6766	16	yes	0.023	0.00	97.68	2.32
25		6766	16	no	-	-	-	-
26	LD-PB $CD71^-$ HSC/MPP	343	2	yes	0.019	0.00	96.56	3.44
27		500	2	no	-	-	-	-
28		600	2	no	-	-	-	-
29		926	2	no	-	-	-	-
30		926	2	no	-	-	-	-
31		3017	2	yes	0.034	0.00	25.99	74.01
32		3492	2	no	-	-	-	-
33		3500	2	no	-	-	-	-
34		3500	2	no	-	-	-	-
35		5000	2	yes	0.149	0.02	85.91	14.07
36		5000	2	yes	0.014	0.00	7.69	92.31
37		750	4	yes	0.016	0.00	32.60	67.40
38		798	4	yes	0.413	0.00	49.23	50.77
39		850	4	yes	0.017	0.00	98.96	1.04
40		1453	4	yes	0.016	0.00	69.68	30.32
41		1500	4	no	-	-	-	-
42		1500	4	yes	0.073	0.00	97.06	2.94
43		1600	4	yes	0.226	0.00	89.19	10.81
44		1600	4	yes	0.038	0.00	90.71	9.29
45		8000	4	no	-	-	-	-
46		5000	8	yes	1.36	0.00	20.61	79.39
47		5000	8	yes	4.46	0.00	1.86	98.14
48		5000	8	yes	0.662	0.00	0.29	99.71
49		100	8	no	-	-	-	-
50		100	8	no	-	-	-	-
51		100	8	no	-	-	-	-
52		1300	8	yes	8.57	0.00	3.23	96.77
53		1300	8	yes	0.081	0.00	97.25	2.75
54		1300	8	yes	0.028	0.00	25.88	74.12
55		1300	8	no	-	-	-	-
56		150	16	no	0.000	-	-	-
57		150	16	no	0.002	-	-	-
58		150	16	no	0.001	-	-	-
59		4000	16	yes	0.045	0.00	1.7	98.3

60		4000	16	no	0.002	-	-	-
61		4000	16	no	0.002	-	-	-
62		1000	16	no	0.001	-	-	-
63		1000	16	no	0.000	-	-	-
64		1000	16	yes	0.014	0.00	14.5	85.5
65		3000	16	yes	0.222	0.01	28.88	71.12
66		3000	16	no	0.001	-	-	-
67		700	16	yes	0.013	0.00	96.08	3.92
68		165	2	no	-	-	-	-
69		500	2	no	-	-	-	-
70		600	2	no	-	-	-	-
71		2000	2	no	-	-	-	-
72		2000	2	no	-	-	-	-
73	LD-PB	3031	2	no	-	-	-	-
74	CD71$^+$	3689	2	no	-	-	-	-
75	HSC/MPP	5000	2	no	-	-	-	-
76		7300	2	no	-	-	-	-
77		10700	2	no	-	-	-	-
78		656	4	no	-	-	-	-
79		750	4	no	-	-	-	-
80		789	4	no	-	-	-	-
81		850	4	no	-	-	-	-
82		1500	4	no	-	-	-	-
83		1500	4	no	-	-	-	-
84		1600	4	no	-	-	-	-
85		5200	4	no	-	-	-	-
86		8000	4	no	-	-	-	-
87		36500	8	yes	63.5	0.01	1.14	98.85
88		36500	8	yes	49.6	1.28	6.06	92.66
89		50	16	yes	0.024	0.00	0.00	100
90		50	16	no	0.002	-	-	-
91		50	16	yes	0.175	0.00	78.31	21.69
92		250	16	yes	62.8	0.08	1.66	98.26
93	CB	250	16	yes	7.37	0.00	2.56	97.44
94	HSC/MPP	250	16	yes	58.7	0.04	1.71	98.25
95		500	16	yes	78.7	0.42	2.85	96.74
96		500	16	no	0.005	-	-	-
97		500	16	yes	50.3	0.13	2.55	97.32
98		1500	16	yes	20.9	0.01	1.49	98.51
99		1500	16	yes	47.5	0.19	2.78	97.03
100		15000	16	yes	73.6	2.56	2.59	94.85

Table S6. List of antibodies used for flow cytometry.

Antigen	Conjugate	Clone	Company	Cat #	Dilution
CD3	APC/Cy7	HIT3a	BioLegend	300318	1:100
	FITC	HIT3a	BD Pharmingen	555339	1:500
CD5	PE	UCHT2	BD Pharmingen	561897	1:300
CD7	BV421	M-T701	BD Horizon	562635	1:100
CD10	APC	HI10a	BioLegend	312210	1:100
	BV421	HI10a	BD Horizon	562902	1:100
CD11b	APC/Cy7	ICRF44	BioLegend	301342	1:300
CD14	PE-Cy7	M5E2	BioLegend	301814	1:1000
	APC/Cy7	HCD14	BioLegend	325619	1:1000
CD15	PE-Cy7	M5E2	BioLegend	301814	1:1000
	BV421	MC-480	BioLegend	125614	1:200
CD19	Alexa Fluor 700	HIB19	BioLegend	302226	1:300
	APC/Fire750	HIB19	BioLegend	302258	1:100
	BV785	SJ25C1	BioLegend	363028	1:600
	FITC	HIB19	BioLegend	302206	1:200
CD33	APC	P67.6	BD Pharmingen	345800	1:200
CD34	APC	581	BD Pharmingen	555824	1:100
	APC-Cy7	581	BioLegend	343514	1:100
CD36	APC	5-271	BioLegend	336208	1:300
CD38	PE/Cy7	HIT2	BioLegend	303516	1:100
CD41	APC	HIP8	BioLegend	303709	1:200
	BV510	HIP8	BD Horizon	563250	1:200
	FITC	HIP8	BioLegend	303704	1:1000
CD42b	APC	HIP1	BioLegend	303912	1:200
CD45	BV510	HI30	BioLegend	304036	1:500
	PE-Cy5	HI30	BioLegend	304010	1:300
	BV605	2D1	BioLegend	368524	1:100
CD45RA	Alexa Fluor 700	HI100	BioLegend	304120	0.25
	FITC	HI100	BD Pharmingen	555488	1:100
	PE	HI100	BioLegend	304108	1:200
	BV421	HI100	BioLegend	304129	1:100
CD49f	PE-Cy5	GoH3	BD Pharmingen	551129	1:100
CD56	APC	HCD56	BioLegend	318310	1:200
CD90	APC	5.00E+10	BD Pharmingen	559869	1:100
	PE	5.00E+10	BioLegend	328110	1:50
	FITC	5.00E+10	BioLegend	328108	1:50
CD71	FITC	CY1G4	BioLegend	334104	1:1000
	PE	CY1G4	BioLegend	334106	1:1000
	PerCP/Cy5.5	CY1G4	BioLegend	334113	1:800
	BV650	CY1G4	BioLegend	334115	1:100
CD105	BV650	266	BD Horizon	563466	1:50
CD123	BV785	6H6	BioLegend	306032	1:100
CD117 (c-Kit)	BV650	266	BD Horizon	563466	1:50
CD135	Alexa Fluor 647	4G8	BD Pharmingen	563494	1:50
	PE	BV10A4H2	BioLegend	313305	1:50
CD370 (CLEC9A)	PE	8F9	BioLegend	353804	1:75
GlyA	PE	GA-R2 (HIR2)	BD Pharmingen	340947	1:1000
	PE-Cy7	HI264	BioLegend	349112	1:300
	BV421	GA-R2 (HIR2)	BD Horizon	562938	1:1000
KI-67	FITC	B56	BD Pharmingen	556026	1:50

Table S7. List of antibodies used for CITE-seq (Total-Seq^{TM} A, BioLegend).

	Clone	Specificity	Barcode	DNA Barcode
ADT_A0145	Ber-ACT8	CD103	145	GACCTCATTGTGAAT
ADT_A0095	RTK4530	IgG2b, k Isotype Ctrl	95	GATTCTTGACGACCT
ADT_A0167	E11	CD35	167	ACTTCCGTCGATCTT
ADT_A0215	11C1	CD268 (BAFF-R, BAFFR)	215	CGAAGTCGATCCGTA
ADT_A0085	BC96	CD25	85	TTTGTCCTGTACGCC
ADT_A0161	ICRF44	CD11b	161	GACAAGTGATCTGCA
ADT_A0147	DREG-56	CD62L	147	GTCCCTGCAACTTGA
ADT_A0007	29E.2A3	CD274 (B7-H1, PD-L1)	7	GTTGTCCGACAATAC
ADT_A0087	UCHL1	CD45RO	87	CTCCGAATCATGTTG
ADT_A0159	L243	HLA-DR	159	AATAGCGAGCAAGTA
ADT_A0149	HP-3G10	CD161	149	GTACGCAGTCCTTCT
ADT_A0080	RPA-T8	CD8a	80	GCTGCGCTTTCCATT
ADT_A0050	HIB19	CD19	50	CTGGGCAATTACTCG
ADT_A0140	G025H7	CD183	140	GCGATGGTAGATTAT
ADT_A0352	AER-37 (CRA-1)	Fc epsilon RI alpha	352	CTCGTTTCCGTATCG
ADT_A0148	G043H7	CD197 (CCR7)	148	AGTTCAGTCAACCGA
ADT_A0083	3G8	CD16	83	AAGTTCACTCTTTGC
ADT_A0375	M1310G05	IgG Fc	375	CTGGAGCGATTAGAA
ADT_A0070	GoH3	CD49f	70	TTCCGAGGATGATCT
ADT_A0423	590H11G1E3	MERTK	423	TCCTGCATGTACCCA
ADT_A0100	2H7	CD20	100	TTCTGGGTCCCTAGA
ADT_A0088	EH12.2H7	CD279 (PD-1)	88	ACAGCGCCGTATTTA
ADT_A0132	AY13	EGFR	132	GCTTAACATTGGCAC
ADT_A0063	HI100	CD45RA	63	TCAATCCTTCCGCTT
ADT_A0236	RTK2071	IgG1, k Isotype Ctrl	236	ATCAGATGCCCTCAT
ADT_A0143	G034E3	CD196	143	GATCCCTTTGTCACT
ADT_A0126	clone 7	CD133	126	TGGTAACGACAGTCC
ADT_A0188	ASL-32	CD66a/c/e	188	GGGACAGTTCGTTTC
ADT_A0367	TS1/8	CD2	367	TACGATTTGTCAGGG
ADT_A0240	RTK4174	IgG2c, k Isotype Ctrl	240	TCCAGGCTAGTCATT
ADT_A0101	9E2	CD335	101	ACAATTTGAACAGCG
ADT_A0205	15-2	CD206 (MMR)	205	TCAGAACGTCTAACT
ADT_A0123	9C4	CD326 (Ep-CAM)	123	TTCCGAGCAAGTATC
ADT_A0206	7-239	CD169	206	TACTCAGCGTGTTTG
ADT_A0353	HIP8	CD41	353	ACGTTGTGGCCTTGT
ADT_A0371	P1E6-C5	CD49b	371	GCTTTCTTCAGTATG
ADT_A0370	201A	CD303	370	GAGATGTCCGAATTT
ADT_A0369	TS2/16	CD29	369	GTATTCCCTCAGTCA
ADT_A0366	12G5	CD184 (CXCR4)	366	TCAGGTCCTTTCAAC
ADT_A0374	MEM-108	CD98	374	GCACCAACAGCCATT
ADT_A0363	G077F6	CD124	363	CCGTCCTGATAGATG
ADT_A0199	7C9	Siglec-8	199	CTTCTCCTCAGCAAT
ADT_A0362	7D4-6	CD309/VEGFR2	362	TTCACGCAGTAAGAT
ADT_A0357	CD43-10G7	CD43	357	GATTAACCAGCTCAT
ADT_A0351	BV10A4H2	CD135 (Flt-3/Flk-2)	351	CAGTAGATGGAGCAT
ADT_A0207	8F9	CD370 (CLEC9A/DNGR1)	207	CTGCATTTCAGTAAG
ADT_A0196	HIR2	CD235ab	196	GCTCCTTTACACGTA
ADT_A0181	Bu32	CD21	181	AACCTAGTAGTTCGG
ADT_A0176	A1	CD39	176	TTACCTGGTATCCGT
ADT_A0163	M80	CD141	163	GGATAACCGCGCTTT
ADT_A0162	10.1	CD64	162	AAGTATGCCCTACGA
ADT_A0359	HB15e	CD83	359	CCACTCATTTCCGGT
ADT_A0372	VI-PL2	CD61	372	AGGTTGGAGTAGACT
ADT_A0358	GHI/61	CD163	358	GCTTCTCCTTCCTTA
ADT_A0360	108-17	CD357 (GITR)	360	ACCTTTCGACACTCG
ADT_A0373	5A6	CD81	373	GTATCCTTCCTTGGC
ADT_A0169	F38-2E2	CD366 (Tim-3)	169	TGTCCTACCCAACTT
ADT_A0168	QA17A04	CD57	168	AACTCCCTATGGAGG
ADT_A0171	C398.4A	CD278 (ICOS)	171	CGCGCACCCATTAAA
ADT_A0156	DX2	CD95	156	CCAGCTCATTAGAGC
ADT_A0146	FN50	CD69	146	GTCTCTTGGCTTAAA

ADT_A0005	2D10	CD80	5	ACGAATCAATCTGTG
ADT_A0055	MI15	CD138	55	ACTCTTTCGTTTACG
ADT_A0027	113-16	CD70	27	CGCGAACATAAGAAG
ADT_A0069	RCR-401	CD201 (EPCR)	69	GTTTCCTTGACCAAG
ADT_A0136	MHM-88	IgM	136	TAGCGAGCCCGTATA
ADT_A0361	p282 (H19)	CD59	361	AATTAGCCGTCGAGA
ADT_A0071	L291H4	CD194	71	AGCTTACCTGCACGA
ADT_A0034	UCHT1	CD3	34	CTCATTGTAACTCCT
ADT_A0046	SK1	CD8	46	GCGCAACTTGATGAT
ADT_A0165	1D11	CD314 (NKG2D)	165	CGTGTTTGTTCCTCA
ADT_A0006	IT2.2	CD86	6	GTCTTTGTCAGTGCA
ADT_A0141	J418F1	CD195	141	CCAAAGTAAGAGCCA
ADT_A0160	L161	CD1c	160	GAGCTACTTCACTCG
ADT_A0386	CD28.2	CD28	386	TGAGAACGACCCTAA
ADT_A0387	1D3	TSLPR (TSLP-R)	387	CAGTCCTCTCTGTCA
ADT_A0023	SKII.4	CD155 (PVR)	23	ATCACATCGTTGCCA
ADT_A0390	A019D5	CD127 (IL-7Ra)	390	GTGTGTTGTCCTATG
ADT_A0066	CD7-6B7	CD7	66	TGGATTCCCGGACTT
ADT_A0061	104D2	CD117	61	AGACTAATAGCTGAC
ADT_A0062	HI10a	CD10	62	CAGCCATTCATTAGG
ADT_A0052	P67.6	CD33	52	TAACTCAGGGCCTAT
ADT_A0029	BJ40	CD48	29	CTACGACGTAGAAGA
ADT_A0024	TX31	CD112 (Nectin-2)	24	AACCTTCCGTCTAAG
ADT_A0073	IM7	CD44	73	TGGCTTCAGGTCCTA
ADT_A0033	HI186	CD52	33	CTTTGTACGAGCAAA
ADT_A0394	CY1G4	CD71	394	CCGTGTTCCTCATTA
ADT_A0393	S-HCL-1	CD22	393	GGGTTGTTGTCTTTG
ADT_A0392	W6D3	CD15	392	TCACCAGTACCTAGT
ADT_A0124	WM59	CD31	124	ACCTTTATGCCACGG
ADT_A0081	M5E2	CD14	81	TCTCAGACCTCCGTA
ADT_A0089	A15153G	TIGIT	89	TTGCTTACCGCCAGA
ADT_A0102	BM16	CD294	102	TGTTTACGAGAGCCC
ADT_A0048	2D1	CD45	48	TCCCTTGCGATTTAC
ADT_A0072	RPA-T4	CD4	72	TGTTCCCGCTCAACT
ADT_A0054	581	CD34	54	GCAGAAATCTCCCTT
ADT_A0189	C1.7	CD244 (2B4)	189	TCGCTTGGATGGTAG
ADT_A0047	5.1H11	CD56	47	TCCTTTCCTGATAGG
ADT_A0144	J252D4	CD185	144	AATTCAACCGTCGCC
ADT_A0385	TS1/18	CD18	385	TATTGGGACACTTCT
ADT_A0214	FIB504	integrin b7	214	TCCTTGGATGTACCG
ADT_A0219	GIR-208	CD119 (IFN-g R a chain)	219	TGTGTATTCCCTTGT
ADT_A0131	16G5	Cadherin 11	131	CGTTGCCATTAACCA
ADT_A0180	ML5	CD24	180	AGATTCCTTCGTGTT
ADT_A0398	9-4D2-1E4	CD115	398	AATCACGGTCCTTGT
ADT_A0135	67A4	CD324 (E-Cadherin)	135	ATCCTTCTCCCTTTC
ADT_A0164	51.1	CD1d	164	TCGAGTCGCTTATCA
ADT_A0242	K036C2	CD192	242	GAGTTCCCTTACCTG
ADT_A0172	9F.8A4	CD275 (B7-H2, B7-RP1, ICOSL)	172	GTTAGTGTTAGCTTG
ADT_A0068	43A3	CD105	68	ATCGTCGAGAGCTAG
ADT_A0400	BV9	CD144	400	TCCACTCATTCTGTA
ADT_A0397	5E8	CD193	397	ACCAATCCTTTCGTC
ADT_A0383	JS11	CD55	383	GCTCATTACCCATTA
ADT_A0187	CB3-1	CD79b	187	ATTCTTCAACCGAAG
ADT_A0166	6/40c	CD66b	166	AGCTGTAAGTTTCGG
ADT_A0064	6H6	CD123	64	CTTCACTCTGTCAGG
ADT_A0053	S-HCL-3	CD11c	53	TACGCCTATAACTTG
ADT_A0218	AK4	CD62P (P-Selectin)	218	CCTTCCGTATCCCTT
ADT_A0216	HIP1	CD42b	216	TCCTAGTACCGAAGT
ADT_A0217	HA58	CD54	217	CTGATAGACTTGAGT
ADT_A0428	9F4	Tim-4	428	CGTCATATAGTATGG
ADT_A0427	94b/FOLR2	Folate Receptor beta	427	TGTGGCTAGTCAGTT
ADT_A0382	MEM-166	CD177	382	AGTATGGAGCCATAT
ADT_A0245	STA	CD106	245	TCACAGTTCCTTGGA

ADT_A0233	MHN3-21	Notch 3	233	CTATTGGACGTATCT
ADT_A0401	H037G3	CD301	401	ACCTAGAAATCAGCA
ADT_A0404	H5C6	CD63	404	GAGATGTCTGCAACT
ADT_A0248	HAE-1f	CD62E	248	CTCCCTGTGGCTTAA
ADT_A0384	IA6-2	IgD	384	CAGTCTCCGTAGAGT
ADT_A0419	3F3	CD72	419	CAGTCGTGGTAGATA
ADT_A0446	VIMD2	CD93	446	GCGCTACTTCCTTGA
ADT_A0409	17G10.2	CD85g (ILT7)	409	TGTCAGTTCCTATGA
ADT_A0408	15-414	CD172a	408	CGTGTTTAACTTGAG
ADT_A0405	HTA125	CD284 (TLR4)	405	GCTTAGCTGTATCCG
ADT_A0406	12C2	CD304	406	GGACTAAGTTTCGTT
ADT_A0407	5-271	CD36	407	TTCTTTGCCTTGCCA
ADT_A0244	CBR-IC2/2	CD102	244	TGACCTTCCTCTCCT
ADT_A0224	IP26	TCR alpha/beta	224	CGTAACGTAGAGCGA
ADT_A0246	TU27	CD122	246	TCATTTCCTCCGATT
ADT_A0237	G0114F7	IgG1, λ Isotype Ctrl	237	GGGAGCGATTCAACT
ADT_A0247	1A1	CD267 (TACI)	247	AGTGATGGAGCGAAC
ADT_A0155	H4A3	CD107a	155	CAGCCCACTGCAATA
ADT_A0134	P1H12	CD146	134	CCTTGGATAACATCA
ADT_A0213	MHN1-519	Notch 1	213	AATCTGTAGTGCGTT
ADT_A0575	TS2/7	CD49a	575	ACTGATGGACTCAGA
ADT_A0579	HI9a	CD9	579	GAGTCACCAATCTGC
ADT_A0581	3C10	TCR V alpha7.2	581	TACGAGCAGTATTCA
ADT_A0583	B3	TCR Vg9	583	AAGTGATGGTATCTG
ADT_A0576	9F10	CD49d	576	CCATTCAACTTCCGG
ADT_A0572	1D9-M12	C5L2	572	ACAATTTGTCTGCGA
ADT_A0574	HI264	CD235a	574	AGAGTATGTATGGGA
ADT_A0569	5D3	CD338 (ABCG2)	569	TAAGACTTGGCCGTC
ADT_A0577	AD2	CD73	577	CAGTTCCTCAGTTCG
ADT_A0578	HM47	CD79a	578	CTTATCACCCGCTTT
ADT_A0598	S16017E	CD110	598	TGTTGTAAGATGCCA
ADT_A0597	9E9A8	CD209 (DC-SIGN)	597	TCACTGGACACTTAA
ADT_A0410	HB-7	CD38	410	CCTATTCCGATTCCG
ADT_A0447	OX-104	CD200	447	CACGTAGACCTTTGC
ADT_A0592	DX27	CD158b (KIR2DL2/L3, NKAT2)	592	GACCCGTAGTTTGAT
ADT_A0805	TX25	CD226 (DNAM-1)	805	AGACCAACTCATTCA
ADT_A0804	K041E5	CD186	804	GACAGTCGATGCAAC
ADT_A0600	UP-R1	CD158f	600	AAAGTGATGCCACTG
ADT_A0599	DX9	CD158e1 (KIR3DL1, NKB1)	599	GGACGCTTTCCTTGA
ADT_A0364	WM15	CD13	364	TTTCAACGCCCTTTC
ADT_A0586	TREM-26	CD354	586	TAGCCGTTTCCTTTG
ADT_A0582	B6	TCR Vd2	582	TCAGTCAGATGGTAT
ADT_A0588	33.1 (Ab33)	CD202b (Tie2/Tek)	588	CGATCCCTTACCTAT
ADT_A0175	NK92.39	CD96	175	TGGCCTATAAATGGT
ADT_A0420	HP-MA4	CD158	420	TATCAACCAACGCTT
ADT_A0801	P30-15	CD337 (NKp30)	801	AAAGTCACTCTGCCG
ADT_A0820	2E1B02	CD130	820	CACGAGAATTTCAGT
ADT_A0822	NY2	CD142	822	CACTGCCGTCGATTA
ADT_A0829	509f6	CD307	829	TCACGCAGTCCTCAA
ADT_A0830	162.1	CD319 (CRACC)	830	AGTATGCCATGTCTT
ADT_A0821	67D2	CD164	821	GAGGCACTTAACATA
ADT_A0814	HD30	CD205 (DEC-205)	814	CTATCGTTTGATGCA
ADT_A0026	CC2C6	CD47	26	GCATTCTGTCACCTA
ADT_A0590	NKTA255	CD305	590	ATTTCCATTCCCTGT
ADT_A0433	8C11	CD325	433	CCTTCCCTTTCCTCT
ADT_A0819	UV4	CD126	819	TGATGGGAGCTTATC
ADT_A0817	W7C5	CD109	817	CACTTAACTCTGGGT
ADT_A0591	15C4	LOX-1	591	ACCCTTTACCGAATA
ADT_A0853	50C1	CD371 (CLEC12A)	853	CATTAGAGTCTGCCA
ADT_A0845	3B2/TA8	CD99	845	ACCCGTCCCTAAGAA
ADT_A0060	5E10	CD90 (Thy1)	60	GCATTGTACGATTCA
ADT_A0844	MEM-55	CD45RB	844	AGATGGGACTCACCA
ADT_A0858	TRA-2-10	CD46	858	ACAGTACGACCTTCT

ADT_A0866	AYP1	CLEC1B	866	TGCCAGTATCACGTA
ADT_A0008	24F.10C12	CD273	8	TCAACGCTTGGCTAG
ADT_A0170	MIH26	BTLA	170	GTTATTGGACTAAGG
ADT_A0138	UCHT2	CD5	138	CATTAACGGGATGCC
ADT_A0154	O323	CD27	154	GCACTCCTGCATGTA
ADT_A0153	SA231A2	KLRG1	153	CTTATTTCCTGCCCT
ADT_A0931	1C1	CD131	931	CTGCATGAGACCAAA
ADT_A0920	ASL-24	CD82	920	TCCCACTTCCGCTTT
ADT_A0934	HSL96	CD179a	934	TAGATGGGATTCCGG
ADT_A0935	LN2	CD74	935	CTGTAGCATTTCCCT
ADT_A0932	31G4D8	Lymphotoxin b Receptor (LT-bR)	932	CCTCTATTCAGAGCA

Table S8. List of antibodies used for mass cytometry.

Antigen	Conjugate	Clone	Company	Cat #
CD2	Biotin	RPA-2.10	eBioscience	13-0029-82
CD3	Biotin	OKT3	eBioscience	13-0037-82
CD4	Biotin	RPA-T4	eBioscience	13-0049-82
CD7	Biotin	eBio124-1D1 (124-1D1)	eBioscience	13-0079-82
CD8a	Biotin	HIT8a	eBioscience	13-0089-82
CD10	Biotin	eBioCB-CALLA (CB-CALLA)	eBioscience	13-0106-82
CD11b	Biotin	ICRF44	eBioscience	13-0118-82
CD14	Biotin	61D3	eBioscience	13-0149-82
CD19	Biotin	HIB19	eBioscience	13-0199-82
CD20	Biotin	2H7	eBioscience	13-0209-82
CD56	Biotin	CMSSB	eBioscience	13-0567-82
CD41	89Y	HIP8	Fluidigm	3089004B
CD45	141Pr	HI30	Fluidigm	3141009B
p-AMPKa (Thr172)	142Nd	40H9	CST	2535S
CD123	143Nd	6H6	Fluidigm	3143014B
CD38	144Nd	HIT2	Fluidigm	3144014B
p53	145Nd	X77	abcam	ab16465
SOCS3	146Nd	516919	R&D	MAB5696
p-Stat5 [Y694]	147Sm	47	Fluidigm	3147012A
CD110 (C-MPL)	148Nd	1.78.1	BD	562137
CD34	149Sm	581	Fluidigm	3149013B
HIF-1α	150Nd	H-206	Santa Cruz	sc-10790
GRIM19	151Eu	6E1BH7	Abcam	ab110240
p-Akt [S473]	152Sm	D9E	Fluidigm	3152005A
p-Stat1 [Y701]	153Eu	58D6	Fluidigm	3153003A
Nrf2 [EP1808Y]	154Sm	EP1808Y	Abcam	ab62352
CD45RA	155Gd	HI100	Fluidigm	3155011B
p38 [T180/Y182]	156Gd	D3F9	Fluidigm	3156002A
p-Stat3 [Y705]	158Gd	4/P-Stat3	Fluidigm	3158005A
p-MAPKAPK2 [T334]	159Tb	27B7	Fluidigm	3159010A
Nrf1	160Gd	EPR5554(N)	Abcam	ab175932
p-Jak2 (Tyr1008)	161Dy	D4A8	CST	8082S
Ki-67	162Dy	B56	Fluidigm	3162012B
mTOR (phospho S2448)	163Dy	EPR426(2)	Abcam	ab109268
LKB1 (phospho S428)	164Dy	polyclonal	Abcam	ab63473
pNF-kB p65 [S529]	166Er	K10895.12.50	Fluidigm	3166006A
Hepcidine	167Er	1F9	Abnova	H00057817-M02
CD71	168Er	CY1G4	biolegend	334102
vWF	169Tm	polyclonal	Abcam	ab11713
Biotin	170Er	1D4C5	Fluidigm	3170003B
pERK1/2 [T202/Y204]	171Yb	D13.14.4E	Fluidigm	3171010A
Anti-Cleaved Caspase 3	172Yb	5A1E	Fluidigm	3172023A
CD90	173Yb	5.00E+10	Fluidigm	3173011B
PF4	174Yb	170138	R&D	MAB7952
PGC-1	175Lu	polyclonal	Merck	AB3242

Table S9. Numbers of cells submitted for scRNA-seq, and after first steps of quality control prior to further downstream bioinformatics analyses.

		10x scRNA-seq and CITE-seq of CD19^{-}CD34^{+} HSCPs			Smart-seq2 of phenotypic HSC/MPPs		
**Donor**	**Tissue**	**Cells submitted**	**Cells recovered**	**Recovery [%]**	**Cells submitted**	**Cells passing QC prior to calculation of identity scores**	**Number of cells passing QC/removal outliers prior DSeq2**
OD1	BM	15,032	5,753	38.27	190	129	104
OD1	PB	7,516	2,607	34.69	-	-	-
OD1	SPL	15,032	5,295	35.22	190	165	150
OD2	BM	17,500	6,216	35.52	272	242	232
OD2	PB	17,500	6,528	37.30	91	87	NA
OD2	SPL	15,011	5,026	33.48	92	74	60
OD3	BM	25,000	12,590	50.36	-	-	-
OD4	BM	30,000	15,364	51.21	-	-	-
OD4	SPL	30,000	14,264	47.55	-	-	-
LD1	PB	12,471	5,174	41.49	-	-	-
LD2	PB	17,500	5,921	33.83	-	-	-
LD3	PB	17,500	5,439	31.08	-	-	-
LD4	PB	17,500	7,325	41.86	-	-	-
LD5	PB	30,000	13,838	46.13	-	-	-
LD6	PB	30,000	18,025	60.08	-	-	-
mPB1	mPB	15,000	8,220	54.80	-	-	-
mPB2	mPB	15,000	7,038	46.92	-	-	-
mPB3	mPB	15,000	5,982	39.88	-	-	-
mPB4	mPB	15,000	6,786	45.24	-	-	-
HS SPL 1	SPL	15,000	5,034	33.56	-	-	-
HS SPL 2	SPL	15,000	4,905	32.70	-	-	-

SUPPLEMENTAL FIGURES

Figure S1

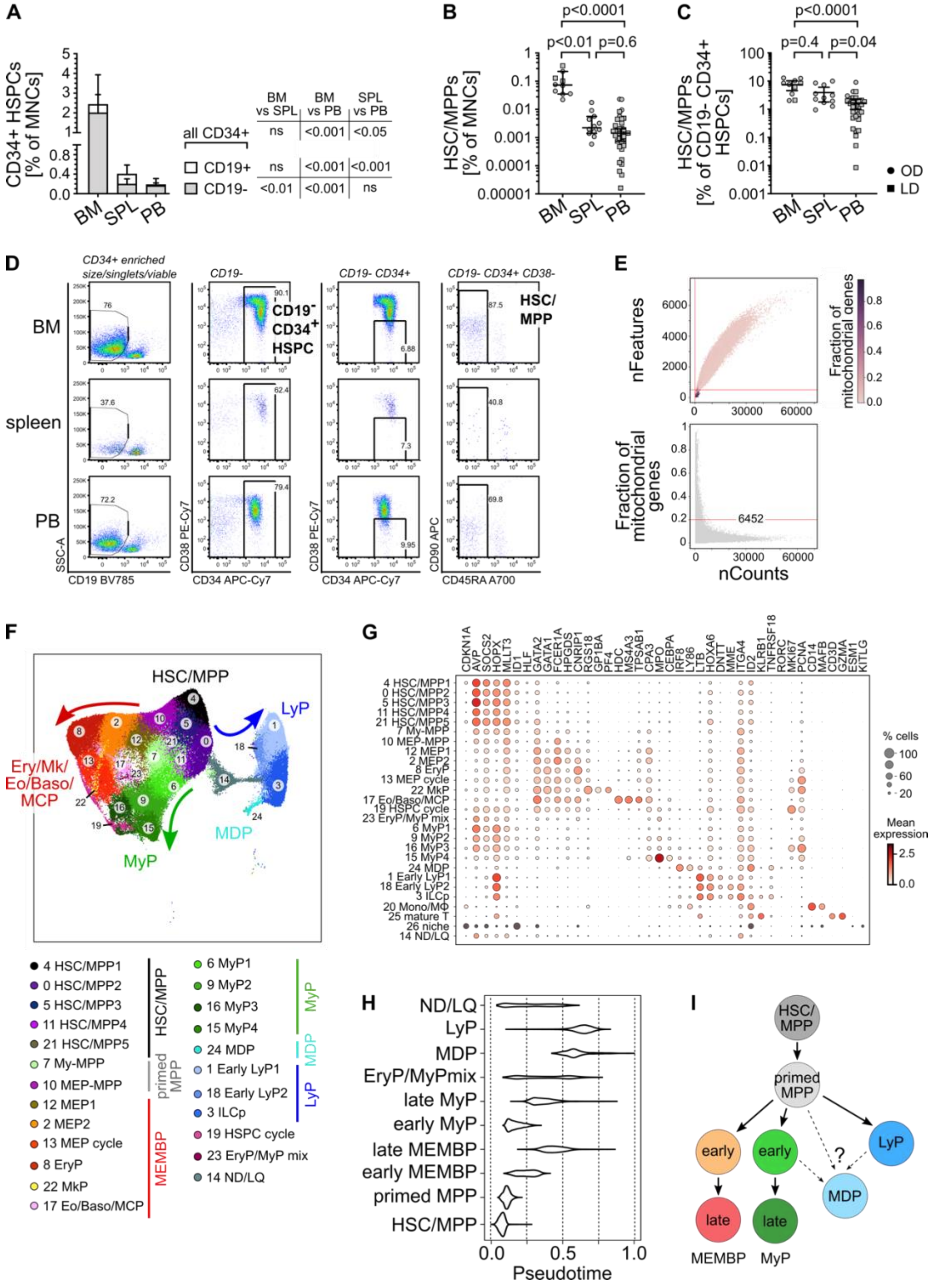

Figure S1. The adult human HSPC landscape across BM, PB and spleen. Related to Figure 1. **(A)** Stacked bar graph showing the frequencies of CD19^{-}CD34^{+} and CD19^{+}CD34^{+} HSPCs in BM (n=9), PB (n=31), spleen (n=10) over 29 experiments.

Mean ± SD is shown. **(B-C)** Frequencies of phenotypic HSC/MPPs ($CD19^-CD34^+CD38^-CD45RA^-$) in mononuclear cells (MNCs **(B)** and $CD19^-CD34^+$ HSPCs **(C)** from BM (n=10), PB (n=30) and spleen (n=11) over 29 experiments. Median ± 95% confidence is shown. **(D)** Gating strategy for the purification of $CD19^-CD34^+$ HSPCs and $CD19^-CD34^+CD38^-CD45RA^-$ HSC/MPPs from all three tissues. Representative plots from OD1 are shown. **(E)** Feature plots of 10x Genomics scRNA-seq data combining BM, spleen and non-mobilized PB. Red lines indicate the thresholds used for the cut-off of nFeatures, nCounts and mitochondrial gene content. 6,452 cells were removed due to a mitochondrial gene content > 20%. **(F)** UMAP of the multisite HSPC landscape after exclusion of mature cells (see methods). Colors and numbers indicate clusters of transcriptionally distinct cells as defined by Leiden clustering. Clusters were annotated using known lineage and stem cell marker genes found amongst the most differentially expressed genes in each cluster (Table S2a). ND/LQ: cluster of low quality for which identity could not be defined using known marker genes. HSC/MPP: hematopoietic stem cell/multipotent progenitor; MEP: Megakaryocyte-erythroid progenitor; EryP: erythroid progenitor; MkP: megakaryocytic progenitor; Eo/Baso/MCP: eosinophil/basophil/mast cell progenitor; MEMBP: Megakaryocyte/erythroid/eosinophil/mast cell/basophil progenitor; MyP: myeloid progenitor; MDP: monocyte/dendritic cell progenitor; LyP: lymphoid progenitor; ILCp: Innate lymphoid cell progenitor. **(G)** Expression values of selected marker genes for all Leiden clusters (as in F above) in the HSPC landscape combining all donors and all tissues. Circle color shows mean scaled expression values and circle size represents the proportion of expressing cells per cluster. **(H)** Pseudotime was assigned to each cell of the HSPC landscape choosing a cell in cluster 4 (HSC/MPP1) as the root. Violin plots of the pseudotime value for each HSPC group is shown. HSPC groups were defined as shown in Figure 1A and Table S2c. **(I)** Schematic differentiation hierarchy of the HSPC group as defined in Table S2c. A-C) Kruskal-Wallis; Dunn's multiple comparison test. LD; living donor, OD; organ donor, SPL; spleen.

Figure S2

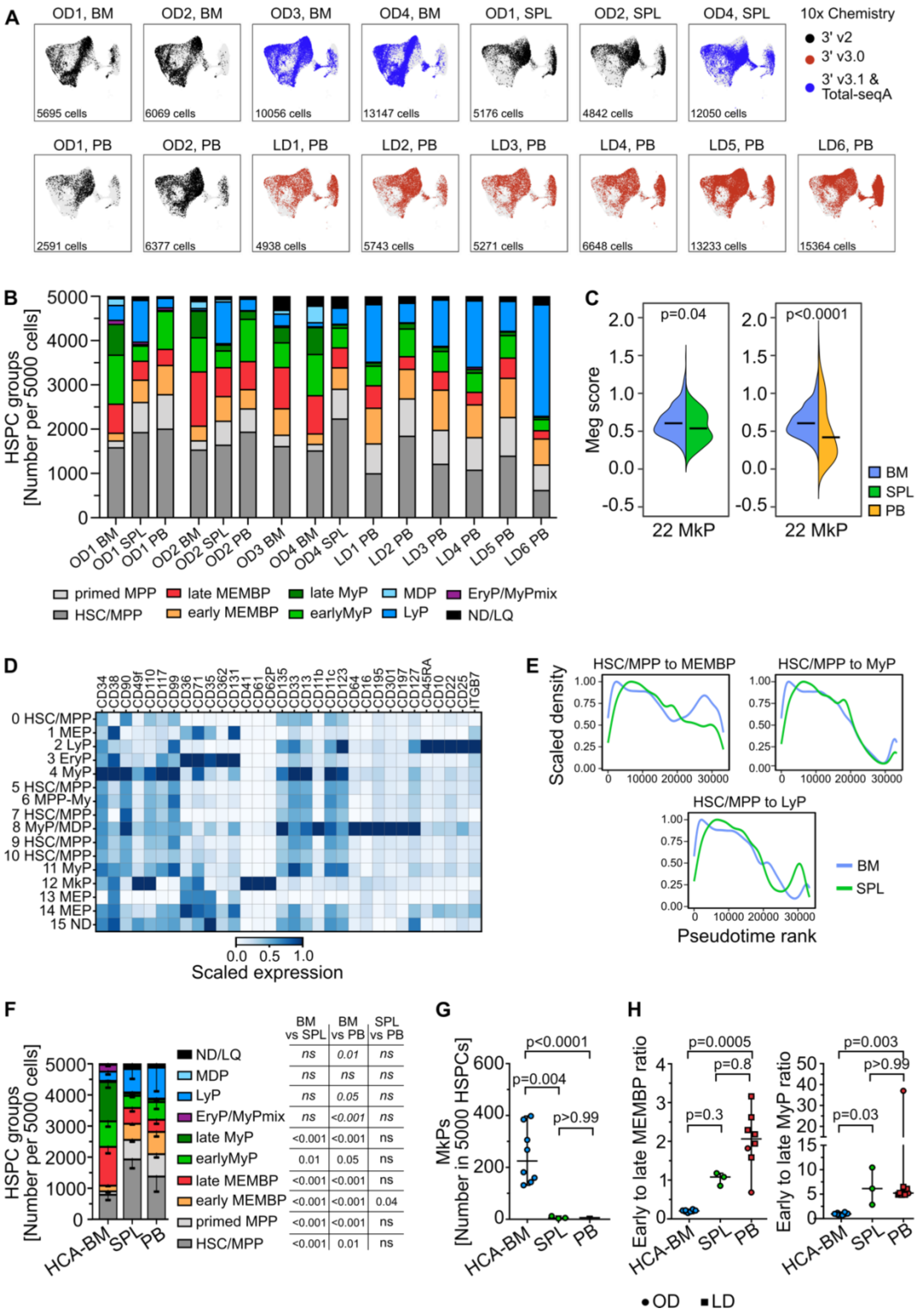

	BM vs SPL	BM vs PB	SPL vs PB
ND/LQ	ns	0.01	ns
MDP	ns	ns	ns
LyP	ns	0.05	ns
EryP/MyPmix	ns	<0.001	ns
late MyP	<0.001	<0.001	ns
earlyMyP	0.01	0.05	ns
late MEMBP	<0.001	<0.001	ns
early MEMBP	<0.001	<0.001	0.04
primed MPP	<0.001	<0.001	ns
HSC/MPP	<0.001	0.01	ns

Figure S2. Changes in HSPC composition between BM, PB and spleen. Related to Figure 2. **(A-G)** Analysis of 10x Genomics scRNA-seq and CITE-seq data combining all donors but comparing different tissues. **(A)** UMAPs of the HSPC landscape for each individual donor and tissue, colored by the version of 10x reagents used for scRNA-

seq and CITE-seq library preparation. The number of single cells from each donor and tissue is indicated in each plot. **(B)** Bar graphs of the relative proportion of each HSPC group in each individual donor (same data as in Figure 2B). **(C)** Split violin plots of the Meg-lineage priming scores in cluster 22 (MkPs) comparing BM with spleen or PB. Median is shown (solid line). 38≤ single cells ≥ 491. Mann-Whitney test. **(D)** Heatmap of cell surface protein expression for each Leiden cluster using exclusively the CITE-Seq protein data from two BMs (OD3: 9,477 cells, OD4: 12,500 cells) and one spleen (OD4: 11,822 cells). **(E)** Pseudotime rank of CITE-seq protein data for each lineage branch (starting with HSC/MPPs) comparing BM and spleen. **(F-H)** Comparison of spleen and PB from our multi-tissue landscape to the benchmark Human Cell Atlas (BM) BM reference [31]. **(F)** Bar graph of the relative composition of HSPC groups in HCA-BM, PB and spleen. Mean ± SD is shown. **(G)** Relative number of MkPs (cluster 22) in each tissue. **(H)** The ratio of early to late progenitors of the MEMB (left) or My (right) branch is shown. F,G) One-way ANOVA with post hoc Tukey test, except for LyP, EryP/MyPmix and ND/LQ clusters in (F) (not normally distributed, italic letters), for which a Kruskal-Wallis test with Dunn's multiple comparison was used. ns; p>0.05. H) Kruskal-Wallis test with Dunn's multiple comparison. G,H) Median ± 95% confidence is shown. LD; living donor, OD; organ donor, SPL; spleen.

Figure S3

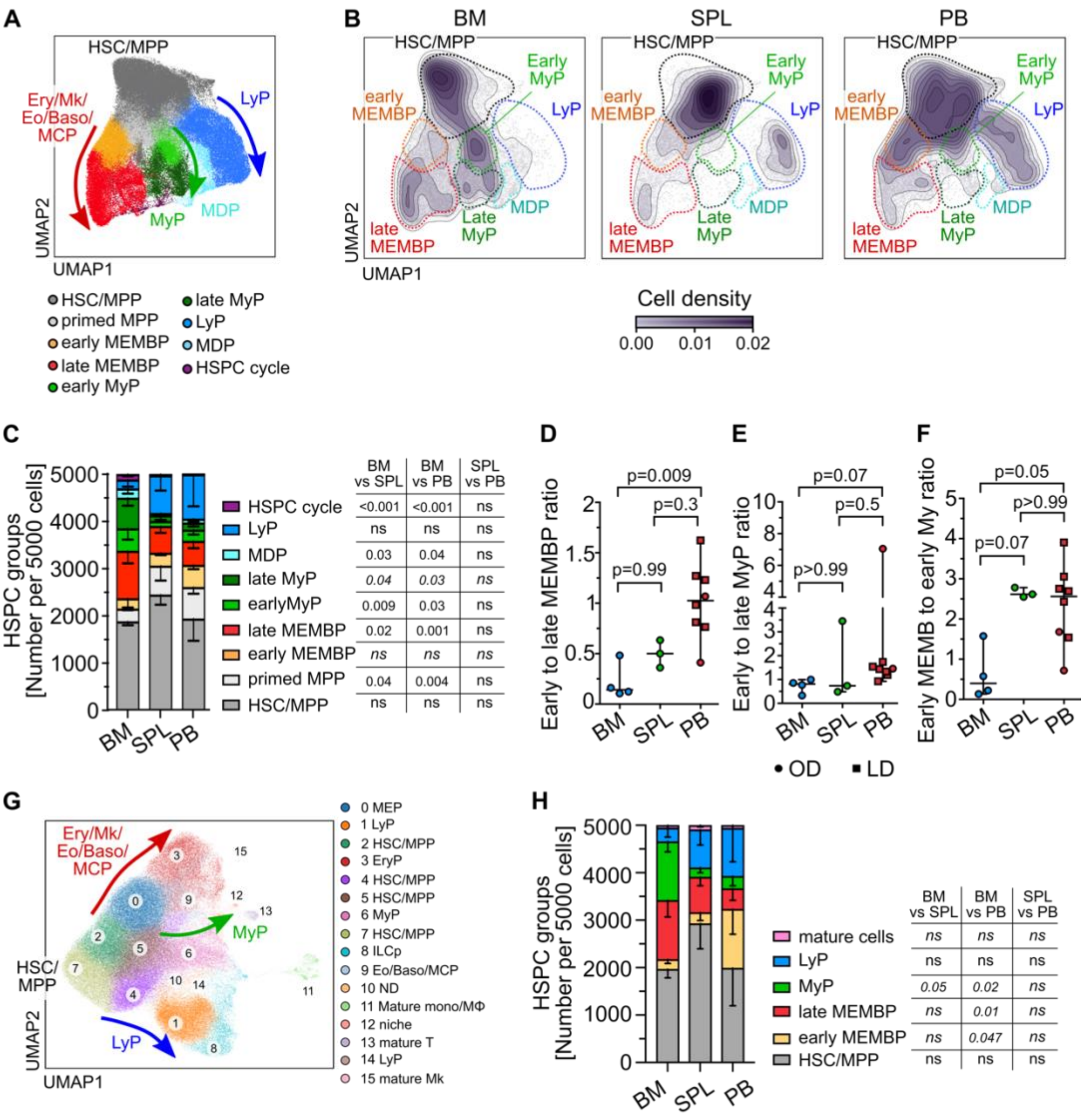

Figure S3. Analysis of BM, spleen and PB scRNA-seq data using cell cycle regression and ComBat batch correction. Related to Figure 2. **(A-F)** Analysis of 10x Genomics scRNA-seq data (same as in Figure 2) after regression of cell cycle genes. **(A)** UMAP of the multisite HSPC landscape. Clusters with similar cell identity are shown as HSPC groups, as indicated by cluster colors. **(B)** Density of cells across the UMAP coordinates of each tissue, displayed as contours filled by a color gradient. **(C)** Bar graph of the relative composition of HSPC groups in BM, spleen and PB. **(D,E)** The ratio of early to late progenitors of the MEMB **(D)** or My **(E)** branch is shown. **(F)** Ratio of early MEMBP to early MyP clusters. **(G-H)** ComBat batch correction was used as an alternative method to Seurat to integrate the 10x datasets of all donors, combining all tissues. **(G)** Annotated UMAP of the HSPC landscape after ComBat correction. Colors and numbers indicate clusters of transcriptionally distinct cells as partitioned by Leiden clustering. **(H)** HSPC group composition after ComBat correction

comparing BM, spleen and PB. C,H) Mean ± SD is shown. One-way ANOVA with post hoc Tukey test, except for not normally distributed clusters (indicated by italic text), for which a Kruskal-Wallis test with Dunn's multiple comparison was used. ns; $p>0.05$. D,E,F) Median ± 95% confidence is shown. Kruskal-Wallis; Dunn's multiple comparison test. LD: living donor, OD: organ donor, SPL: spleen.

Figure S4

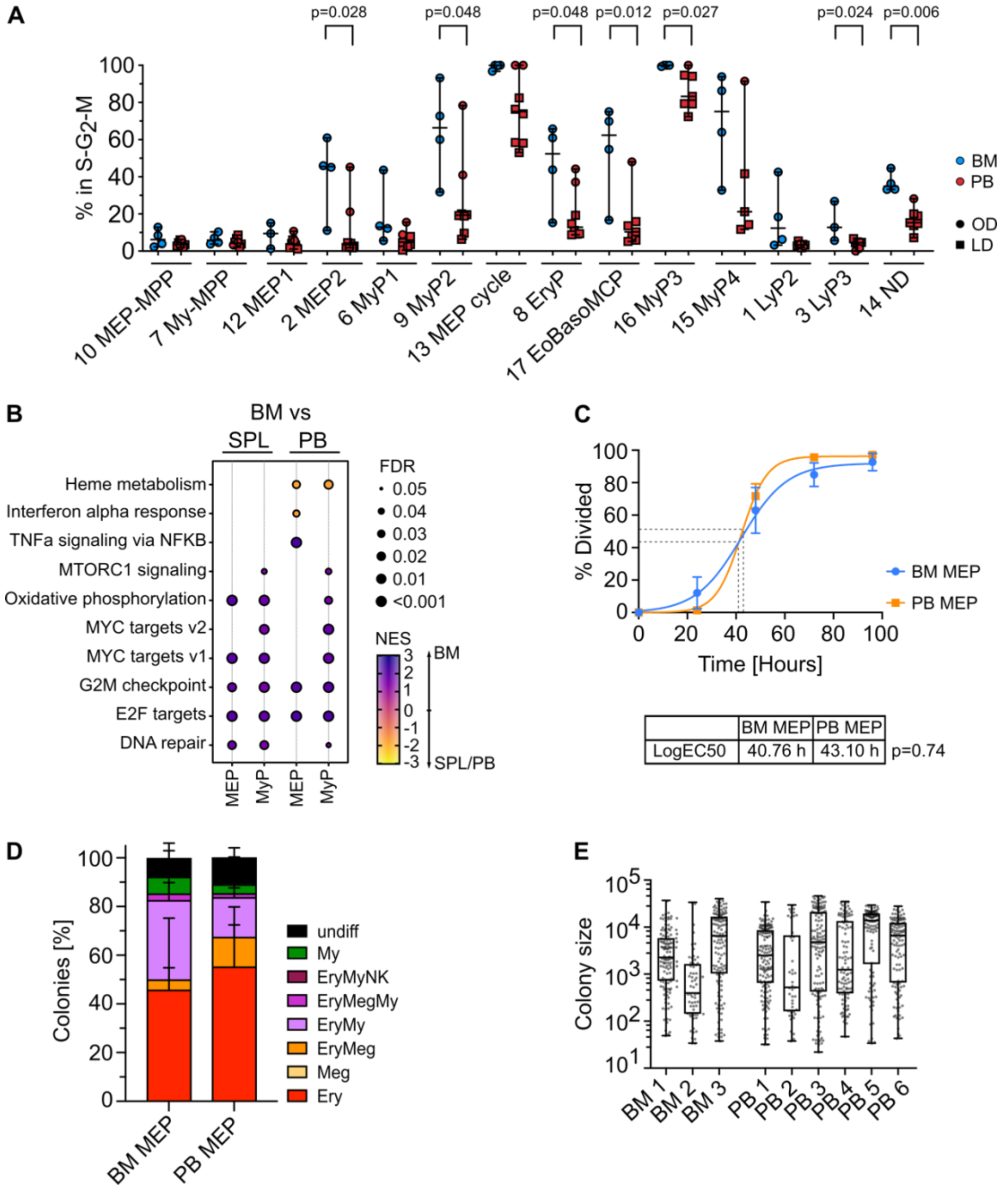

Figure S4. Low to no cycling activity of HSPCs in extramedullary sites. Related to Figure 3. **(A)** Percentage of cells in S-G$_2$-M phase (assigned by cell cycle phase scoring as described by [11]) for each progenitor cluster comparing unmatched BM and PB. Median ± 95% confidence is shown. LD; living donor, OD; organ donor. Two-tailed Mann-Whitney test. **(B)** Selected pathways (MSigDB hallmark) enriched in early progenitors of the MEMB (MEP: cluster 2,10,12) and My branch (MyP: cluster 6,7,9) comparing BM with spleen or BM with PB. All enriched pathways are summarized in Table S4b-e. **(C-E)** Single sorter purified CD19$^-$CD34$^+$CD38$^+$CD123$^-$CD45RA$^-$ MEPs

from BM (n=317 single cells from 3 donors) and PB (n=655 single cells from 6 donors) were cultured for 2 weeks in medium promoting My, Ly, Ery and Meg differentiation. Five independent experiments were performed. **(C)** Time to first division kinetics (counted for the first 4 days, excluding dead cells) comparing BM MEPs as PB MEPs. Curve is least-squares sigmoid fit. Estimated time to first division (logEC50) is indicated by dashed gray lines, p is by extra sum of squares F-test. **(D)** Colony composition after 2 weeks culture of BM MEPs and PB MEPs. Mean ± SD is shown. All not significant with two-tailed Mann-Whitney test. **(E)** Box plots of colony size for each sample (box represents median and interquartile range). LD; living donor, OD; organ donor, SPL: spleen.

Figure S5

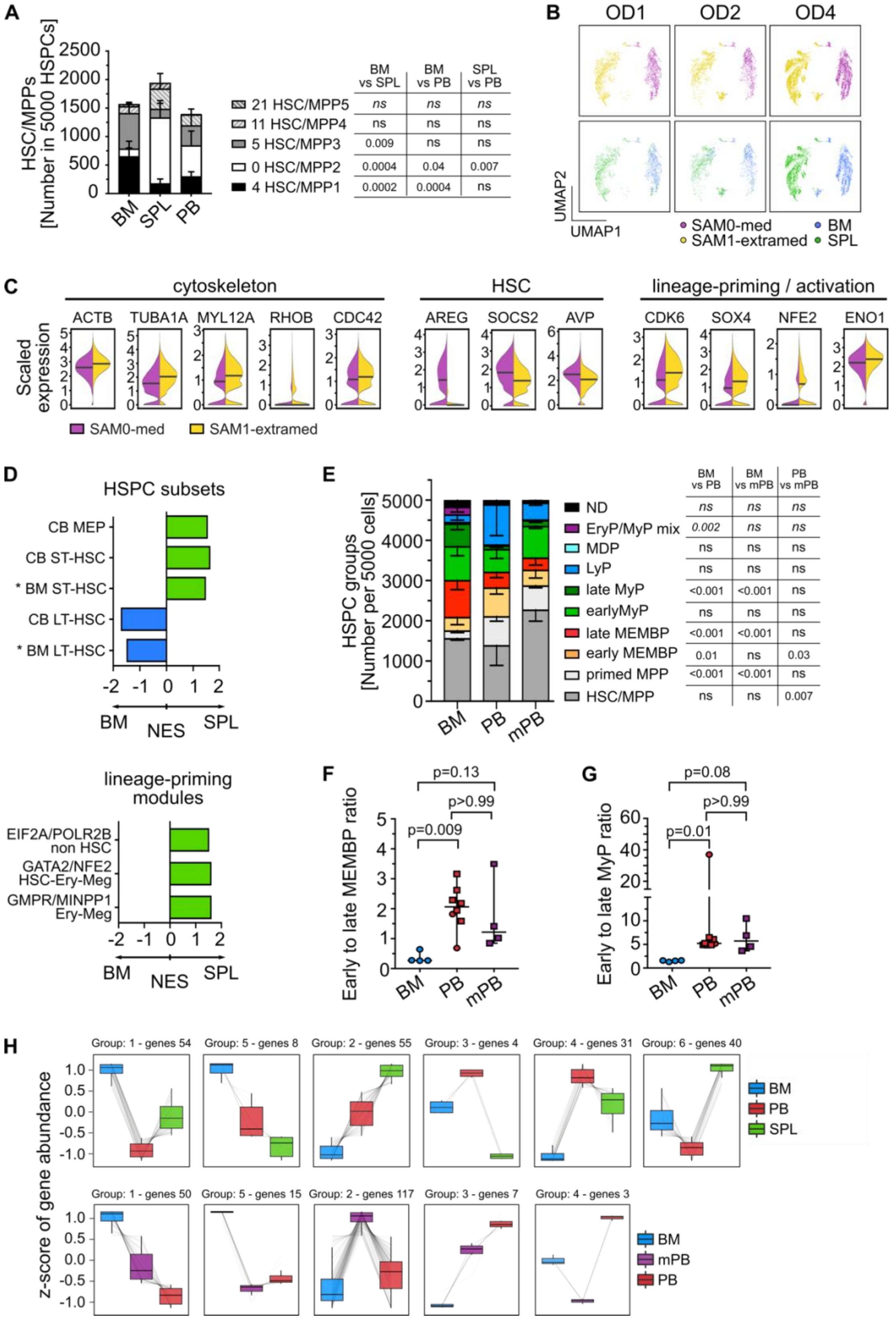

Figure S5. Comparison of HSC/MPPs from BM and extramedullary hematopoietic tissues. Related to Figure 4. **(A)** Composition of HSC/MPP clusters

comparing different tissues. **(B)** UMAPs colored by SAM clusters and tissue for each donor. **(C)** Split violin plots showing scaled expression values of selected genes related to cytoskeleton, HSC and lineage-priming genes differentially expressed (FDR<0.05) between SAM0-med and SAM1-extramed clusters. Line indicates median. **(D)** Phenotypic HSC/MPPs (CD19$^-$CD34$^+$CD38$^-$CD45RA$^-$) from BM and spleen of OD1 and OD2 were single-cell sorted and transcriptome was sequenced using the Smart-seq2 protocol. Bar graphs show all population-specific signatures (left, [26,28], signature for BM HSCs unpublished) and lineage-priming modules (right, [29]) from pre-ranked GSEA analysis (FDR <0.05) comparing BM and spleen HSC/MPPs. **(E-G)** Analysis of scRNA-seq data integrating BMs and non-mobilized PBs (same as in Figure 2) with four mobilized PB (mPB) CD19$^-$CD34$^+$ HSPCs (28,026 cells). **(E)** Relative composition of HSPC groups comparing BM, non-mobilized PB and mPB. **(F,G)** The ratio of early to late progenitors of the MEMB (F) or My (G) branch is shown. Median ± 95% confidence is shown. Kruskal-Wallis test with Dunn's multiple comparison. **(H)** Expression patterns for the key medullary and extramedullary signature genes comparing BM, non-mobilized PB and spleen (top panel) or BM, non-mobilized PB and mobilized PB (bottom panel). A,E) Mean ± SD is shown. One-way ANOVA with post hoc Tukey test, except for not normally distributed clusters (indicated in italic), for which a Kruskal-Wallis test with Dunn's multiple comparison was used. ns; p>0.05. SPL: spleen.

Figure S6

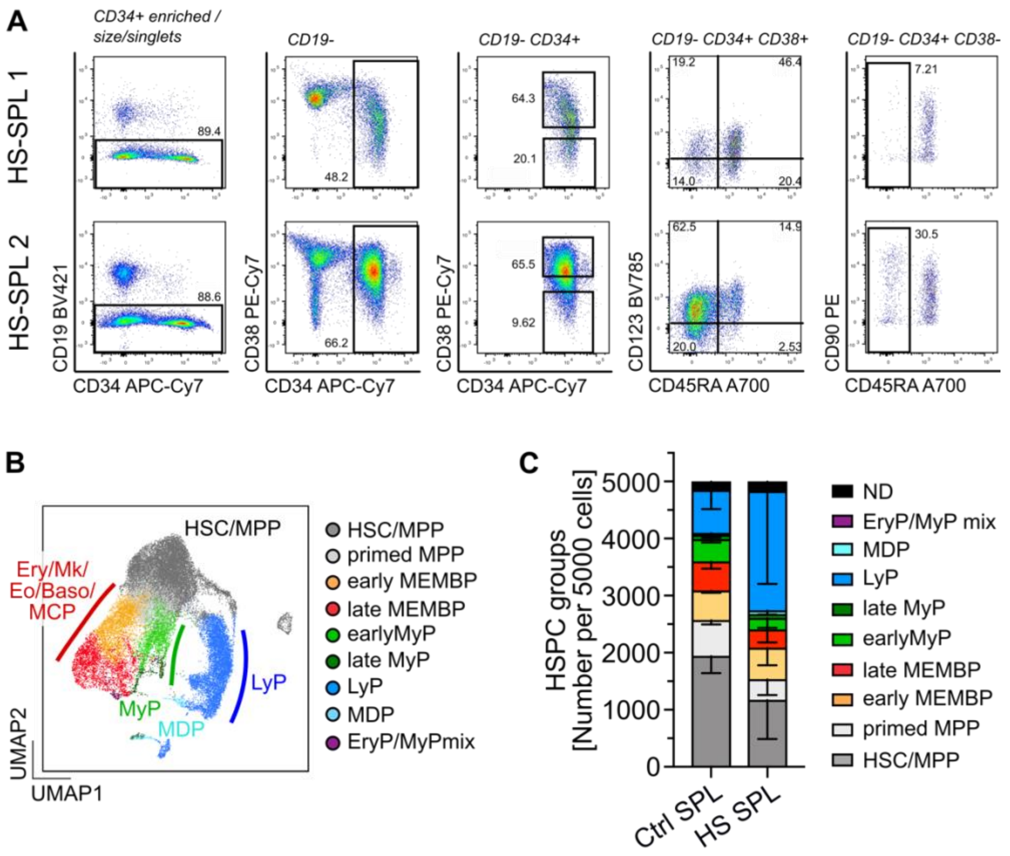

Figure S6: scRNA-seq analysis of hereditary spherocytosis spleens. Related to Figure 5. **(A)** Flow cytometry data from HSPC phenotyping of both hereditary spherocytosis (HS) spleen $CD34^+$ enriched samples. **(B)** UMAP after integration of 10x Genomics scRNA-seq data from two HS spleens (n=9,939 cells) with control spleens (same as in Figure 2). HSPC groups are indicated by different cluster colors. **(C)** HSPC group composition in control and HS spleens. Mean ± SD is shown. SPL: spleen.

Figure S7

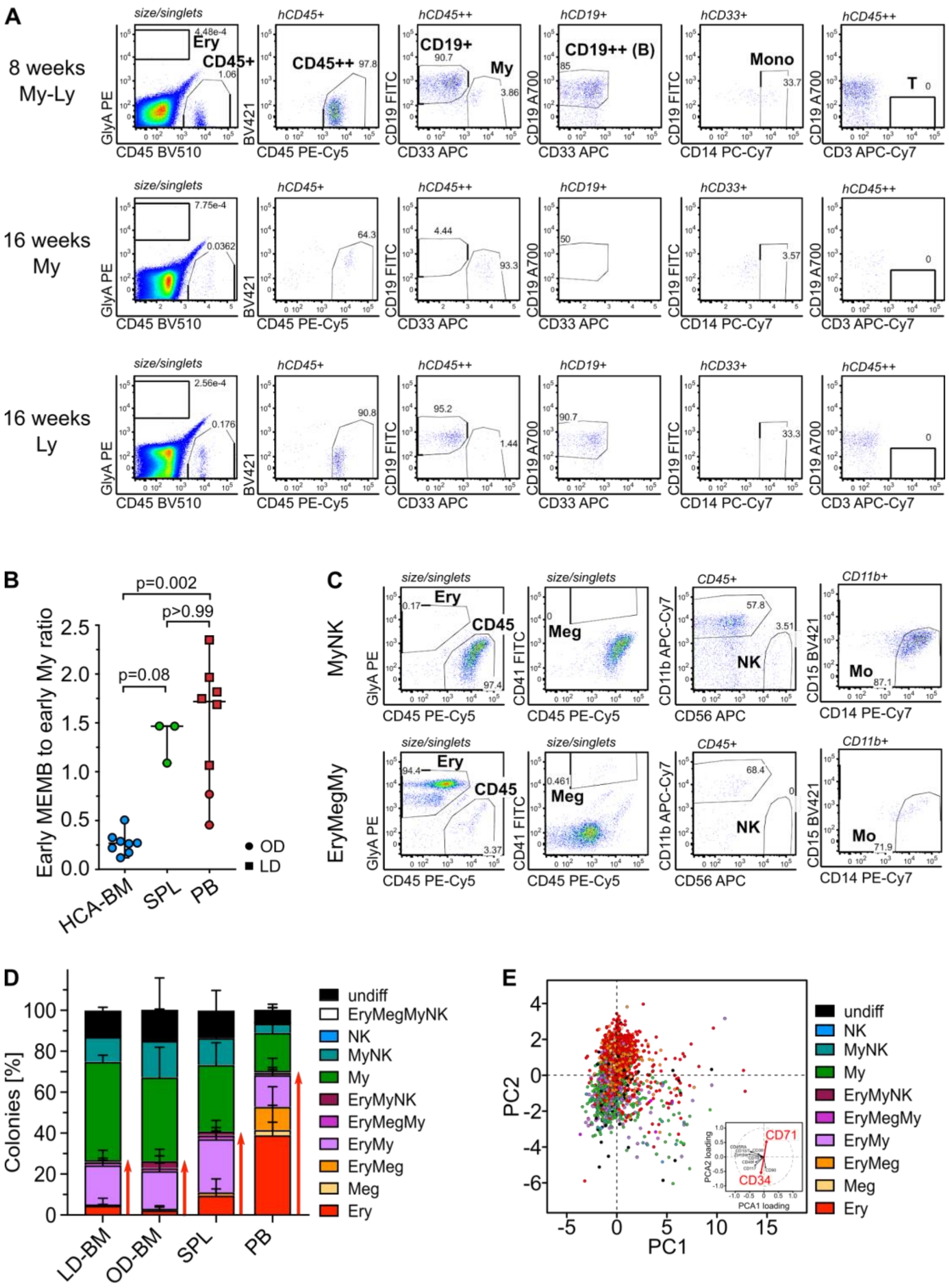

Figure S7. Functional characterization of the phenotypic HSC/MPP pool from steady-state PB. Related to Figure 6. **(A)** Representative examples of flow cytometry plots of human cell engraftment in NSG mice after 8 and 16 weeks of repopulation with HSC/MPPs from PB (My-Ly: Myelo-lymphoid; Ly: lymphoid; My: myeloid engraftment) using antibody panel (K). Within engrafted human leukocytes (CD45^{++}, doubly labeled

by both hCD45 BV510 and hCD45 PE-Cy5 antibodies), $CD19^{++}$ B cells (B), $CD33^{+}$ myeloid cells (My), $CD33^{+}CD14^{+}$ monocytes (Mono) and $CD3^{+}$ T cells (T) were distinguished. Erythrocytes (Ery) were identified by GlyA expression. This gating strategy was used for Figure 6A. **(B)** Ratio of early MEMB (sum cluster 2,12) to early My (sum cluster 6,9) progenitors in spleen and PB compared to the HCA BM reference [31]. Median ± 95% confidence interval is shown. Kruskal-Wallis; Dunn's multiple comparison test. **(C)** Two representative examples of the gating strategy used to determine lineage composition of colonies derived from single cells grown in medium supporting Ery, Meg, My and Ly differentiation. Colonies were defined as described in the methods. **(D)** Bar graphs show average colony composition for each tissue assessed by flow cytometry. Same data as in Figure 6C. BM from organ donors (OD-BM, n=496 single cells from 4 samples) and living donors (LD-BM, n=417 single cells from 3 samples) are shown separately. Red bar highlights all colonies containing Ery and/or Meg colonies as also quantified in Figure 6C. **(E)** Phenotypic HSC/MPPs from PB (n=2,164; 20 independent PBs) were index-sorted, recording the cell surface expression of known hematopoietic stem/progenitor markers for each (CD19, CD34, CD38, CD45RA, CD90, CD117, CD71, CD49f, CD10/CD7), and seeded into medium promoting simultaneous differentiation into myeloid (My), erythroid (Ery), megakaryocytic (Meg) and lymphoid (Ly, measured by growth of NK cell colonies) lineages [2]. Plot shows the principal component analysis (PCA) of the surface markers expression on phenotypic PB HSC/MPPs at the time of sort. Colors indicate the colony type produced by each single HSC/MPP. PCA loadings are shown in the lower right corner. LD: living donor, OD: organ donor, SPL: spleen.

Figure S8

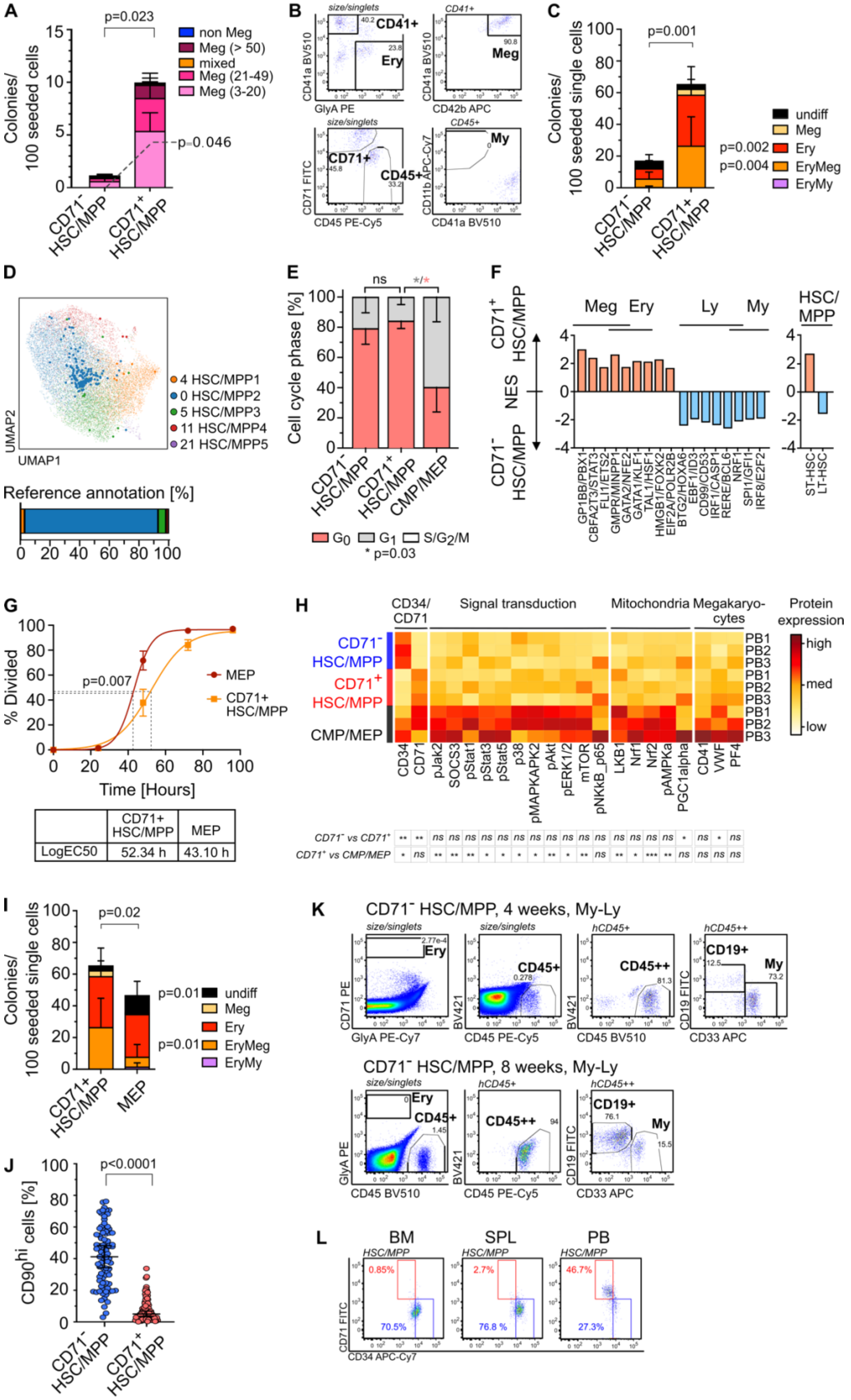
A
p=0.023
p=0.046
Colonies/ 100 seeded cells
non Meg
Meg (> 50)
mixed
Meg (21-49)
Meg (3-20)
CD71- HSC/MPP
CD71+ HSC/MPP
B
size/singlets
CD41+
Ery
Meg
CD41a BV510
GlyA PE
CD42b APC
CD71+
CD45+
CD71 FITC
CD45 PE-Cy5
My
CD11b APC-Cy7
CD41a BV510
C
p=0.001
Colonies/ 100 seeded single cells
undiff
Meg
Ery
EryMeg
EryMy
p=0.002
p=0.004
D
4 HSC/MPP1
0 HSC/MPP2
5 HSC/MPP3
11 HSC/MPP4
21 HSC/MPP5
UMAP1
UMAP2
Reference annotation [%]
E
ns
/
Cell cycle phase [%]
CMP/MEP
G0
G1
S/G2/M
* p=0.03
F
Meg
Ery
Ly
My
HSC/ MPP
NES
GP1BB/PBX1
CBFA2T3/STAT3
FLI1/ETS2
GMPR/MINPP1
GATA2/NFE2
GATA1/KLF1
TAL1/HSF1
HMGB1/FOXK2
EIF2A/POLR2B
BTG2/HOXA6
EBF1/ID3
CD99/CD53
IRF1/CASP1
RERE/BCL6
NRF1
SPI1/GFI1
IRF8/E2F2
ST-HSC
LT-HSC
G
% Divided
p=0.007
MEP
CD71+ HSC/MPP
Time [Hours]
LogEC50
CD71+ HSC/MPP 52.34 h
MEP 43.10 h
H
CD34/ CD71
Signal transduction
Mitochondria
Megakaryo- cytes
Protein expression
high
med
low
CD71- HSC/MPP
CD71+ HSC/MPP
CMP/MEP
PB1
PB2
PB3
CD34
CD71
pJak2
SOCS3
pStat1
pStat3
pStat5
p38
pMAPKAPK2
pAkt
pERK1/2
mTOR
pNKkB_p65
LKB1
Nrf1
Nrf2
pAMPKa
PGC1alpha
CD41
VWF
PF4
CD71- vs CD71+
CD71+ vs CMP/MEP
I
p=0.02
p=0.01
Colonies/ 100 seeded single cells
undiff
Meg
Ery
EryMeg
EryMy
CD71+ HSC/MPP
MEP
J
p<0.0001
CD90hi cells [%]
CD71- HSC/MPP
CD71+ HSC/MPP
K
CD71- HSC/MPP, 4 weeks, My-Ly
size/singlets
hCD45+
hCD45++
Ery
CD45+
CD45++
CD19+
My
CD71 PE
GlyA PE-Cy7
BV421
CD45 PE-Cy5
CD45 BV510
CD19 FITC
CD33 APC
CD71- HSC/MPP, 8 weeks, My-Ly
GlyA PE
L
BM
SPL
PB
HSC/MPP
0.85%
70.5%
2.7%
76.8 %
46.7%
27.3%
CD71 FITC
CD34 APC-Cy7

Figure S8. Characterization of CD71$^+$ HSC/MPPs. Related to Figure 6. **(A)** Percentage of Meg colonies from CD71$^-$ and CD71$^+$ HSC/MPPs in MegaCult^{TM} medium. n=3 PBs in 3 experiments. Two tailed paired t-test. (B-C) Single cell sorted CD71$^-$ (n=457 single cells; 5 independent PBs) and CD71$^+$ (n=1,005 single cells; 9 independent PBs) HSC/MPPs were grown in conditions optimized for Ery/Meg differentiation [39] over 6 experiments. **(B)** Representative examples for the gating strategy used to determine lineage composition of colonies. Colonies were defined as follows: Ery: CD45$^-$GlyA$^+$ >30 cells, Meg: GlyA$^-$CD41a$^+$CD42b$^+$ >20 cells, My: CD45$^+$CD11b$^+$ > 30 cells. **(C)** Colony types are shown as frequency of all seeded cells. Two-tailed Mann-Whitney test. **(D)** Smart-seq2 scRNA-seq data from CD71$^+$ PB HSC/MPPs was projected onto the HSC/MPP clusters of the 10x multitissue landscape. UMAP (left) shows the nearest neighbors for each single CD71$^+$ HSC/MPP (large dots) on the 10x HSC/MPP map (small dots). Bar graph shows the reference annotation for all CD71$^+$ HSC/MPPs in these projections. **(E)** Percentage PB CD71$^-$ HSC/MPPs, CD71$^+$ HSC/MPPs and CMP/MEPs in G_0 (Ki-67$^-$DAPI$^-$), G_1 (Ki-67$^+$DAPI$^-$) and S/G_2/M (Ki=67$^+$DAPI$^+$) cell cycle phases as determined by flow cytometry. n=6 PBs over 2 experiments. Wilcoxon test. **(F)** Significantly enriched (FDR <0.05 by pre-ranked GSEA and NES >1.6 or <-1.6) HSC/MPP-specific gene signatures from [28] (right panel) and lineage-priming gene signatures from [29] (left panel) in CD71$^-$ and CD71$^+$ HSC/MPPs. Enrichment for population-specific signatures from [26] are shown in Table S4I. **(G)** Time to first division kinetics (excluding dead cells) in pro-differentiating cultures comparing MEPs (same as in supplemental Figure 4C, n=655 single cells from 6 donors) and CD71$^+$ HSC/MPPs (n=373 single from PB. Curve is least-squares sigmoid fit. Estimated time to first division (logEC50) is indicated by dashed grey lines. p is by extra sum of squares F-test. **(H)** Heatmap shows scaled expression values of indicated proteins measured in CD71$^-$ HSC/MPPs, CD71$^+$ HSC/MPPs and CMP/MEPs of 3 independent PBs by mass cytometry. **(I)** Colony types produced by CD71$^+$ HSC/MPPs (n=1,005 single cells; 9 independent PBs, same as in supplemental Figure 8C) and MEPs (n=556 single cells; 6 independent PBs) in conditions optimized for Ery/Meg differentiation [39] over 6 experiments. Two-tailed Mann-Whitney test. **(J)** Frequency of CD90^{hi} cells in CD71$^-$ and CD71$^+$ PB HSC/MPPs. Median ± 95% confidence is shown. Paired two-tailed Wilcoxon test. **(K)** Representative examples of flow cytometry plots of human myelo-lymphoid (My-Ly) engraftment from CD71$^-$ PB HSC/MPPs in NSG mice at 4 and 8 weeks after transplantation (using antibody panel (M; 4 weeks and L; 8 weeks). Within engrafted human leukocytes (CD45^{++}, doubly labeled by both hCD45 BV510 and hCD45 PE-Cy5 antibodies), CD19$^+$ B cells and

$CD33^+$ myeloid (My) cells were distinguished. Erythrocytes (Ery) were defined by GlyA and/or CD71 expression. This gating strategy was used for Figure 6H-I. **(L)** Representative examples of flow cytometry plots showing the proportion of $CD71^-$ (blue gates) and $CD71^+$ (red gates) phenotypic HSC/MPPs ($CD19^-CD34^+CD38^-CD45RA^-$ cells) in BM and spleen. A,C,E,G,I) Mean ± SD is shown. SPL: spleen.

Figure S9

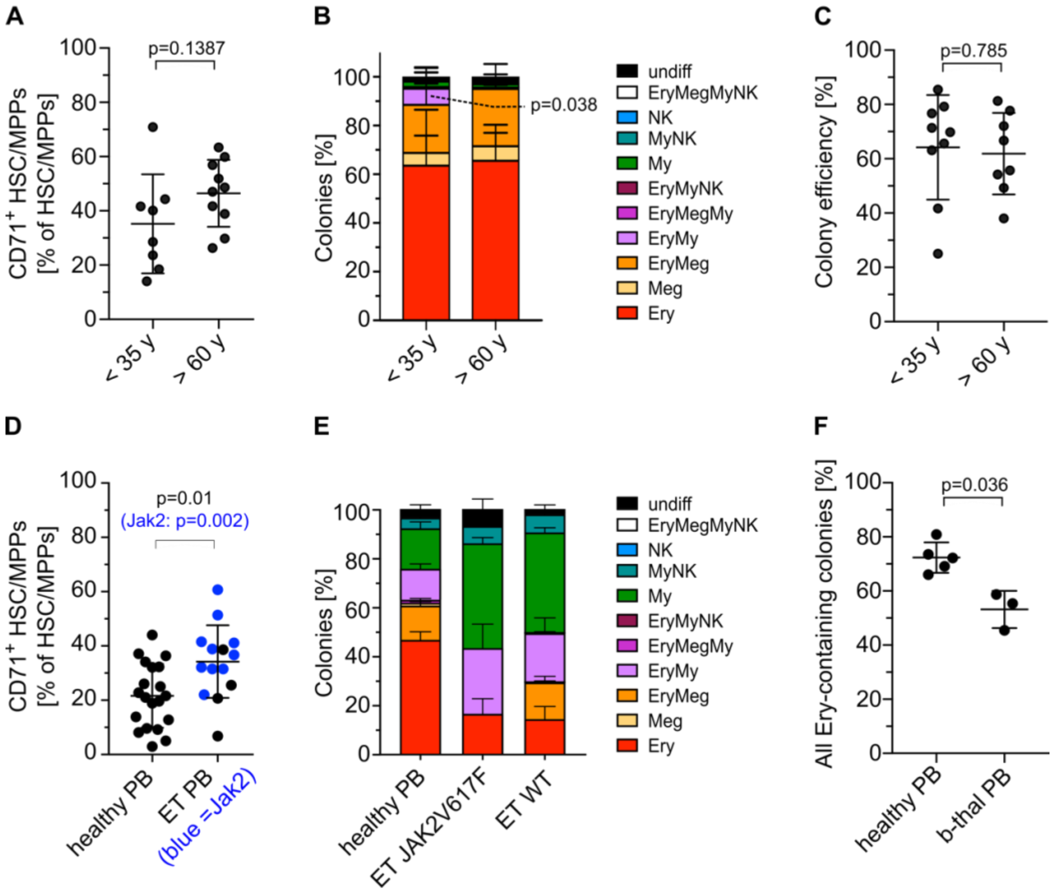

Figure S9. Differentiation output of PB HSC/MPPs in age and disease. Related to Figure 7. **(A)** Percentage of CD71$^+$ HSC/MPPs within the phenotypic HSC/MPP pool of PB from donors <35 years (n=8) and >60 years (n=10). **(B)** Colonies derived from young (<35 years, n=369 single cells from 6 samples) and aged (>60 years, n=209 single cells from 4 samples) CD71$^+$ HSC/MPPs isolated from non-mobilized PB. **(C)** Single cell differentiation assay (MEM) with PB HSC/MPPs from donors <35 years (n=942 single cells; 9 independent PBs) or >60 years (n=700 single cells; 8 independent PBs). Graph shows clonogenic efficiency calculated as the number of colonies per number of seeded cells. **(D)** Percentage of CD71$^+$ HSC/MPPs within the phenotypic HSC/MPP pool PB from healthy donors (n=20) and ET patients (n=13). ET samples carrying the JAK2V617F mutation are highlighted in blue. **(E)** Colonies generated from ET PB HSC/MPPs were genotypes and divided into cells carrying the JAK2V617F mutation (n=35 cells, 3 samples) and wildtype cells (WT, n=180 cells, 3 samples). Same data as in Figure 7F. **(F)** Percentage of all erythroid-containing colonies derived from healthy control PB and b-thal PB. Same samples as in Figure 7G. A-F) Mean± SD is shown. A,C) Two-tailed unpaired t-test. C,D,E,F) Mann-Whitney test.

Figure S10

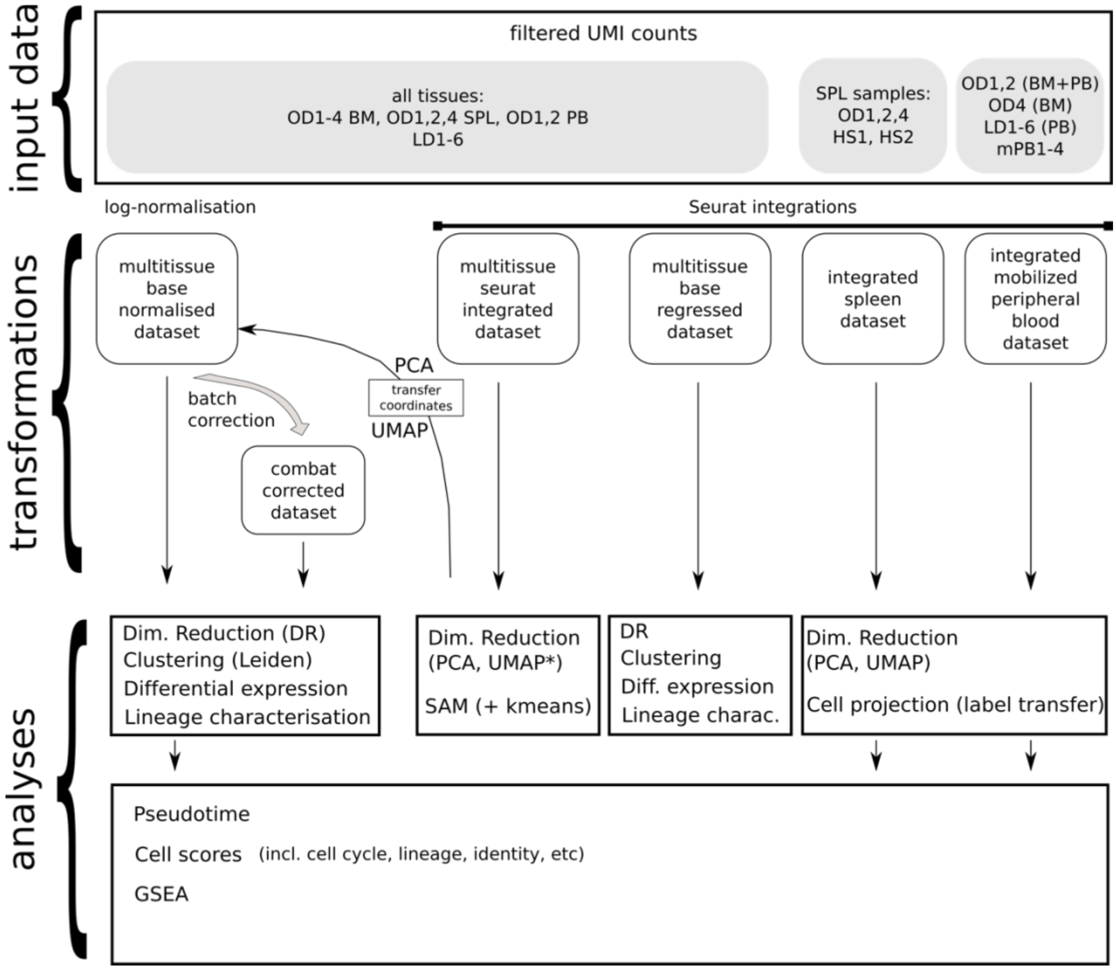

Figure S10. Overview of the 10x mRNA analyses after QC filtering. Grouping of samples (OD1-4 BM, OD1-2,4 SPL, OD1,2 PB, LD1-6 PB; OD1-2,4 SPL, HS1-2 SPL; OD1-2 BM+PB, OD4 BM, LD1-6 PB, mPB1-4) and the respective main pivotal data transformation points which are subsequently used as inputs to produce the listed analyses. The dimensionality reduction step used for the multi-tissue map is highlighted with an asterisk. OD: organ donor, LD: living donor, BM: bone marrow, PB: peripheral blood, mPB: mobilized peripheral blood, SPL: spleen.

Figure S11

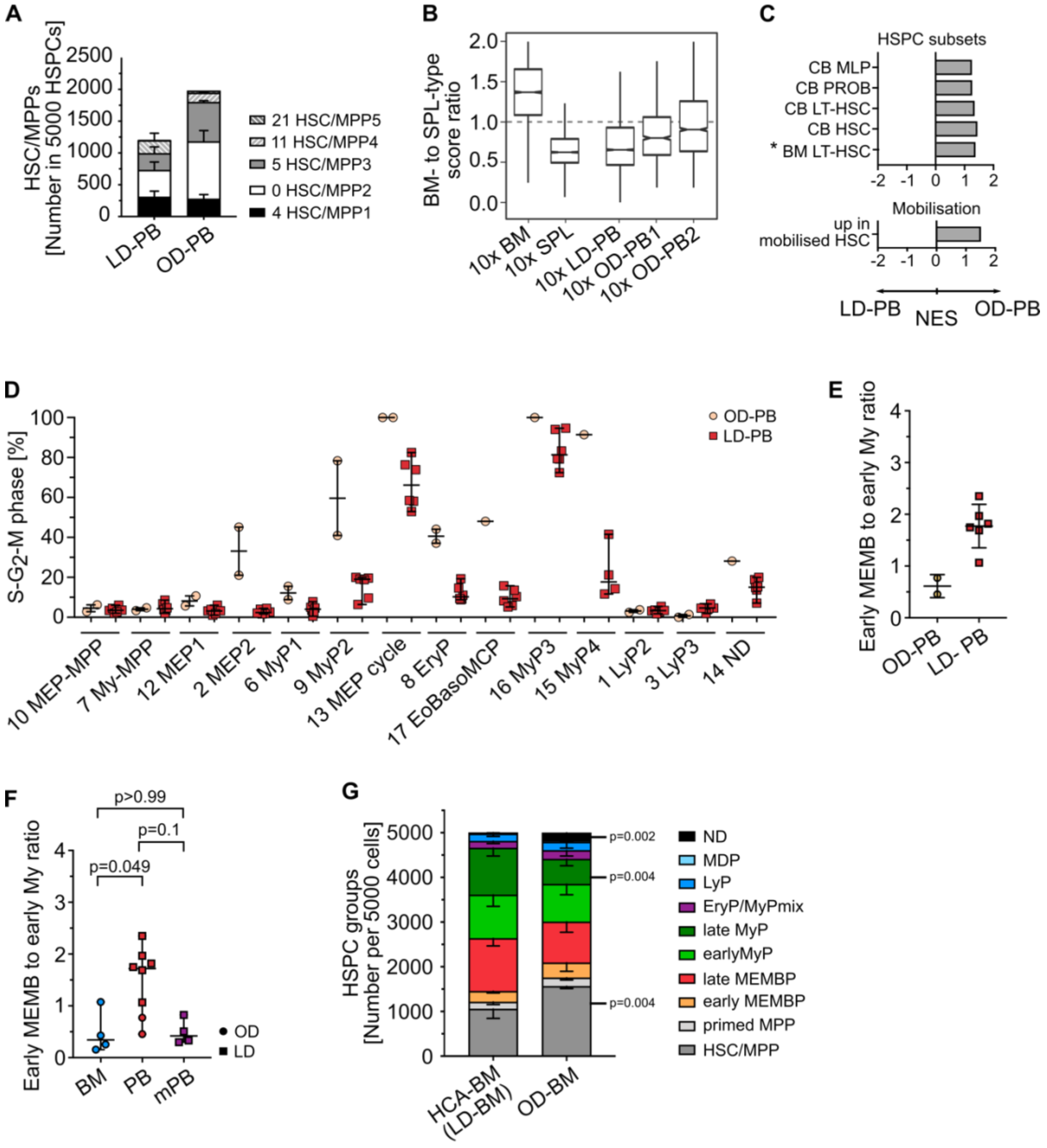

Figure S11. Comparison of living donor samples with organ donor samples. Related to supplementary note 1. **(A)** Composition of HSC/MPP clusters comparing LD-PB (n=6) with OD-PB (n=2). **(B)** Ratio between BM- and SPL-type identity scores in LD-PB and two individual OD-PBs (OD-PB1 and OD-PB2). Notches indicate the 95% confidence interval of the median (middle line). **(C)** Bar graphs show significantly enriched gene sets (FDR <0.05, see Table S3q, S4n-o) in OD-PB compared to LD-PB using population-specific signatures (top, [26,28], BM LT-HSC unpublished) and gene sets that are differentially regulated in mouse HSCs after treatment with mobilizing drugs (middle, [30]). **(D)** Percentage of cells in S-G2-M phase (assigned by cell cycle phase scoring as described by [11]) for each progenitor cluster comparing OD- and LD-PB **(E)** Ratio of early MEMBP to early MyP progenitors in OD- and LD-PB. **(F)** Ratio of

early MEMBP to early MyP progenitors in BM, non-mobilized PB (PB) and mobilized PB (mPB). Kruskal-Wallis test with Dunn’s multiple comparison. **(G)** Comparison of BM from our multi-tissue landscape (OD-BM) to the benchmark HCA-BM reference1 (HCA-BM/LD-BM). Bar graph of the relative composition of HSPC groups are shown. Two-tailed Mann-Whitney test. A,G) Mean ± SD is shown. D,E,F) Median ± 95% CI is shown. LD: living donor, OD: organ donor.

REFERENCES FOR SUPPLEMENTAL MATERIAL

1. Stoeckius M, Hafemeister C, Stephenson W, et al. Simultaneous epitope and transcriptome measurement in single cells. *Nature Methods*. 2017;14(9):865–868.
2. Belluschi S, Calderbank EF, Ciaurro V, et al. Myelo-lymphoid lineage restriction occurs in the human haematopoietic stem cell compartment before lymphoid-primed multipotent progenitors. *Nature Communications*. 2018;9(1):.
3. Nestorowa S, Hamey FK, Sala BP, et al. A single-cell resolution map of mouse hematopoietic stem and progenitor cell differentiation. *Blood*. 2016;128(8):e20–e31.
4. Picelli S, Faridani OR, Björklund ÅK, et al. Full-length RNA-seq from single cells using Smart-seq2. *Nature Protocols*. 2014;9(1):171–181.
5. Zerbino DR, Achuthan P, Akanni W, et al. Ensembl 2018. *Nucleic Acids Res*. 2018;46(Database issue):D754–D761.
6. Wu TD, Nacu S. Fast and SNP-tolerant detection of complex variants and splicing in short reads. *Bioinformatics*. 2010;26(7):873–881.
7. Anders S, Pyl PT, Huber W. HTSeq—a Python framework to work with high-throughput sequencing data. *Bioinformatics*. 2015;31(2):166–169.
8. Brennecke P, Anders S, Kim JK, et al. Accounting for technical noise in single-cell RNA-seq experiments. *Nature Methods*. 2013;10(11):1093–1095.
9. Love MI, Huber W, Anders S. Moderated estimation of fold change and dispersion for RNA-seq data with DESeq2. *Genome Biol*. 2014;15(12):.
10. Leek JT, Johnson WE, Parker HS, Jaffe AE, Storey JD. The sva package for removing batch effects and other unwanted variation in high-throughput experiments. *Bioinformatics*. 2012;28(6):882–883.
11. Satija R, Farrell JA, Gennert D, Schier AF, Regev A. Spatial reconstruction of single-cell gene expression. *Nat Biotechnol*. 2015;33(5):495–502.
12. Anders S, Huber W. Differential expression analysis for sequence count data. *Genome Biology*. 2010;11(10):R106.
13. Heaton H, Talman AM, Knights A, et al. Souporcell: robust clustering of single-cell RNA-seq data by genotype without reference genotypes. *Nat Methods*. 2020;17(6):615–620.
14. Wolock SL, Lopez R, Klein AM. Scrublet: Computational Identification of Cell Doublets in Single-Cell Transcriptomic Data. *Cell Systems*. 2019;8(4):281-291.e9.
15. Wolf FA, Angerer P, Theis FJ. SCANPY: large-scale single-cell gene expression data analysis. *Genome Biol*. 2018;19:.
16. Stuart T, Butler A, Hoffman P, et al. Comprehensive Integration of Single-Cell Data. *Cell*. 2019;177(7):1888-1902.e21.
17. Jardine L, Webb S, Goh I, et al. Blood and immune development in human fetal bone marrow and Down syndrome. *Nature*. 2021;598(7880):327–331.
18. Mulè MP, Martins AJ, Tsang JS. Normalizing and denoising protein expression data from droplet-based single cell profiling. *bioRxiv*. 2021;2020.02.24.963603.
19. Coifman RR, Lafon S. Diffusion maps. *Applied and Computational Harmonic Analysis*. 2006;21(1):5–30.
20. McInnes L, Healy J, Melville J. UMAP: Uniform Manifold Approximation and Projection for Dimension Reduction. *arXiv:1802.03426 [cs, stat]*. 2018;
21. Jacomy M, Venturini T, Heymann S, Bastian M. ForceAtlas2, a Continuous Graph Layout Algorithm for Handy Network Visualization Designed for the Gephi Software. *PLoS One*. 2014;9(6):.
22. Robinson MD, McCarthy DJ, Smyth GK. edgeR: a Bioconductor package for differential expression analysis of digital gene expression data. *Bioinformatics*. 2010;26(1):139–140.

23. Haghverdi L, Büttner M, Wolf FA, Buettner F, Theis FJ. Diffusion pseudotime robustly reconstructs lineage branching. *Nat Methods*. 2016;13(10):845–848.
24. Tarashansky AJ, Xue Y, Li P, Quake SR, Wang B. Self-assembling manifolds in single-cell RNA sequencing data. *eLife*. 2019;8:.
25. Hie B, Bryson B, Berger B. Efficient integration of heterogeneous single-cell transcriptomes using Scanorama. *Nat Biotechnol*. 2019;37(6):685–691.
26. Laurenti E, Doulatov S, Zandi S, et al. The transcriptional architecture of early human hematopoiesis identifies multilevel control of lymphoid commitment. *Nat Immunol*. 2013;14(7):756–763.
27. Subramanian A, Tamayo P, Mootha VK, et al. Gene set enrichment analysis: A knowledge-based approach for interpreting genome-wide expression profiles. *Proceedings of the National Academy of Sciences*. 2005;102(43):15545–15550.
28. Laurenti E, Frelin C, Xie S, et al. CDK6 Levels Regulate Quiescence Exit in Human Hematopoietic Stem Cells. *Cell Stem Cell*. 2015;16(3):302–313.
29. Velten L, Haas SF, Raffel S, et al. Human haematopoietic stem cell lineage commitment is a continuous process. *Nature Cell Biology*. 2017;19(4):271–281.
30. Forsberg EC, Passegué E, Prohaska SS, et al. Molecular Signatures of Quiescent, Mobilized and Leukemia-Initiating Hematopoietic Stem Cells. *PLoS One*. 2010;5(1):.
31. Hay S, Ferchen K, Chetal K, Grimes HL, Salomonis N. The Human Cell Atlas Bone Marrow Single-Cell Interactive Web Portal. *Experimental Hematology*. 2018;68:51–61.
32. Itoh K, Tezuka H, Sakoda H, et al. Reproducible establishment of hemopoietic supportive stromal cell lines from murine bone marrow. *Exp. Hematol.* 1989;17(2):145–153.
33. Baxter EJ, Scott LM, Campbell PJ, et al. Acquired mutation of the tyrosine kinase JAK2 in human myeloproliferative disorders. *Lancet*. 2005;365(9464):1054–1061.
34. Hu Y, Smyth GK. ELDA: Extreme limiting dilution analysis for comparing depleted and enriched populations in stem cell and other assays. *Journal of Immunological Methods*. 2009;347(1):70–78.
35. Mimasaka S. Postmortem Cytokine Levels and the Cause of Death. *The Tohoku Journal of Experimental Medicine*. 2002;197(3):145–150.
36. Schwarz P, Custódio G, Rheinheimer J, et al. Brain Death-Induced Inflammatory Activity is Similar to Sepsis-Induced Cytokine Release. *Cell Transplant*. 2018;27(10):1417–1424.
37. Corr L, Grounds RM, Brown MJ, Whitwam JG. Plasma catecholamine changes during cardiopulmonary bypass: a randomised double blind comparison of trimetaphan camsylate and sodium nitroprusside. *Heart*. 1986;56(1):89–93.
38. Iyer A, Chew HC, Gao L, et al. Pathophysiological Trends During Withdrawal of Life Support: Implications for Organ Donation After Circulatory Death. *Transplantation*. 2016;100(12):2621–2629.
39. Psaila B, Barkas N, Iskander D, et al. Single-cell profiling of human megakaryocyte-erythroid progenitors identifies distinct megakaryocyte and erythroid differentiation pathways. *Genome Biology*. 2016;17:83.
